# Supplementary material for: Ubiquitin-Conjugating Enzyme E2O Primes Hepatocytes to Restore Immune Tolerance in Autoimmune Hepatitis via Inhibiting Y-Box Binding Protein 1/Interleukin-6 Axis
Source: Cell Mol Gastroenterol Hepatol. 2026 Mar 2;20(7):101765. doi: 10.1016/j.jcmgh.2026.101765 (PMC13196572; doi:10.1016/j.jcmgh.2026.101765)
Supplement: Supplementary Table 1 [file mmc1.pdf]

Table S1. Potential proteins showing in mass spectrometry that may interact with Ube2o

Accession	Gene	Description	iBAQ IP [%]
**P99024**	Tubb5	Tubulin beta-5 chain OS=Mus musculus OX=10090 GN=Tubb5 PE=1 SV=1	0.59822702
**P68372**	Tubb4b	Tubulin beta-4B chain OS=Mus musculus OX=10090 GN=Tubb4b PE=1 SV=1	0.844459314
**P11983**	Tcp1	T-complex protein 1 subunit alpha OS=Mus musculus OX=10090 GN=Tcp1 PE=1 SV=3	0.159140979
**P14869**	Rplp0	60S acidic ribosomal protein P0 OS=Mus musculus OX=10090 GN=Rplp0 PE=1 SV=3	0.272813107
**Q6A0A9**	FAM120A	Constitutive coactivator of PPAR-gamma-like protein 1 OS=Mus musculus OX=10090 GN=FAM120A PE=1 SV=2	0.153046016
**Q8CGC7**	Eprs	Bifunctional glutamate/proline--tRNA ligase OS=Mus musculus OX=10090 GN=Eprs PE=1 SV=4	0.030899666
**P70168**	Kpnb1	Importin subunit beta-1 OS=Mus musculus OX=10090 GN=Kpnb1 PE=1 SV=2	0.125655715
**P17742**	Ppia	Peptidyl-prolyl cis-trans isomerase A OS=Mus musculus OX=10090 GN=Ppia PE=1 SV=2	0.596110713
**P09405**	Ncl	Nucleolin OS=Mus musculus OX=10090 GN=Ncl PE=1 SV=2	1.288460477
**Q99KI0**	Aco2	Aconitate hydratase, mitochondrial OS=Mus musculus OX=10090 GN=Aco2 PE=1 SV=1	0.079890581
**Q8C2Q7**	Hnrnph1	Heterogeneous nuclear ribonucleoprotein H OS=Mus musculus OX=10090 GN=Hnrnph1 PE=1 SV=1	0.277162118
**Q5SUT0**	Ewsr1	RNA-binding protein EWS OS=Mus musculus OX=10090 GN=Ewsr1 PE=1 SV=1	0.130512639
**Q61316**	Hspa4	Heat shock 70 kDa protein 4 OS=Mus musculus OX=10090 GN=Hspa4 PE=1 SV=1	0.108820495
**P26039**	Tln1	Talin-1 OS=Mus musculus OX=10090 GN=Tln1 PE=1 SV=2	0.01707066
**Q9JKF1**	Iqgap1	Ras GTPase-activating-like protein IQGAP1 OS=Mus musculus OX=10090 GN=Iqgap1 PE=1 SV=2	0.05028874
**P27659**	Rpl3	60S ribosomal protein L3 OS=Mus musculus OX=10090 GN=Rpl3 PE=1 SV=3	0.551774086
**Q9Z1Q9**	Vars	Valine--tRNA ligase OS=Mus musculus OX=10090 GN=Vars PE=1 SV=1	0.102429248
**Q9JIK5**	Ddx21	Nucleolar RNA helicase 2 OS=Mus musculus OX=10090 GN=Ddx21 PE=1 SV=3	0.140459281

**P08113**	Hsp90b1	Endoplasmin OS=Mus musculus OX=10090 GN=Hsp90b1 PE=1 SV=2	0.204990766
**Q8VEK3**	Hnrnpu	Heterogeneous nuclear ribonucleoprotein U OS=Mus musculus OX=10090 GN=Hnrnpu PE=1 SV=1	0.803984947
**E9PVC6**	Eif4g1	Eukaryotic translation initiation factor 4 gamma 1 OS=Mus musculus OX=10090 GN=Eif4g1 PE=1 SV=1	0.03475399
**Q8BGJ5**	Ptbp1	MCG13402, isoform CRA_a OS=Mus musculus OX=10090 GN=Ptbp1 PE=1 SV=1	0.198017535
**P14148**	Rpl7	60S ribosomal protein L7 OS=Mus musculus OX=10090 GN=Rpl7 PE=1 SV=2	0.338148788
**O35286**	Dhx15	Pre-mRNA-splicing factor ATP-dependent RNA helicase DHX15 OS=Mus musculus OX=10090 GN=Dhx15 PE=1 SV=2	0.101492783
**P11499**	Hsp90ab1	Heat shock protein HSP 90-beta OS=Mus musculus OX=10090 GN=Hsp90ab1 PE=1 SV=3	2.422324743
**Q02053**	Uba1	Ubiquitin-like modifier-activating enzyme 1 OS=Mus musculus OX=10090 GN=Uba1 PE=1 SV=1	0.13755465
**P17182**	Eno1	Alpha-enolase OS=Mus musculus OX=10090 GN=Eno1 PE=1 SV=3	0.553096778
**A0A1B0GQU8**	Rpl18	60S ribosomal protein L18 OS=Mus musculus OX=10090 GN=Rpl18 PE=1 SV=1	1.056936515
**Q9WTI7**	Myo1c	Unconventional myosin-Ic OS=Mus musculus OX=10090 GN=Myo1c PE=1 SV=2	0.022019643
**P26040**	Ezr	Ezrin OS=Mus musculus OX=10090 GN=Ezr PE=1 SV=3	0.443900638
**P45376**	Akr1b1	Aldo-keto reductase family 1 member B1 OS=Mus musculus OX=10090 GN=Akr1b1 PE=1 SV=3	0.029521421
**Q61753**	Phgdh	D-3-phosphoglycerate dehydrogenase OS=Mus musculus OX=10090 GN=Phgdh PE=1 SV=3	0.386099009
**P13020**	Gsn	Gelsolin OS=Mus musculus OX=10090 GN=Gsn PE=1 SV=3	0.26896672
**P10107**	Anxa1	Annexin A1 OS=Mus musculus OX=10090 GN=Anxa1 PE=1 SV=2	0.511040471
**P62960**	Ybx1	Nuclease-sensitive element-binding protein 1 OS=Mus musculus OX=10090 GN=Ybx1 PE=1 SV=3	0.315620703
**P58252**	Eef2	Elongation factor 2 OS=Mus musculus OX=10090 GN=Eef2 PE=1 SV=2	0.867897411

**Q05D44**	Eif5b	Eukaryotic translation initiation factor 5B OS=Mus musculus OX=10090 GN=Eif5b PE=1 SV=2	0.107180357
**P20029**	Hspa5	Endoplasmic reticulum chaperone BiP OS=Mus musculus OX=10090 GN=Hspa5 PE=1 SV=3	0.519680293
**P29341**	Pabpc1	Polyadenylate-binding protein 1 OS=Mus musculus OX=10090 GN=Pabpc1 PE=1 SV=2	0.462381287
**A2BH06**	Rpl11	60S ribosomal protein L11 (Fragment) OS=Mus musculus OX=10090 GN=Rpl11 PE=1 SV=1	0.252306095
**A0A2I3BQF4**	Rpl30	60S ribosomal protein L30 OS=Mus musculus OX=10090 GN=Rpl30 PE=1 SV=1	0.161289031
**P63038**	Hspd1	60 kDa heat shock protein, mitochondrial OS=Mus musculus OX=10090 GN=Hspd1 PE=1 SV=1	0.49364972
**P52293**	Kpna2	Importin subunit alpha-1 OS=Mus musculus OX=10090 GN=Kpna2 PE=1 SV=2	0.0998209
**P52480**	Pkm	Pyruvate kinase PKM OS=Mus musculus OX=10090 GN=Pkm PE=1 SV=4	1.409566132
**B1AXW5**	Prdx1	Peroxiredoxin-1 (Fragment) OS=Mus musculus OX=10090 GN=Prdx1 PE=1 SV=8	0.113624511
**P97429**	Anxa4	Annexin A4 OS=Mus musculus OX=10090 GN=Anxa4 PE=1 SV=4	0.245396353
**P23116**	Eif3a	Eukaryotic translation initiation factor 3 subunit A OS=Mus musculus OX=10090 GN=Eif3a PE=1 SV=5	0.042145191
**P05213**	Tuba1b	Tubulin alpha-1B chain OS=Mus musculus OX=10090 GN=Tuba1b PE=1 SV=2	2.080117936
**P56959**	Fus	RNA-binding protein FUS OS=Mus musculus OX=10090 GN=Fus PE=1 SV=1	0.400415825
**P68369**	Tuba1a	Tubulin alpha-1A chain OS=Mus musculus OX=10090 GN=Tuba1a PE=1 SV=1	0.061917846
**P40142**	Tkt	Transketolase OS=Mus musculus OX=10090 GN=Tkt PE=1 SV=1	0.491205386
**P62908**	Rps3	40S ribosomal protein S3 OS=Mus musculus OX=10090 GN=Rps3 PE=1 SV=1	0.149882137
**P07356**	Anxa2	Annexin A2 OS=Mus musculus OX=10090 GN=Anxa2 PE=1 SV=2	1.057253961
**A0A0R4J0V5**	Polr2a	DNA-directed RNA polymerase subunit OS=Mus musculus OX=10090 GN=Polr2a PE=1 SV=1	0.004397156
**Q60598**	Cttn	Src substrate cortactin OS=Mus musculus OX=10090 GN=Cttn PE=1 SV=2	0.293246049

**P56480**	Atp5f1b	ATP synthase subunit beta, mitochondrial OS=Mus musculus OX=10090 GN=Atp5f1b PE=1 SV=2	0.629389637
**P14206**	Rpsa	40S ribosomal protein SA OS=Mus musculus OX=10090 GN=Rpsa PE=1 SV=4	0.734411361
**P14131**	Rps16	40S ribosomal protein S16 OS=Mus musculus OX=10090 GN=Rps16 PE=1 SV=4	0.259221127
**G3UVV4**	Hk1	Hexokinase 1, isoform CRA_f OS=Mus musculus OX=10090 GN=Hk1 PE=1 SV=1	0.027229461
**A0A075B5N9**	Igkv3-7	Immunoglobulin kappa variable 3-7 OS=Mus musculus OX=10090 GN=Igkv3-7 PE=4 SV=7	0.161966249
**Q6ZQ38**	Cand1	Cullin-associated NEDD8-dissociated protein 1 OS=Mus musculus OX=10090 GN=Cand1 PE=1 SV=2	0.016402436
**Q8BTU6**	Eif4a2	Eukaryotic initiation factor 4A-II OS=Mus musculus OX=10090 GN=Eif4a2 PE=1 SV=1	0.204043718
**A0A0R4J259**	Syncrip	Heterogeneous nuclear ribonucleoprotein Q OS=Mus musculus OX=10090 GN=Syncrip PE=1 SV=1	0.112714499
**Q9JHJ0**	Tmod3	Tropomodulin-3 OS=Mus musculus OX=10090 GN=Tmod3 PE=1 SV=1	0.062642681
**P19096**	Fasn	Fatty acid synthase OS=Mus musculus OX=10090 GN=Fasn PE=1 SV=2	0.069663529
**Q8R0W0**	Eppk1	Epiplakin OS=Mus musculus OX=10090 GN=Eppk1 PE=1 SV=2	0.016306673
**Q03265**	Atp5f1a	ATP synthase subunit alpha, mitochondrial OS=Mus musculus OX=10090 GN=Atp5f1a PE=1 SV=1	1.11084943
**P63017**	Hspa8	Heat shock cognate 71 kDa protein OS=Mus musculus OX=10090 GN=Hspa8 PE=1 SV=1	2.010544351
**P38647**	Hspa9	Stress-70 protein, mitochondrial OS=Mus musculus OX=10090 GN=Hspa9 PE=1 SV=3	0.243666272
**P62137**	Ppp1ca	Serine/threonine-protein phosphatase PP1-alpha catalytic subunit OS=Mus musculus OX=10090 GN=Ppp1ca PE=1 SV=1	0.016440529
**Q61656**	Ddx5	Probable ATP-dependent RNA helicase DDX5 OS=Mus musculus OX=10090 GN=Ddx5 PE=1 SV=2	0.248094644
**P10126**	Eef1a1	Elongation factor 1-alpha 1 OS=Mus musculus OX=10090 GN=Eef1a1 PE=1 SV=3	0.80731813
**P25444**	Rps2	40S ribosomal protein S2 OS=Mus musculus OX=10090 GN=Rps2 PE=1 SV=3	0.325641415
**E9Q390**	Myof	Myoferlin OS=Mus musculus OX=10090 GN=Myof PE=1 SV=2	0.068520723

**E9Q616**	Ahnak	AHNAK nucleoprotein (desmoyokin) OS=Mus musculus OX=10090 GN=Ahnak PE=1 SV=1	0.68425489
**D3YYM6**	Rps5	40S ribosomal protein S5 (Fragment) OS=Mus musculus OX=10090 GN=Rps5 PE=1 SV=1	0.299203453
**E9QAI5**	Cad	CAD protein OS=Mus musculus OX=10090 GN=Cad PE=1 SV=1	0.027160152
**F6TN80**	Srsf11	Serine/arginine-rich-splicing factor 11 (Fragment) OS=Mus musculus OX=10090 GN=Srsf11 PE=1 SV=1	0.019845667
**Q2KN98**	Specc1l	Cytospin-A OS=Mus musculus OX=10090 GN=Specc1l PE=1 SV=1	0.03616345
**C0HKE4**	Hist1h2ae	Histone H2A type 1-E OS=Mus musculus OX=10090 GN=Hist1h2ae PE=1 SV=1	1.111431414
**P62983**	Rps27a	Ubiquitin-40S ribosomal protein S27a OS=Mus musculus OX=10090 GN=Rps27a PE=1 SV=2	0.400198903
**P61979**	Hnrnpk	Heterogeneous nuclear ribonucleoprotein K OS=Mus musculus OX=10090 GN=Hnrnpk PE=1 SV=1	0.382744663
**Q9D8E6**	Rpl4	60S ribosomal protein L4 OS=Mus musculus OX=10090 GN=Rpl4 PE=1 SV=3	0.58050295
**Q9D8N0**	Eef1g	Elongation factor 1-gamma OS=Mus musculus OX=10090 GN=Eef1g PE=1 SV=3	0.373141921
**P12970**	Rpl7a	60S ribosomal protein L7a OS=Mus musculus OX=10090 GN=Rpl7a PE=1 SV=2	0.441445722
