# Supplementary material for: Ubiquitin-Conjugating Enzyme E2O Primes Hepatocytes to Restore Immune Tolerance in Autoimmune Hepatitis via Inhibiting Y-Box Binding Protein 1/Interleukin-6 Axis
Source: Cell Mol Gastroenterol Hepatol. 2026 Mar 2;20(7):101765. doi: 10.1016/j.jcmgh.2026.101765 (PMC13196572; doi:10.1016/j.jcmgh.2026.101765)
Supplement: Supplementary Material [file mmc2.pdf]

Figure 2F

UBE2O

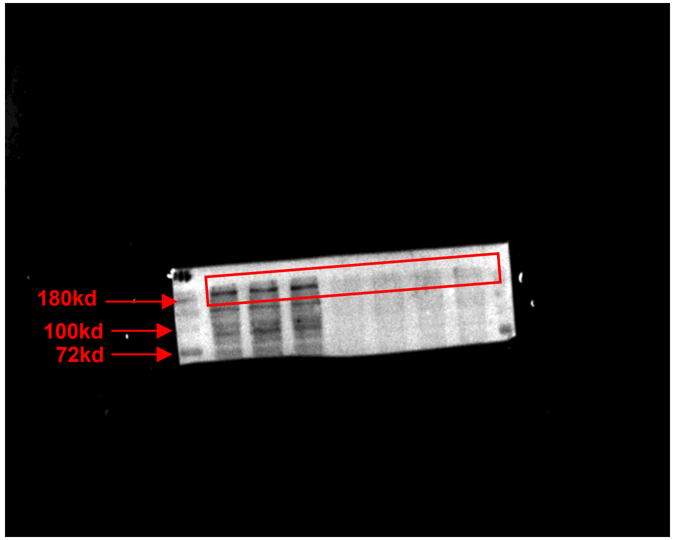

β-actin

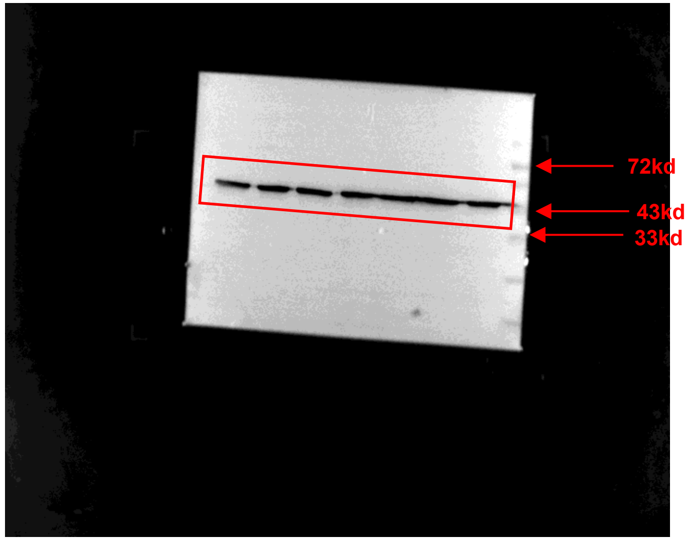

Figure 3B

UBE2O

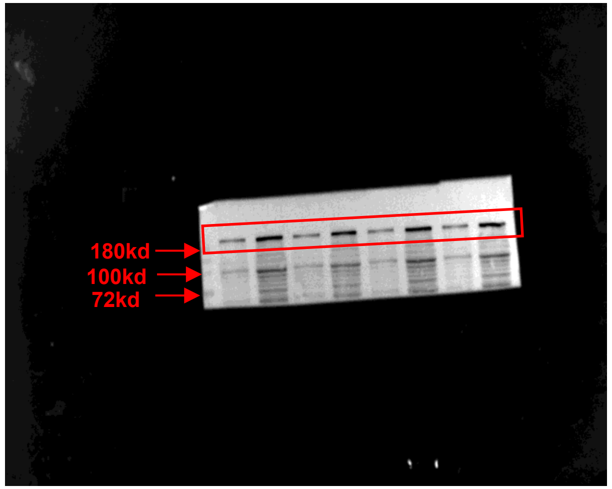

β-actin

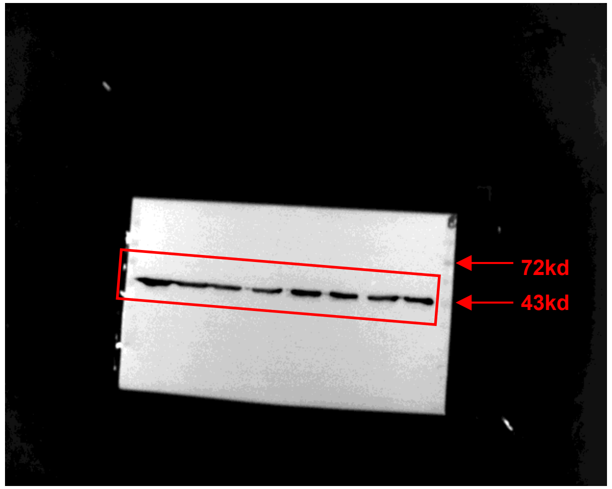

Figure 4B

UBE2O

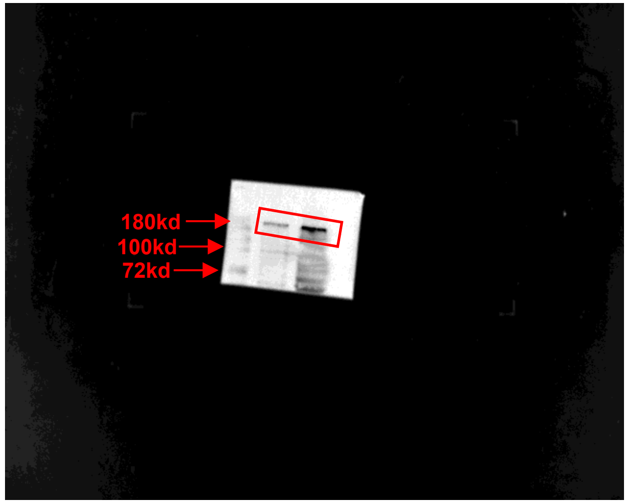

β-actin

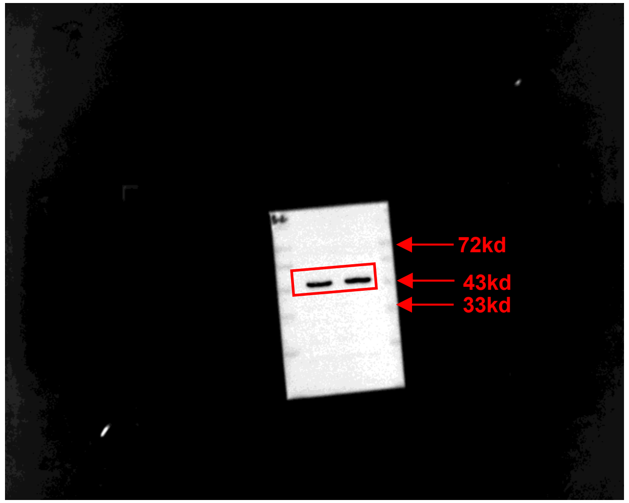

Figure 4G

UBE2O

IL-6

β-actin

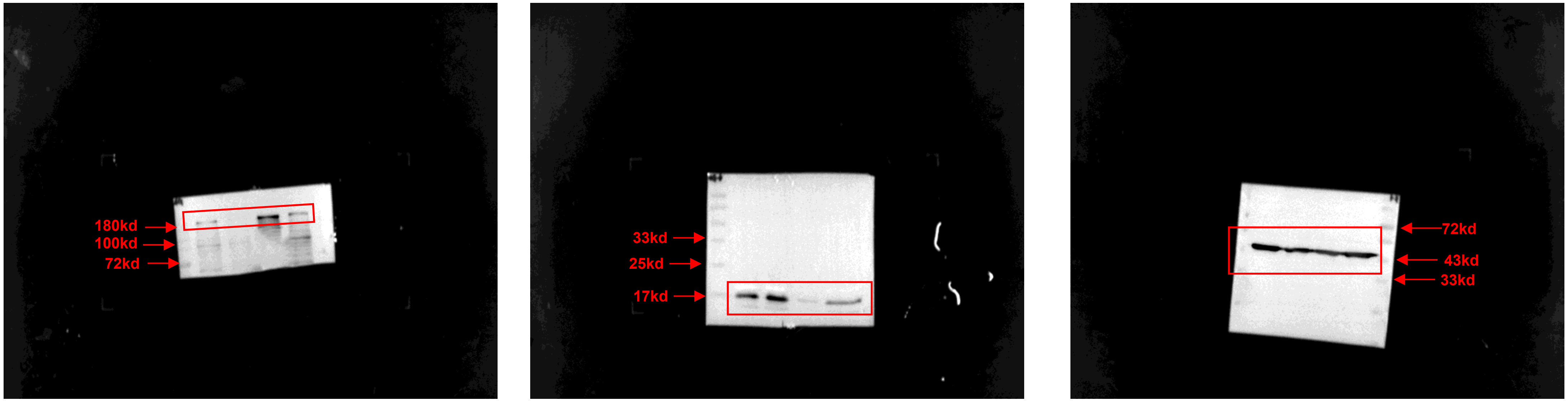

Figure 6B

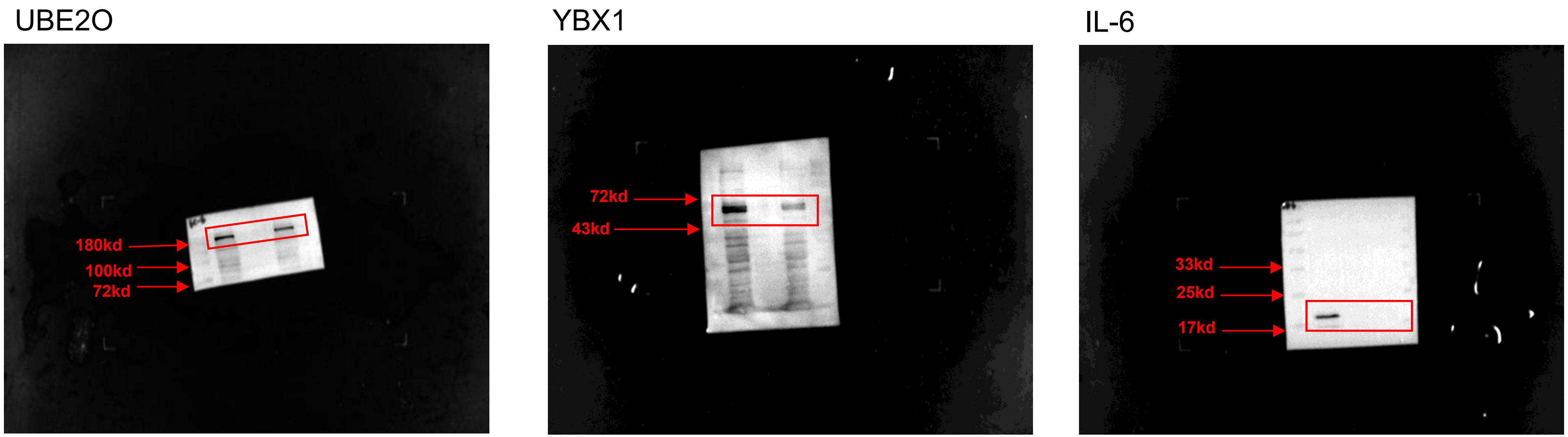

Figure 6C

YBX1

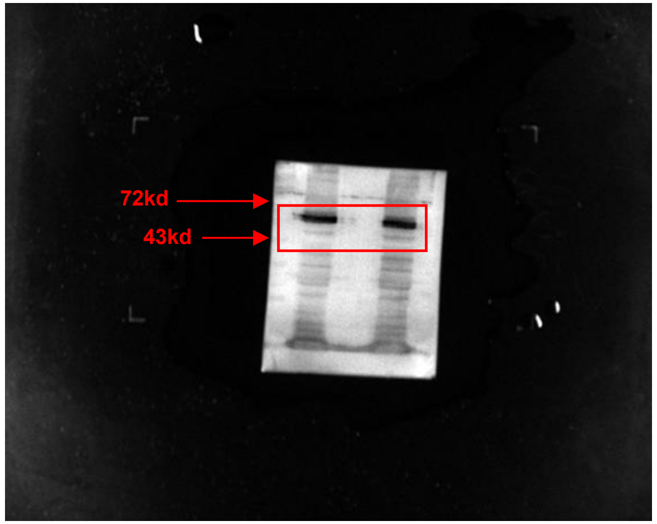

UBE2O

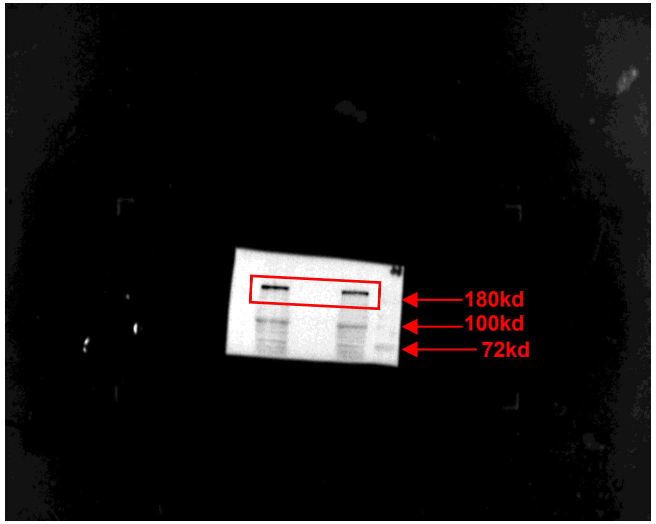

Figure 6D

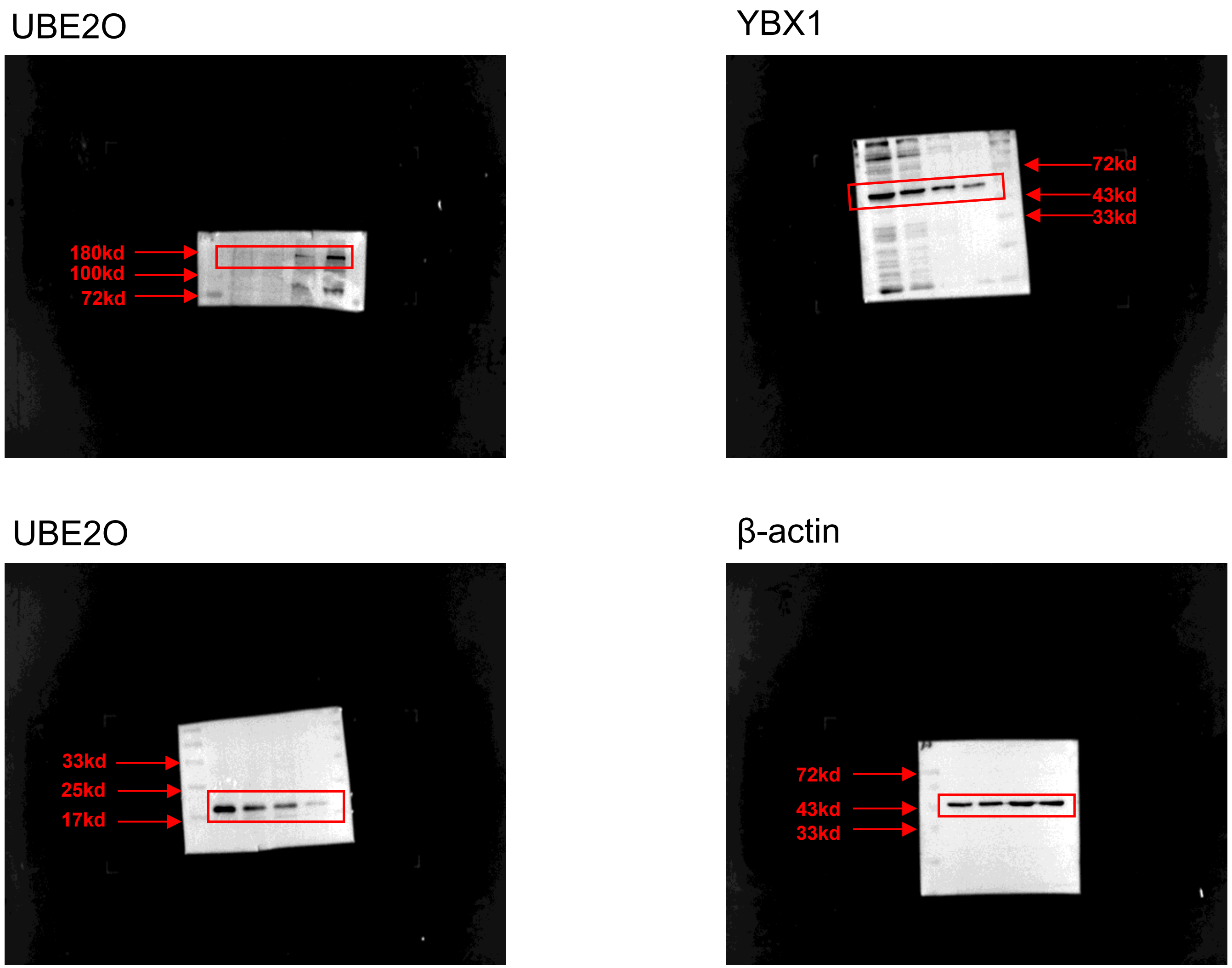
UBE2O
180kd
100kd
72kd
YBX1
72kd
43kd
33kd
UBE2O
33kd
25kd
17kd
β-actin
72kd
43kd
33kd

Figure 6E

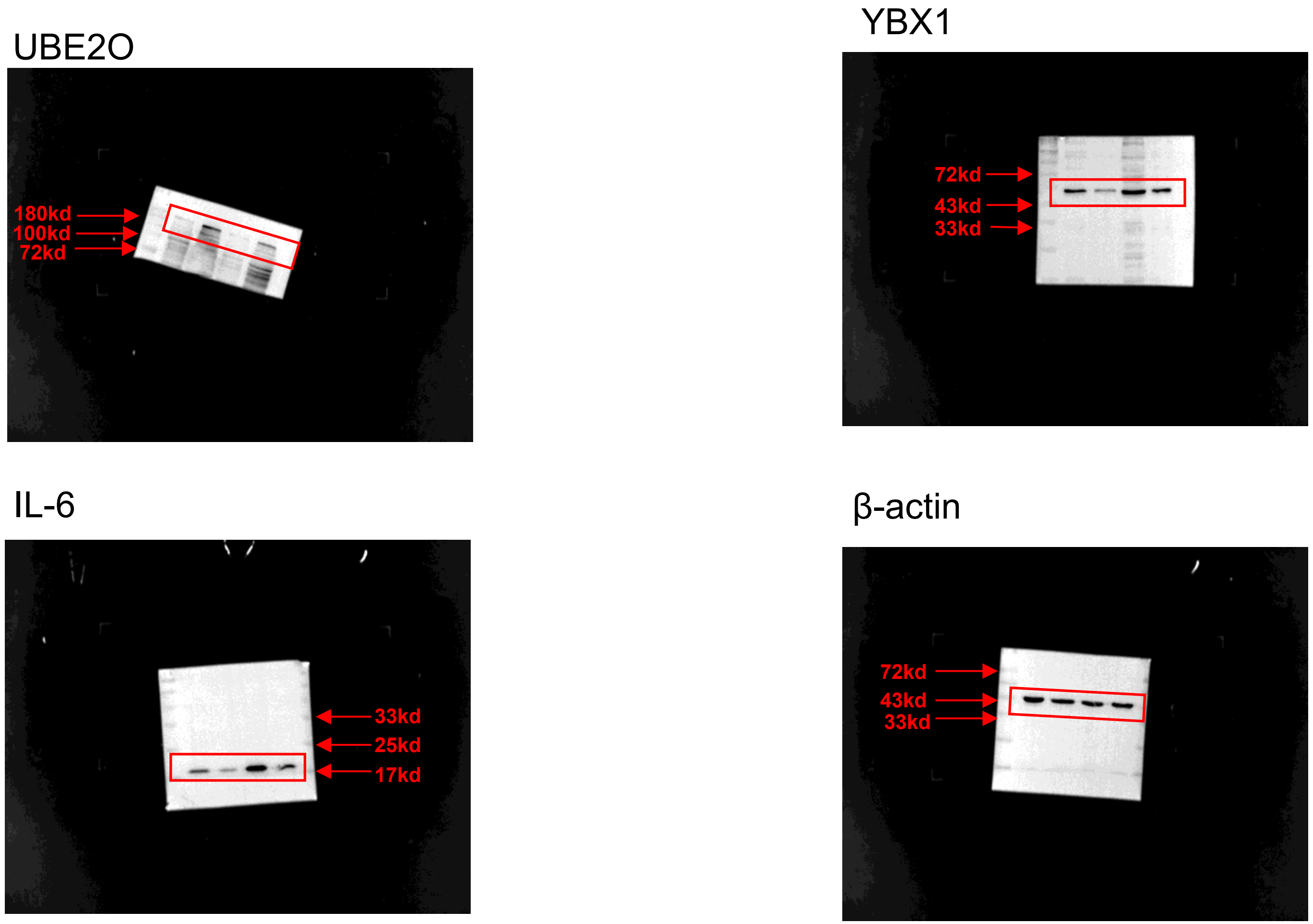

UBE2O
180kd
100kd
72kd
YBX1
72kd
43kd
33kd
IL-6
33kd
25kd
17kd
β-actin
72kd
43kd
33kd

Figure 8E

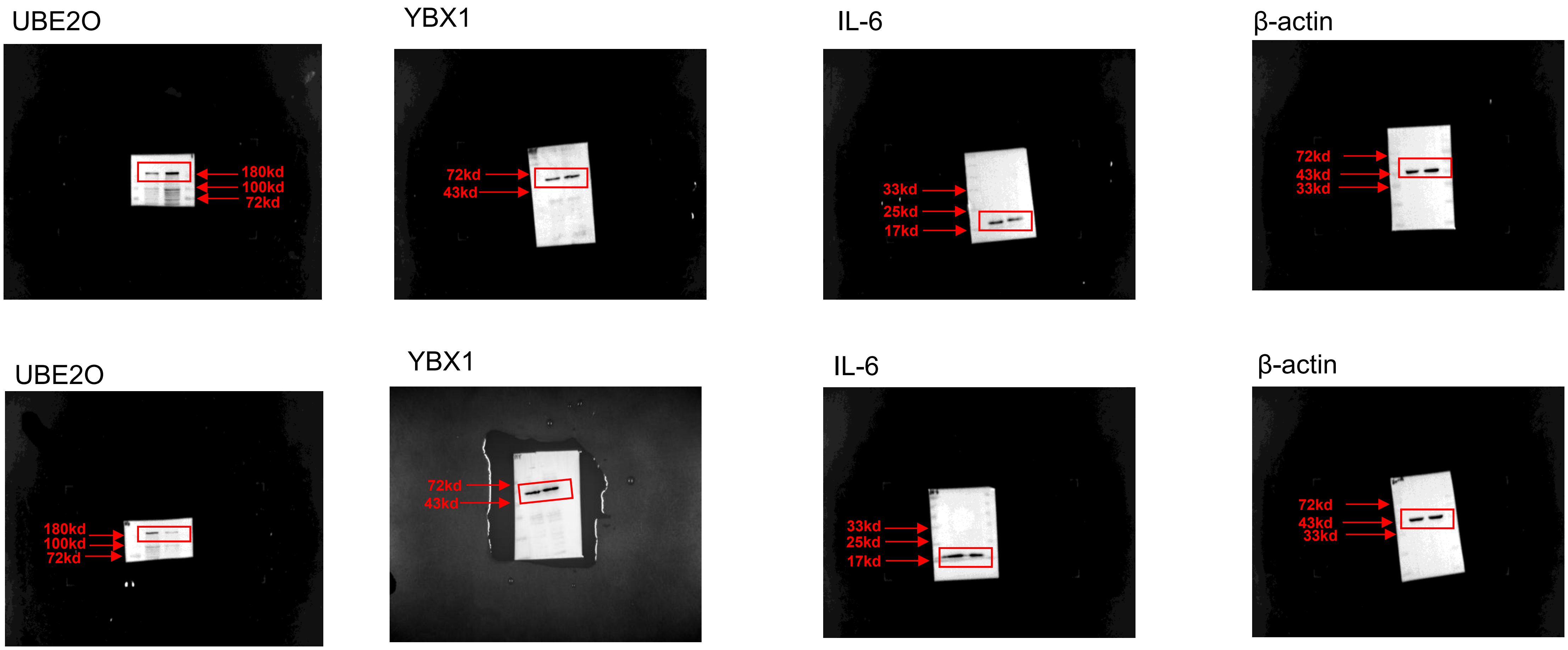
UBE2O
180kd
100kd
72kd
YBX1
72kd
43kd
IL-6
33kd
25kd
17kd
β-actin
72kd
43kd
33kd
UBE2O
180kd
100kd
72kd
YBX1
72kd
43kd
IL-6
33kd
25kd
17kd
β-actin
72kd
43kd
33kd

Figure 9A

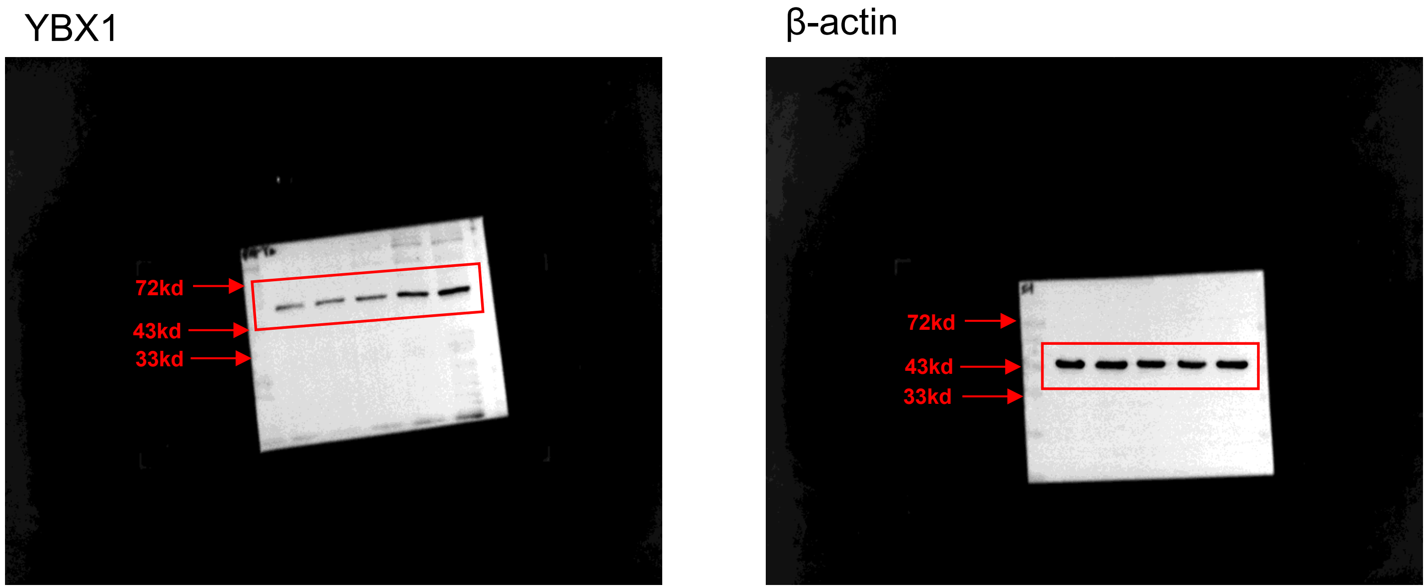

Figure 9B

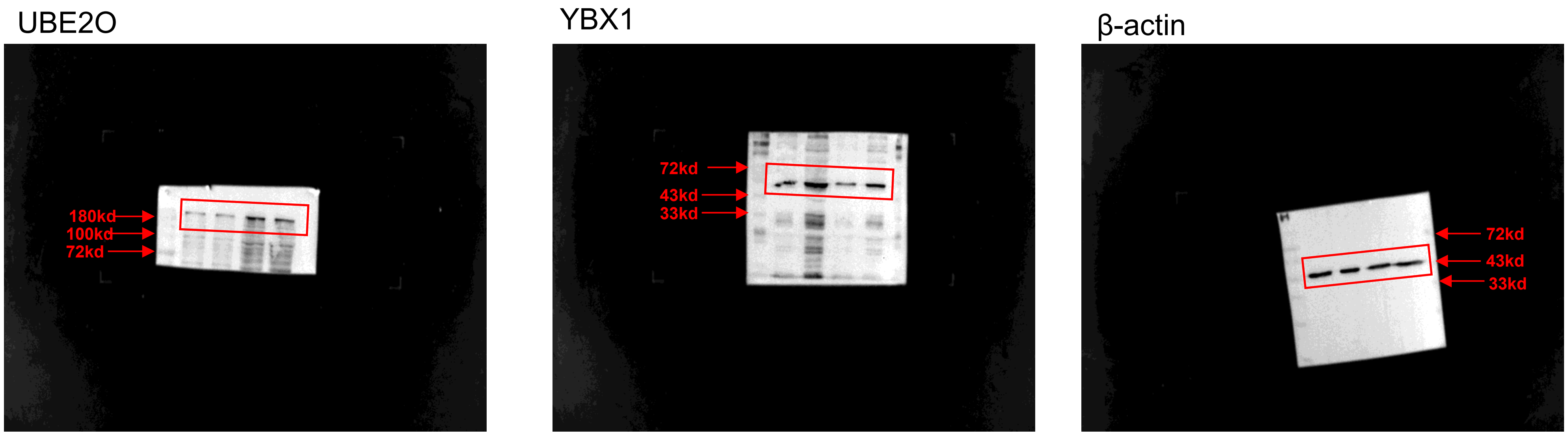
UBE2O
180kd
100kd
72kd
YBX1
72kd
43kd
33kd
β-actin
72kd
43kd
33kd

Figure 9C

UBE2O

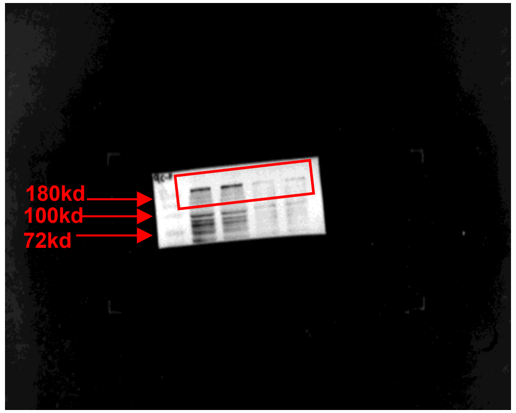

YBX1

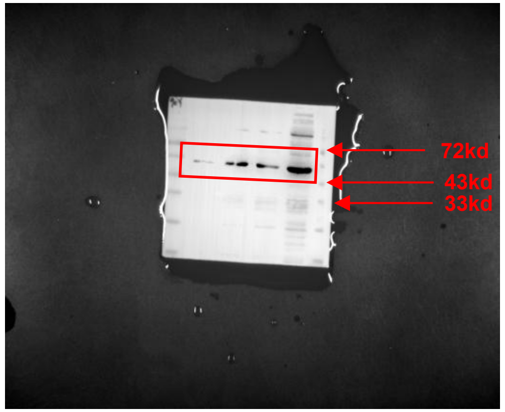

β-actin

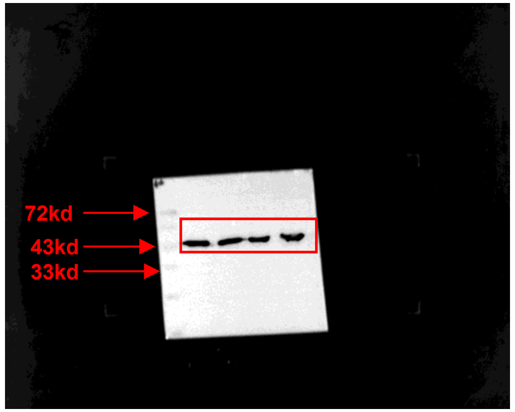

Figure 9D

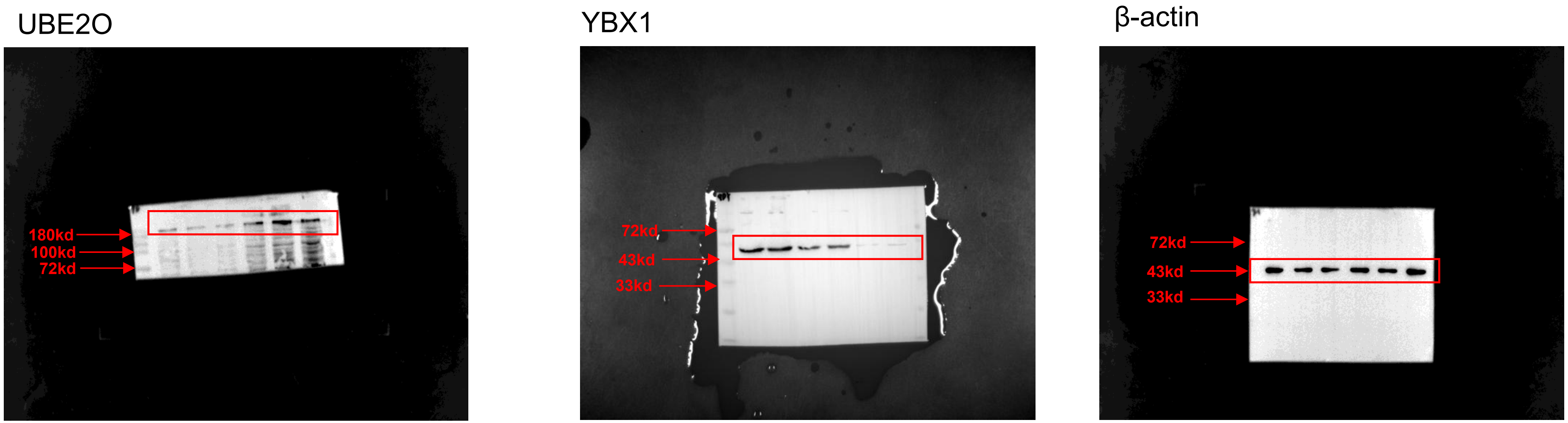
UBE2O
180kd
100kd
72kd
YBX1
72kd
43kd
33kd
β-actin
72kd
43kd
33kd

Figure 9E

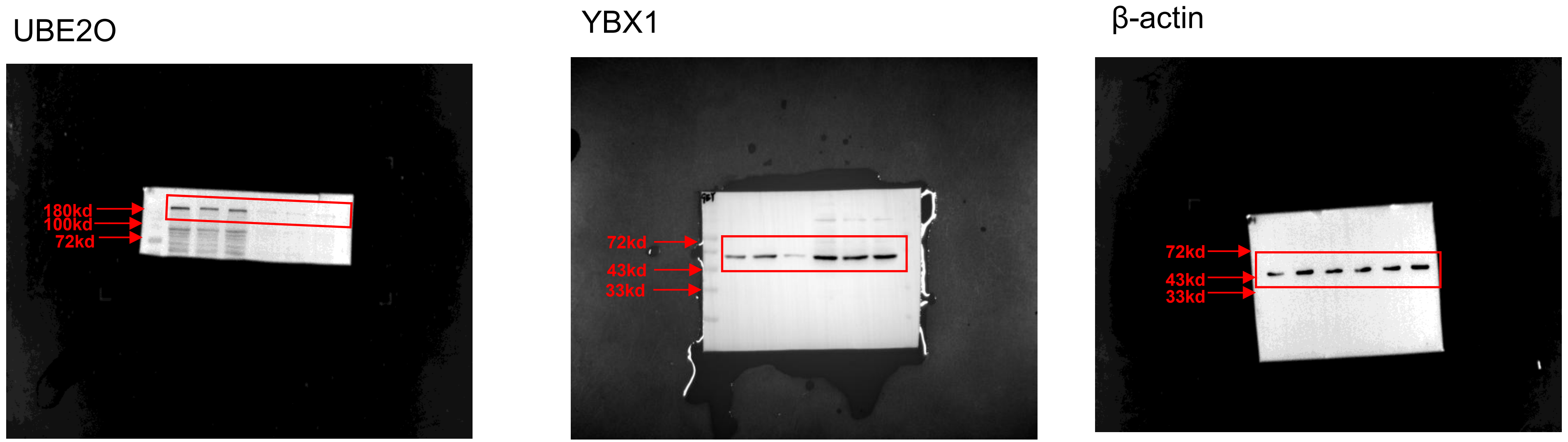
UBE2O
180kd
100kd
72kd
YBX1
72kd
43kd
33kd
β-actin
72kd
43kd
33kd

Figure 9F

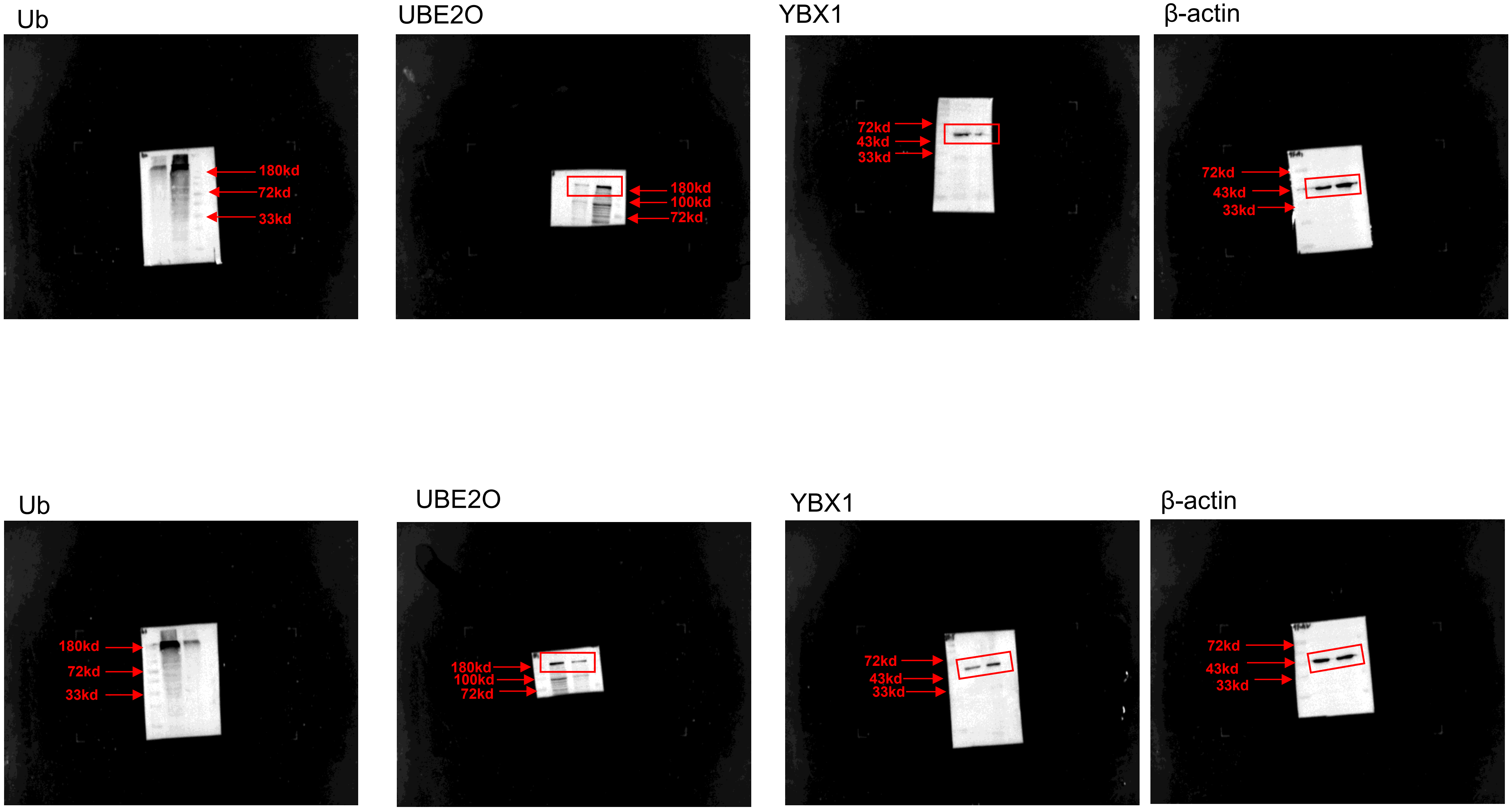
Ub
180kd
72kd
33kd
UBE2O
180kd
100kd
72kd
YBX1
72kd
43kd
33kd
β-actin
72kd
43kd
33kd
Ub
180kd
72kd
33kd
UBE2O
180kd
100kd
72kd
YBX1
72kd
43kd
33kd
β-actin
72kd
43kd
33kd

Figure 9H

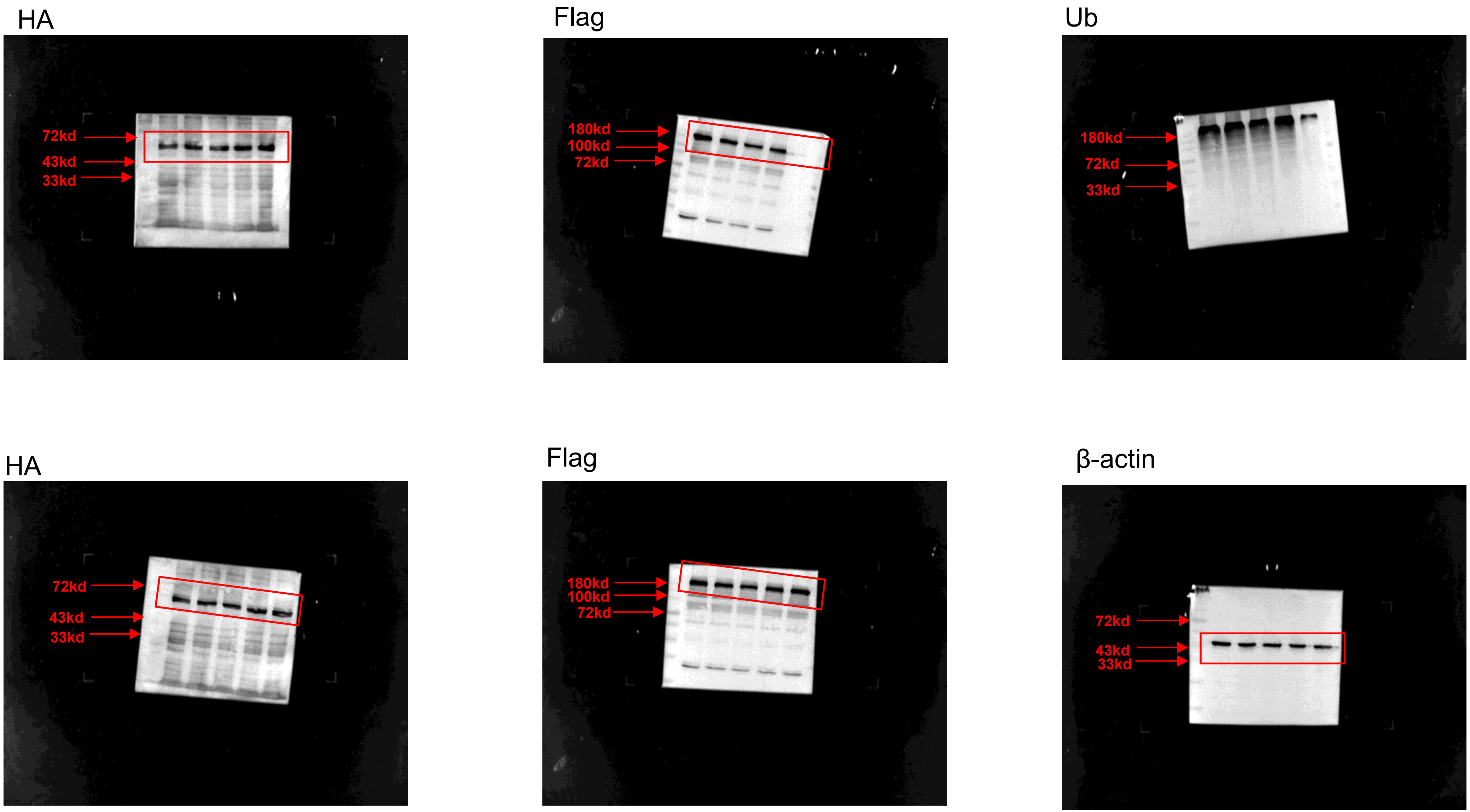
HA
72kd
43kd
33kd
Flag
180kd
100kd
72kd
Ub
180kd
72kd
33kd
HA
72kd
43kd
33kd
Flag
180kd
100kd
72kd
β-actin
72kd
43kd
33kd

Figure 10C

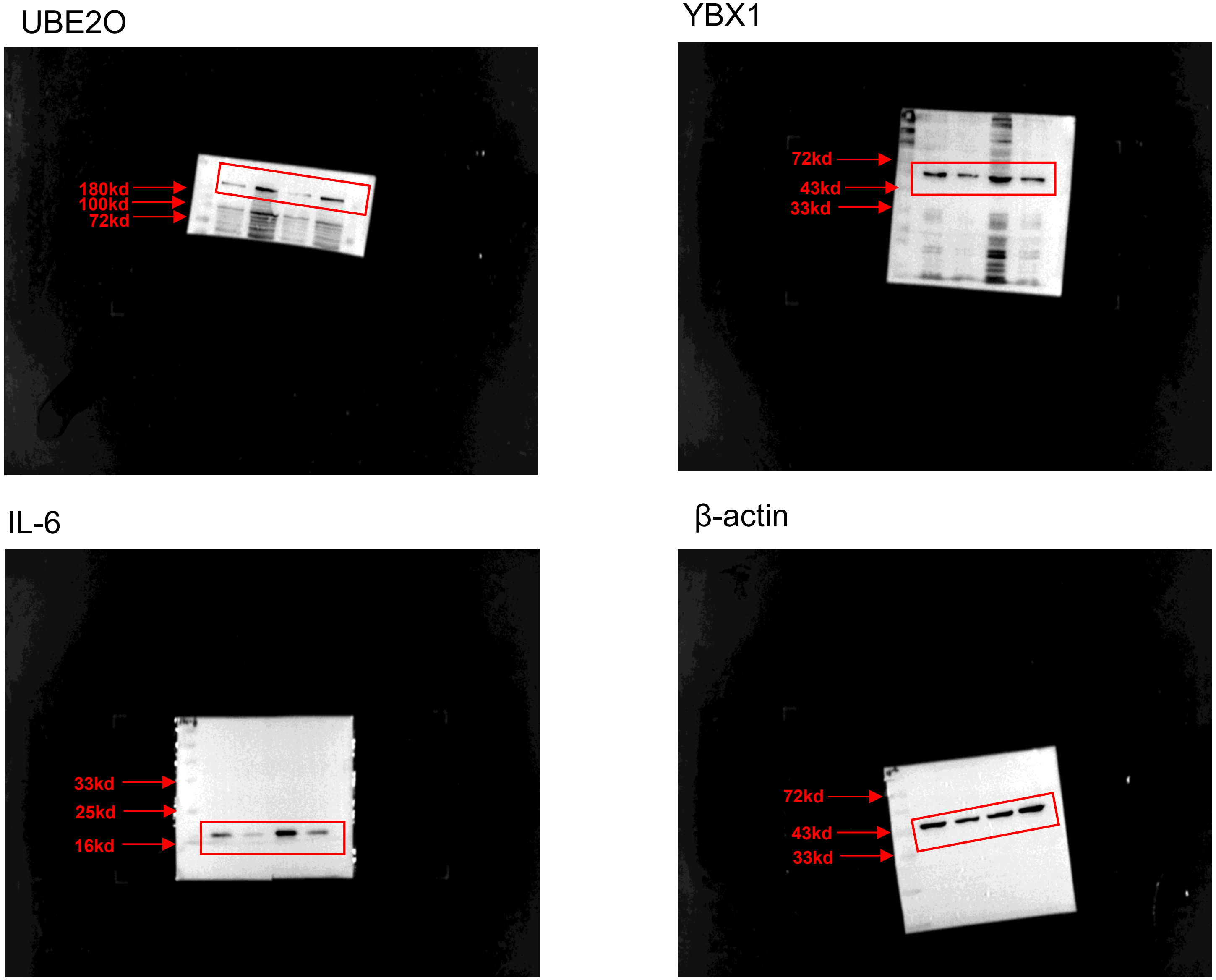
UBE2O
180kd
100kd
72kd
YBX1
72kd
43kd
33kd
IL-6
33kd
25kd
16kd
β-actin
72kd
43kd
33kd
