# Supplementary material for: Ubiquitin-Conjugating Enzyme E2O Primes Hepatocytes to Restore Immune Tolerance in Autoimmune Hepatitis via Inhibiting Y-Box Binding Protein 1/Interleukin-6 Axis
Source: Cell Mol Gastroenterol Hepatol. 2026 Mar 2;20(7):101765. doi: 10.1016/j.jcmgh.2026.101765 (PMC13196572; doi:10.1016/j.jcmgh.2026.101765)
Supplement: Extended PDF [file mmc3.pdf]

cmgh
CELLULAR AND MOLECULAR GASTROENTEROLOGY AND HEPATOLOGY

ORIGINAL RESEARCH

Ubiquitin-Conjugating Enzyme E2O Primes Hepatocytes to Restore Immune Tolerance in Autoimmune Hepatitis via Inhibiting Y-Box Binding Protein 1/Interleukin-6 Axis

Yu Lei,[1,2,*] Han Wang,[1,2,*] Yu Chen,[1,2] Shuhui Wang,[1,2] Zhipeng Du,[1,2] Zheng Huang,[3] Muru Wang,[4] Shangshu Nie,[1,2] Ping Han,[1,2] Wei Yan,[1,2] Mei Liu,[1,2] and Dean Tian[1,2]

[1]Department of Gastroenterology, Tongji Hospital, Tongji Medical College, Huazhong University of Science and Technology, Wuhan, Hubei Province, China; [2]Hubei Key Laboratory of Hepato-Biliary-Pancreatic Diseases, Tongji Hospital, Tongji Medical College, Huazhong University of Science and Technology, Wuhan, Hubei Province, China; [3]Department of Pediatrics, Tongji Hospital, Tongji Medical College, Huazhong University of Science and Technology, Wuhan, Hubei Province, China; and [4]Department of Gastroenterology, Henan Provincial People's Hospital, Zhengzhou University People's Hospital, Zhengzhou, Henan, China

Summary

This study provides evidence to demonstrate the hepatoprotective role of UBE2O in AIH and sheds light on an important mechanistic link between the hepatocellular UBE2O/YBX1/IL-6 axis and hepatic Treg/Th17 immune homeostasis.

UBE2O overexpression promotes ubiquitination degradation of YBX1 and inhibits IL-6 secreted by hepatocytes, thus restoring hepatic Treg/Th17 balance and attenuating liver injury in AIH.

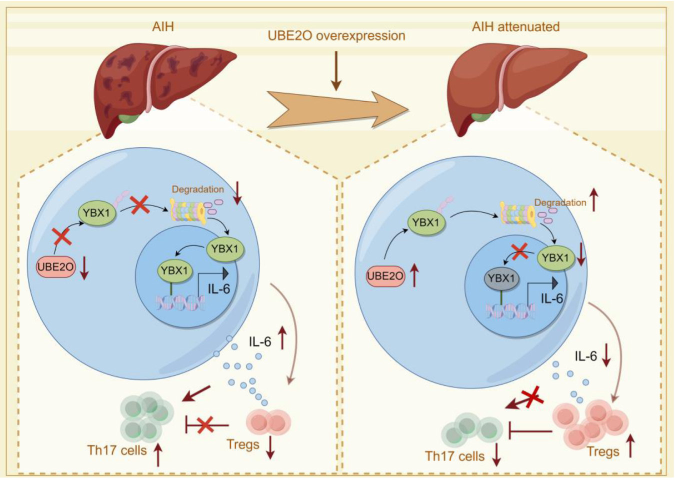

SUMMARY

We uncover a previously unrecognized hepatocyte-intrinsic mechanism in which ubiquitin-conjugating enzyme E2O promotes K135-specific ubiquitination and degradation of Y-box binding protein 1, leading to suppressed interleukin-6 production, reprogrammed CD4^{+} T cell differentiation toward regulatory T cells, and restoration of the regulatory T cell/T helper cell 17 balance.

WHAT YOU NEED TO KNOW

Background: Autoimmune hepatitis (AIH) is a chronic progressive inflammatory liver disease, with its incidence increasing worldwide. The mechanisms underlying the regulation of immune tolerance in AIH remain largely unknown.

Impact: Our study revealed a previously unrecognized hepatocellular ubiquitin-conjugating enzyme E2O/Y-box binding protein 1/interleukin-6 axis in AIH that primes hepatocytes to restore immune tolerance.

Future Directions: Targeting ubiquitin-conjugating enzyme E2O might provide a promising therapeutic target for AIH by linking post-translational modification and hepatic immune tolerance.

BACKGROUND & AIMS: Autoimmune hepatitis (AIH) is a chronic progressive inflammatory liver disease, with its incidence increasing continuously worldwide. The mechanisms underlying the regulation of immune tolerance in AIH remain largely unknown. This study investigated a novel regulatory pathway of regulatory T cell/T helper 17 (Treg/Th17) balance involving the ubiquitin-conjugating enzyme E2O (UBE2O) and underlying mechanisms.

METHODS: UBE2O expression was analyzed in patients and mice with AIH. In vivo UBE2O overexpression in a chronic AIH model and naïve CD4$^+$ T differentiation induction assays were used to investigate the exact role of UBE2O in AIH. Liver samples were assessed by histology, immunochemistry, immunoblot, flow cytometry, and enzyme-linked immunosorbant assays. Mass spectrometry, co-immunoprecipitation, cytokine microarray, and site-specific mutation experiments were utilized to elucidate underlying molecular mechanisms.

RESULTS: We detected a reduction of UBE2O expression in livers of patients and mice with AIH. Low expression of UBE2O indicated severe liver inflammatory injury. Hepatic UBE2O overexpression experiments demonstrated that UBE2O alleviated hepatic injury, inflammation, and fibrosis, and restored Treg/Th17 balance in experimental AIH. Mechanistically, UBE2O was found to interact with and promote ubiquitination degradation of YBX1 at lysine 135 (K135), leading to the reduction of interleukin-6 transcription and secretion in hepatocytes, thus rewiring naïve CD4$^+$ T cells differentiation into Tregs. Furthermore, YBX1 partially reversed the protective effects of UBE2O overexpression in AIH.

CONCLUSIONS: Our study revealed a previously unrecognized hepatocellular UBE2O/YBX1/interleukin-6 axis in AIH that primes hepatocytes to restore immune tolerance. Targeting UBE2O might provide a promising therapeutic target for AIH by linking posttranslational modification and hepatic immune tolerance. *(Cell Mol Gastroenterol Hepatol 2026;20:101765; https://doi.org/10.1016/j.jcmgh.2026.101765)*

Keywords: Autoimmune Hepatitis (AIH); Immune Tolerance; Ubiquitin-Conjugating Enzyme E2O (UBE2O); Y-Box-Binding Protein 1 (YBX1).

Autoimmune hepatitis (AIH) is a chronic, progressive inflammatory liver disease that is increasingly recognized as a global health concern, with a significantly rising incidence.[1–3] Immune disorders are a critical factor in the pathogenesis of AIH, and immunosuppressive therapy is the standard clinical intervention.[1] However, a substantial number of patients do not respond favorably to this treatment due to drug resistance or adverse side effects.[2] Therefore, exploring the cellular and molecular underlying immune disorders in the liver and identifying novel therapeutic targets might improve the outcomes and prognoses of patients with AIH.

The liver is acknowledged as a pivotal immune organ, where various immune cells interact synergistically within the hepatic microenvironment.[4] AIH is characterized by T cell-mediated immune liver injury, with CD4$^+$ T cells playing a critical role.[1] Foxp3$^+$ regulatory T cells (Tregs) are an immunosuppressive subset of CD4$^+$ T cells that modulate autoreactive T cells and mitigate proinflammatory processes by secreting anti-inflammatory cytokines, thereby maintaining peripheral immune tolerance.[5] It is proposed that a reduction in the quantity or functional capacity of Tregs may contribute to the progression of AIH.[6,7] T helper cell 17 (Th17) cells, another subset of CD4$^+$ T cells, are known for their ability to produce proinflammatory cytokines, particularly interleukin (IL)-17, thereby promoting immune cell infiltration and hepatic inflammatory damage. Notably, the proliferation and functions of Th17 cells can be effectively inhibited by Tregs.[8] A growing body of evidence suggests that an imbalance between Tregs and Th17 cells is implicated in the pathogenesis and progression of various autoimmune diseases, including AIH.[9–11] However, a thorough understanding of the factors influencing Treg/Th17 homeostasis in AIH remains elusive. In this regard, exploring potential therapeutic targets that regulate the Treg/Th17 balance might hold significant promise for AIH treatment.

Ubiquitination, a critical form of post-translational modification, plays an essential role in autoimmune diseases; however, its exact role in AIH has yet to be elucidated.[12] In recent years, protein ubiquitination has emerged as a pivotal regulator of the immune system, particularly in the development and differentiation of immune cells, including T cells.[13] The ubiquitin-conjugating enzyme E2O (UBE2O) is a novel ubiquitin-binding enzyme that exhibits dual functionality, possessing both ubiquitin-conjugating enzyme E2 and ubiquitin ligase E3 activities. UBE2O has been reported to be implicated in a variety of biological processes, such as metabolism, DNA repair, and circadian rhythm regulation.[14–16] Notably, a previous study has observed that UBE2O could bind to tumor necrosis factor receptor-associated factor 6 (TRAF6) to block the activation of nuclear transcription factor-κB (NF-κB) and secretion of several inflammatory cytokines,[17] suggesting a potential role for UBE2O in immunoregulation.

***Authors share co-first authorship.**

Abbreviations used in this paper: **ACLF, acute-on-chronic liver failure; AIH, autoimmune hepatitis; ALT, alanine aminotransferase; ANOVA, non-parametric one-way analysis of variance; aPC, activated Protein C; APC, allophycocyanin; AST, aspartate aminotransferase; CBA, cytometric bead array; CHX, cycloheximide; Co-IP, co-immunoprecipitation; conA, concanavalin A; CTLA4, cytotoxic T-lymphocyte associated protein 4; CYP2D6, cytochrome P450 2D6; DAPI, 4,6-diamidino-2-phenylindone; DMEM, Dulbecco's Modified Eagle's Medium; EDTA, ethylenediaminetetraacetic acid; ELISA, enzyme-linked immunosorbent assay; FACS, fluorescence-activated cell sorting; FBS, fetal bovine serum; FITC, fluorescein isothiocyanate; Foxp3, forkhead box p3; H&E, hematoxylin and eosin; HCC, hepatocellular carcinoma; IF, immunofluorescence; IFN-γ, interferon-γ; IHC, immunohistochemistry; IL, interleukin; IP, immunoprecipitation; K48, lysine 48; K48-Ub, K48-linked ubiquitination; Knodell HAI, Knodell histological activity index; LPS, lipopolysaccharide; MNC, mononuclear cell; mRNA, messenger RNA; MS, mass spectrometry; NAFLD, nonalcoholic fatty liver disease; NF-κB, nuclear transcription factor-κB; OTUB1, Otubain1; PBS, phosphate-buffered saline; PE, phycoerythrin; qRT-PCR, quantitative real-time-polymerase chain reaction; SDS-PAGE, sodium dodecyl-sulfate polyacrylamide gel electrophoresis; SEM, standard error of the mean; SPF, specific pathogen-free; TGFβ, transforming growth factor β; Th17, T helper cell 17; TNF-α, tumor necrosis factor-α; TRAF6, tumor necrosis factor receptor-associated factor 6; Treg, regulatory T cell; Ub, ubiquitination; UBE2O, ubiquitin-conjugating enzyme E2O; YBX1, Y-box binding protein 1.**

Most current article

© 2026 The Authors. Published by Elsevier Inc. on behalf of American Gastroenterological Association Institute. This is an open access article under the CC BY license (http://creativecommons.org/licenses/by/4.0/).
2352-345X
https://doi.org/10.1016/j.jcmgh.2026.101765

Despite these findings, it remains unknown whether UBE2O could effectively modulate Treg/Th17 homeostasis within the liver microenvironment and thereby exert an immunoregulatory effect in AIH. Based on experimental results, we demonstrated a novel hepatoprotective role of UBE2O in AIH and revealed its role in priming hepatocytes to restore immune tolerance. Mechanistically, we illustrated a previously unrecognized hepatocellular UBE2O/Y-box binding protein 1 (YBX1)/IL-6 axis that restores the Treg/Th17 balance in AIH. This work provides a promising therapeutic target for AIH by linking post-translational modification with hepatic immune tolerance.

Results

UBE2O Is Significantly Decreased in Hepatic Tissues of AIH

Although dysregulation of UBE2O expression has been documented in hepatocellular carcinoma (HCC),[14] its expression pattern in AIH has not been previously characterized. In this study, we assessed the hepatic expression levels of UBE2O in patients with AIH. The diagnosis of AIH was pathologically confirmed, and typical pathological characteristics detected in patients with AIH, such as interface hepatitis, lymphocytes invasion, and rosettes, are displayed in Figure 1*A*. As shown in Figure 1*B*, the results of immunohistochemistry (IHC) staining of liver sections exhibited a marked reduction of hepatic UBE2O expression in patients with AIH compared with controls. Based on liver biopsy findings, patients with AIH were divided into 3 groups: absent or only portal inflammation (G0–G1), mild interface hepatitis (G2), and moderate or severe interface hepatitis (G3–G4). We found a significant difference of IHC intensity for UBE2O expression across different grades of inflammation (Figure 1*C*). Moreover, when comparing the level of alanine aminotransferase (ALT) and aspartate aminotransferase (AST), we also found a difference between patients with AIH with relatively high or low UBE2O expression (Figure 1*D*). Then, a chronic AIH murine model was employed to analyze the expression level of UBE2O, wherein the hepatic histological alterations and antibody production closely mirrored those observed in patients with AIH.[18] Hematoxylin and eosin (H&E) staining results indicated a higher degree of inflammatory cell infiltration after modeling (Figure 1*E*). The results of Western blotting analysis suggested that the expression level of hepatic UBE2O was dramatically decreased in liver tissues of AIH models compared with the controls (Figure 1*F*). The IHC and immunofluorescence (IF) staining results of liver sections also revealed a marked reduction of hepatic UBE2O expression in experimental AIH (Figure 1*G* and *H*), with notable abundant expression in hepatocytes. To further determine the expression of UBE2O in hepatocytes, primary hepatocytes were isolated from mice with both control and chronic AIH. IF staining results revealed a notable reduction in hepatocellular UBE2O expression in those with AIH, as evidenced by the decreased positive staining and diminished intensity of UBE2O in hepatocytes (Figure 1*I*). These findings indicated that UBE2O was significantly downregulated in hepatic tissues of AIH.

UBE2O Overexpression Ameliorates AIH and Restores Hepatic Treg/Th17 Balance

To investigate the potential role of UBE2O in AIH in mice, UBE2O was overexpressed using a hydrodynamics-based gene delivery method (Figure 2*A* and *B*). As shown in Figure 2*C*, hepatic overexpression of UBE2O markedly mitigated liver inflammation, as evidenced by the alleviation of interface hepatitis and reduced liver inflammatory scores in chronic AIH mice. Meanwhile, alterations in inflammatory factors in the serum of chronic AIH mice were assessed, revealing that UBE2O overexpression led to a significant decrease in serum levels of the proinflammatory factor IL-17A, which was involved in Th17 cell differentiation, as well as tumor necrosis factor-α (TNF-α) and interferon-γ (IFN-γ), while the serum anti-inflammatory factor IL-10 level was increased after UBE2O overexpression (Figure 2*D*). Moreover, Sirius red staining results demonstrated that UBE2O overexpression significantly mitigated liver fibrosis in chronic AIH (Figure 2*E*). Collectively, these results suggested that UBE2O exerted a protective effect against AIH through restoring the balance between anti-inflammatory and proinflammatory responses.

As mentioned above, our study revealed a reduction in Th17-related proinflammatory factors following UBE2O expression, whereas IL-10, an anti-inflammatory cytokine predominantly secreted by Tregs, was elevated with UBE2O overexpression. A negative correlation was observed between the Treg/Th17 ratio and liver inflammatory scores in liver sections from control and chronic AIH mice at various stages of hepatitis (Figure 2*F*), further underscoring the significance of Treg/Th17 imbalance in AIH. Therefore, we hypothesized that UBE2O modulated the expression of key factors to enhance the proportion of Tregs and regulate the hepatic Treg/Th17 balance, thereby re-establishing immune tolerance in the liver. We analyzed the changes in the proportions of both Treg and Th17 cells in the liver. In the chronic AIH model, a decrease in the Treg/Th17 ratio was detected, suggesting a compromise in immune tolerance associated with chronic AIH. Nevertheless, overexpression of UBE2O resulted in an elevation of the Treg/Th17 ratio in the liver, as illustrated in Figure 2*G*. Consistently, IHC staining results also demonstrated an upregulation of Foxp3 and a downregulation of IL-17A expression in the liver tissues of mice with UBE2O overexpression (Figure 2*H*). Taken together, these findings demonstrated that UBE2O modulated the hepatic Treg/Th17 ratio to restore immune tolerance and alleviate AIH.

UBE2O Rewires Naïve CD4^{+}T Cell Differentiation Through Reducing IL-6 in Hepatocytes

Hepatocytes have been identified as innate immune cells with the capability to secrete substantial quantities of

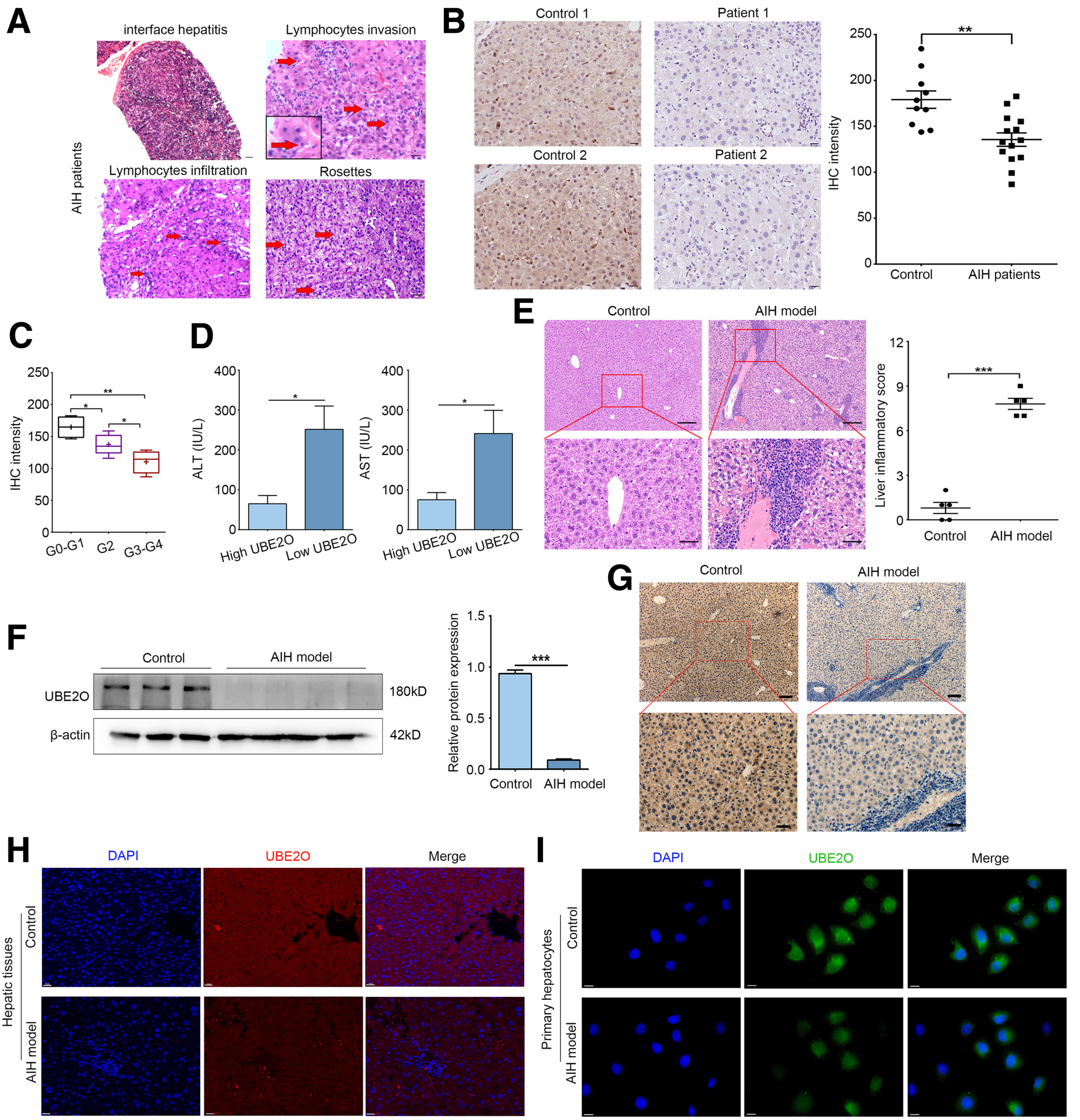

Figure 1. UBE2O is significantly downregulated in hepatic tissues of AIH. (*A*) Representative H&E staining of liver tissue sections from patients with AIH. *Red arrow* indicates the lymphocytes invasion, lymphocytes infiltration, and rosettes, respectively. Bars, 100 μm. (*B*) Representative IHC staining images showing UBE2O expression (*left*) and IHC intensity (*right*) in control and patients with AIH. Bars, 20 μm. *$P < .05$; **$P < .01$. Mean ± SEM. Student's *t*-test. (*C*) Comparison of UBE2O IHC intensity in patients with AIH from different inflammation grades. *$P < .05$; **$P < .01$. Mean ± SEM. One-way ANOVA. (*D*) Comparisons of serum ALT and AST levels in patients with AIH of relatively high or low UBE2O expression. *$P < .05$. Mean ± SEM. Student's *t*-test. (*E*) Representative H&E staining images showing the successful establishment of the chronic AIH model (*upper*) and liver inflammatory scores (*lower*). Bars, 200 μm (*upper panel*); 50 μm (*lower panel*). *$P < .05$; **$P < .01$; ***$P < .001$. Mean ± SEM. Student's *t*-test. (*F*) Western blotting analysis of hepatic UBE2O protein expression level in the control and mice with chronic AIH (*left*). Semi-quantification (*right*). *$P < .05$; **$P < .01$; ***$P < .001$. Mean ± SEM. Student's *t*-test. (*G*) Representative IHC staining images showing UBE2O expression in the control and mice with chronic AIH. Bars, 200 μm (*upper panel*); 50 μm (*lower panel*). (*H*) Representative IF staining images showing UBE2O expression in hepatic tissues from the control and mice with chronic AIH. Bars, 20 μm. (*I*) Representative IF staining images showing UBE2O expression in primary hepatocytes from the control and mice with chronic AIH. Bars, 20 μm.

cytokines. Therefore, we investigated the potential role of UBE2O in regulating key inflammatory factors within hepatocytes, which may influence the differentiation direction of T cells in the hepatic microenvironment. We isolated hepatocytes from chronic AIH mice and normal control mice respectively, and obtained culture supernatants enriched with hepatocyte-secreted cytokines. Naïve $CD4^+$ T cells were cultured with the supernatants from each group

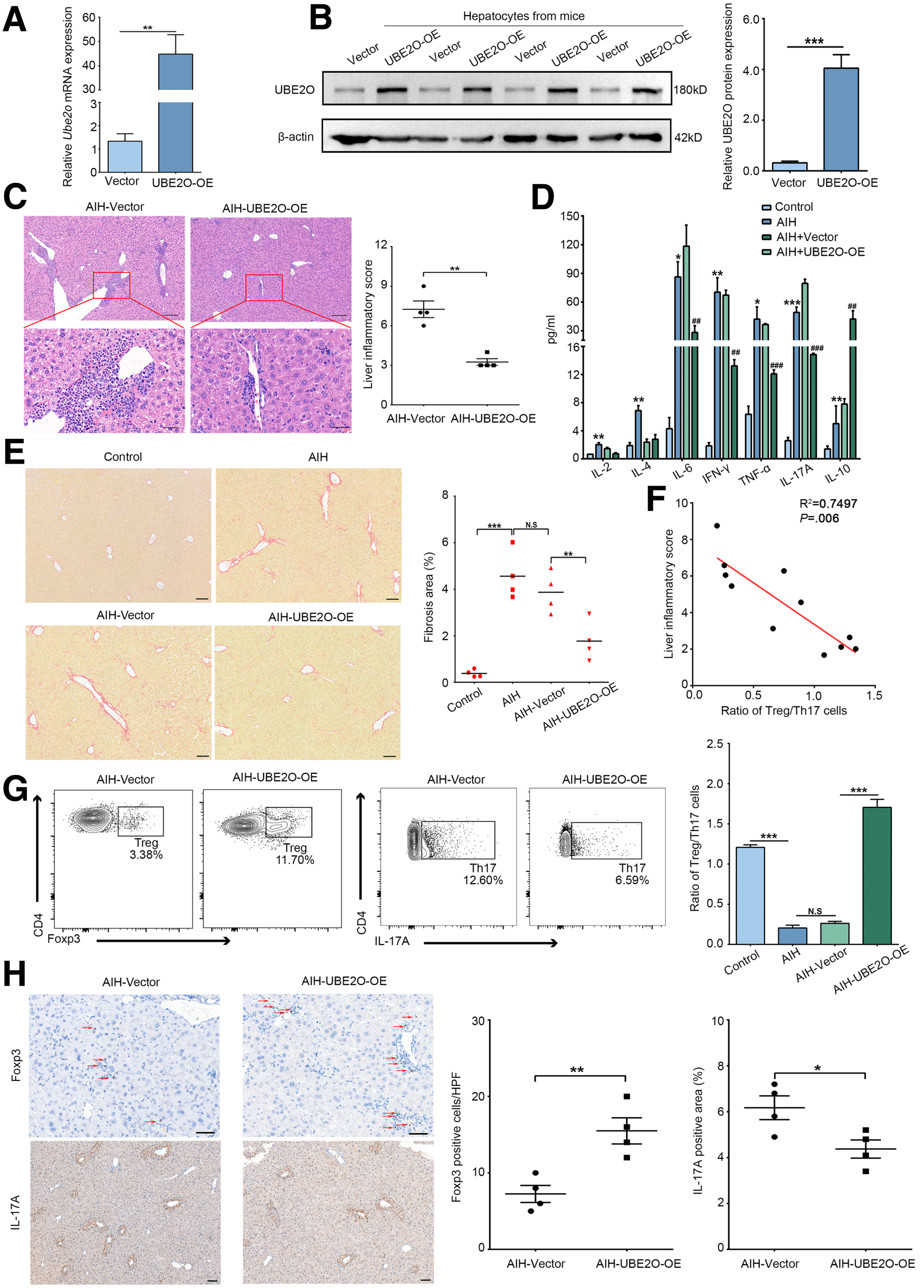

of hepatocytes, and then were collected for further analysis. The results showed that the proportion of Tregs was significantly reduced in those co-cultured with supernatants of hepatocytes in chronic AIH mice compared with the control group, whereas the proportion of Th17 cells was markedly increased (Figure 3*A*). These results suggested that, in chronic AIH, the altered cytokine secretion patterns of hepatocytes influenced the differentiation of hepatic $CD4^+$ T cell differentiation, disrupting the Treg/Th17 balance and immune tolerance, as evidenced by the decreased proportion of Tregs and increased proportion of Th17 cells. Based on the aforementioned findings, we focused on exploring the influence of UBE2O on hepatocytes. To ascertain whether UBE2O regulated the cytokine secretion patterns of hepatocytes in vitro, we successfully established AML12 hepatocytes with UBE2O overexpression (Figure 3*B*) and collected the culture medium from an lipopolysaccharide (LPS)-treated hepatocyte model for co-culture with naïve $CD4^+$T cells. Compared with those co-cultured with control, the proportions of Tregs were markedly downregulated, whereas Th17 cell proportions were increased in co-cultures with supernatants from LPS-treated AML12 hepatocytes. However, these alterations were partially reversed upon UBE2O overexpression (Figure 3*C* and *D*). These findings indicated that UBE2O could promote the differentiation of naïve $CD4^+$ T cells into Tregs by modulating hepatocyte-secreted cytokines, as well as inhibit or reduce their differentiation into Th17 cells.

To further investigate the hepatocyte-secreted cytokines patterns and identify the key cytokines regulated by UBE2O that influence the differentiation of naïve $CD4^+$T cells, we employed a proteome profiler cytokine solid-phase antibody microarray to analyze the cytokines present in the cell supernatants. Interestingly, among the cytokines that exhibited significant differences, it was observed that hepatocytes are capable of secreting substantial amounts of IL-6. Notably, this secretion was markedly reduced upon the overexpression of UBE2O (Figure 3*E*). The recruitment of T cells is reported to be involved in AIH pathogenesis[19]; meanwhile, we also evaluated the impact of UBE2O on chemokines involved in T-cell recruitment (eg, CCL5/CXCL9/CXCL10). However, the results showed that UBE2O seemed have no influence on the expression of these chemokines (Figure 4*A* and *B*). IL-6 plays a pivotal role in the regulation of naïve $CD4^+$ T cell differentiation. Specifically, IL-6 can inhibit the differentiation of naïve $CD4^+$T cells into Tregs while promoting their differentiation into Th17 cells, as well as facilitating the conversion of Tregs into Th17 cells.[20,21] We, therefore, postulated that UBE2O might restore the Treg/Th17 balance by reducing IL-6 levels in hepatocytes. To verify our hypothesis, we analyzed the messenger RNA (mRNA) and protein expression of IL-6 by quantitative real-time-polymerase chain reaction (qRT-PCR) and Western blotting assays in an LPS-treated hepatocytes model. Our findings indicated that UBE2O overexpression counteracted the LPS-induced increase in IL-6 compared with control cells (Figure 3*F* and *G*). Moreover, consistent results were detected in enzyme-linked immunosorbent assay (ELISA) measurement of IL-6, which further supported the microarray data (Figure 3*H*). To confirm the importance of hepatocyte-secreted IL-6 in regulating naïve $CD4^+$T differentiation, we isolated hepatocytes from chronic AIH and control mice, and introduced either an IL-6 antibody or an isotype control, followed by co-culturing the isolated naïve $CD4^+$T cells with the prepared culture medium. As presented in Figure 3*I*, the neutralization of IL-6 abrogated the inhibitory effect of supernatants from hepatocytes of chronic AIH mice on the differentiation of naïve $CD4^+$ T cells into Tregs. Altogether, our findings suggested that UBE2O might attenuate the expression and secretion of IL-6 to restore the differentiation of naïve $CD4^+$ T cells into Tregs and inhibit their differentiation into Th17 cells, thus contributing to the re-establishment of immune tolerance in AIH.

UBE2O Regulates IL-6 Expression Through YBX1 in Hepatocytes

Considering that UBE2O functions as an enzyme involved in post-translational modification, we conducted immunoprecipitation/mass spectrometry (IP/MS) analysis to identify potential interacting proteins and elucidate the underlying molecular mechanisms (Supplementary Table 1). Given previous studies and the results from IP/

Figure 2. (***See previous page***). **UBE2O overexpression ameliorates AIH and restores hepatic Treg/Th17 balance.** (*A*) qRT-PCR analysis of hepatic *Ube2o* mRNA expression level in mice injected with vector and UBE2O-OE. *$P < .05$; **$P < .01$. Mean ± SEM. Student's *t*-test. (*B*) Western blotting analysis of hepatocellular UBE2O protein expression level in mice injected with vector and UBE2O-OE plasmids. *$P < .05$; **$P < .01$; ***$P < .001$. Mean ± SEM. Student's *t*-test. (*C*) Representative H&E staining images of hepatic tissues of the chronic AIH mice without or with UBE2O overexpression (*left*) and liver inflammatory scores (*right*). Bars, 200 μm (*upper panel*); 50 μm (*lower panel*). *$P < .05$; **$P < .01$. Mean ± SEM. Student's *t*-test. (*D*) Several cytokines in the serum of mice from control, AIH, AIH + vector, and AIH + UBE2O-OE group were detected using a CBA kit. $^{\#}P < .05$; $^{\#\#}P < .01$; $^{\#\#\#}P < .001$ (vs the control group). Mean ± SEM. One-way ANOVA. *$P < .05$; **$P < .01$; ***$P < .001$ (vs the chronic AIH group). Mean ± SEM. One-way ANOVA. (*E*) Representative Sirius red staining of liver tissues in the indicated groups (*left*) and liver fibrosis scores. Bars, 100 μm (*left*). *$P < .05$; **$P < .01$; ***$P < .001$; N.S, no significance. Mean ± SEM. One-way ANOVA. (*F*) Correlation analysis of Treg/Th17 cell ratios and liver inflammatory scores in the livers of mice with control or chronic AIH (Spearman's coefficient $R^2 = 0.07497$; $P = .006$). (*G*) The proportion of intrahepatic Tregs and Th17 cells (*left*). Statistical graph of the Treg/Th17 cell ratio in the livers from the indicated groups of chronic AIH model (*right*). *$P < .05$; **$P < .01$; ***$P < .001$; N.S, no significance. Mean ± SEM. One-way ANOVA. (*H*) Representative IHC staining images showing Foxp3 and IL-17A expression in hepatic tissues of chronic AIH mice injected with vector and UBE2O-OE plasmids. *Red arrows* indicate $Foxp3^+$cells. Bars, 50 μm (*upper panel*); 100 μm (*lower panel*) (*left*). Quantification of the IHC data of Foxp3 and IL-17A expression (*right*). *$P < .05$; **$P < .01$. Mean ± SEM. Student's *t*-test.

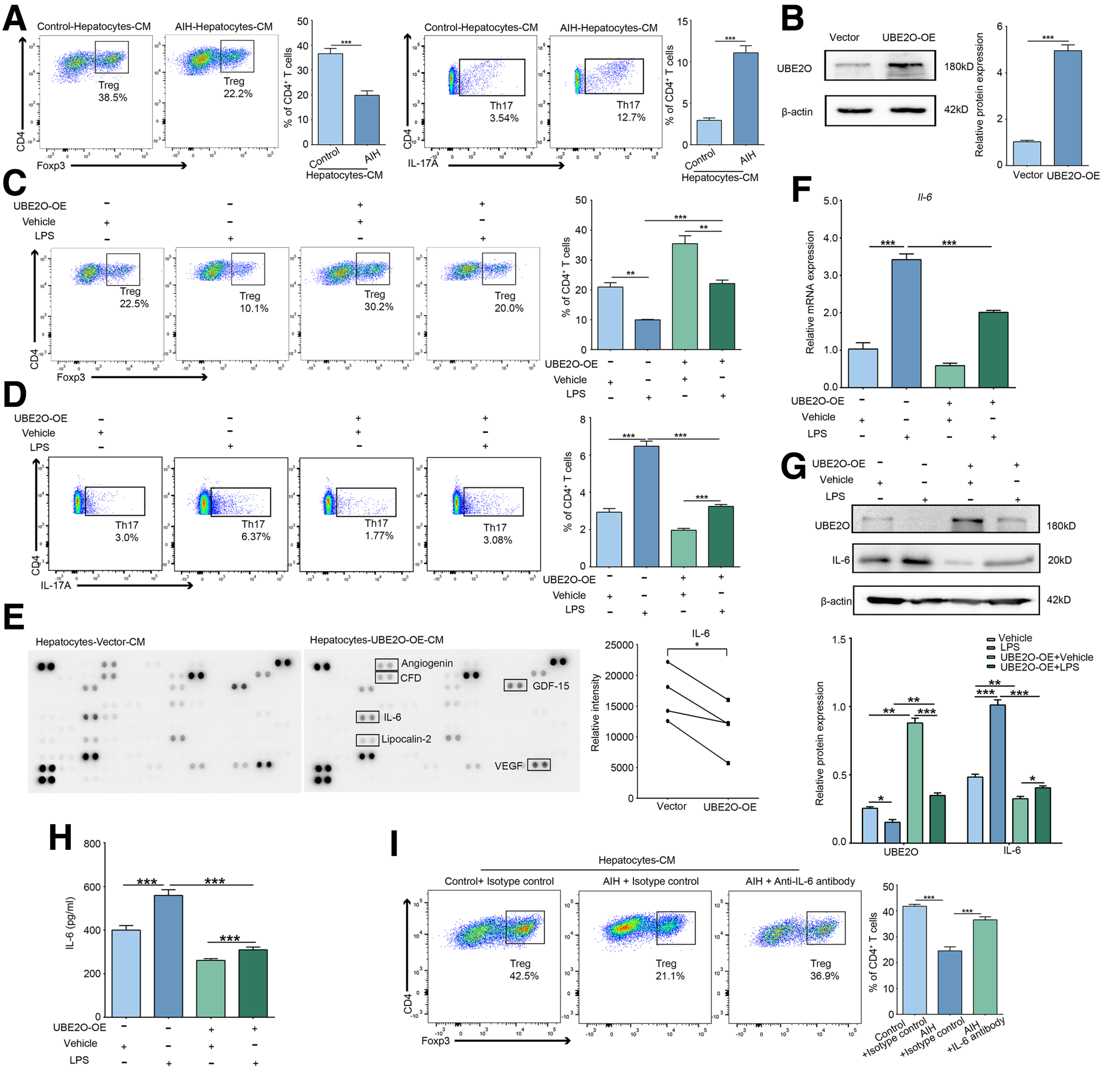

Figure 3. UBE2O rewires naïve CD4+T cell differentiation through reducing IL-6 in hepatocytes. (*A*) Induced Treg and Th17 cell differentiation assessed with culture supernatants of hepatocytes from control and chronic AIH mice. **P* < .05; ***P* < .01; ****P* < .001. Mean ± SEM. Student's *t*-test. (*B*) Western blotting analysis of UBE2O protein expression level in AML12 hepatocytes transfected with vector and UBE2O-OE plasmids (*upper panel*). Semi-quantification (*lower panel*). **P* < .05; ***P* < .01; ****P* < .001. Mean ± SEM. Student's *t*-test. (*C*) Induced Treg differentiation assessed with culture supernatants of AML12 hepatocytes from the experimental groups. **P* < .05; ***P* < .01; ****P* < .001. Mean ± SEM. One-way ANOVA. (*D*) Induced Th17 cell differentiation assessed with culture supernatants of AML12 hepatocytes from the experimental groups. **P* < .05; ***P* < .01; ****P* < .001. Mean ± SEM. One-way ANOVA. (*E*) Alteration of cytokines in the supernatants of control and UBE2O-overexpressing hepatocytes detected by solid-phase microarray of cytokines (*left*). Relative intensity of IL-6 (*right*). **P* < .05. Mean ± SEM. Student's *t*-test. (*F*) RT-qPCR analysis of *Il-6* mRNA expression level of AML12 hepatocytes from the experimental groups. **P* < .05; ***P* < .01; ****P* < .001. Mean ± SEM. One-way ANOVA. (*G*) Western blotting analysis of IL-6 and UBE2O protein expression level of AML12 hepatocytes from the experimental groups (*upper*). Semi-quantification (*lower*). **P* < .05; ***P* < .01; ****P* < .001. Mean ± SEM. One-way ANOVA. (*H*) ELISA analysis of IL-6 of the culture supernatants of AML12 hepatocytes from the experimental groups. **P* < .05; ***P* < .01; ****P* < .001. Mean ± SEM. One-way ANOVA. (*I*) Induced Treg differentiation assessed with culture supernatants of hepatocytes from control and chronic AIH mice treatment with isotype control or anti IL-6 antibody. **P* < .05; ***P* < .01; ****P* < .001. Mean ± SEM. One-way ANOVA.

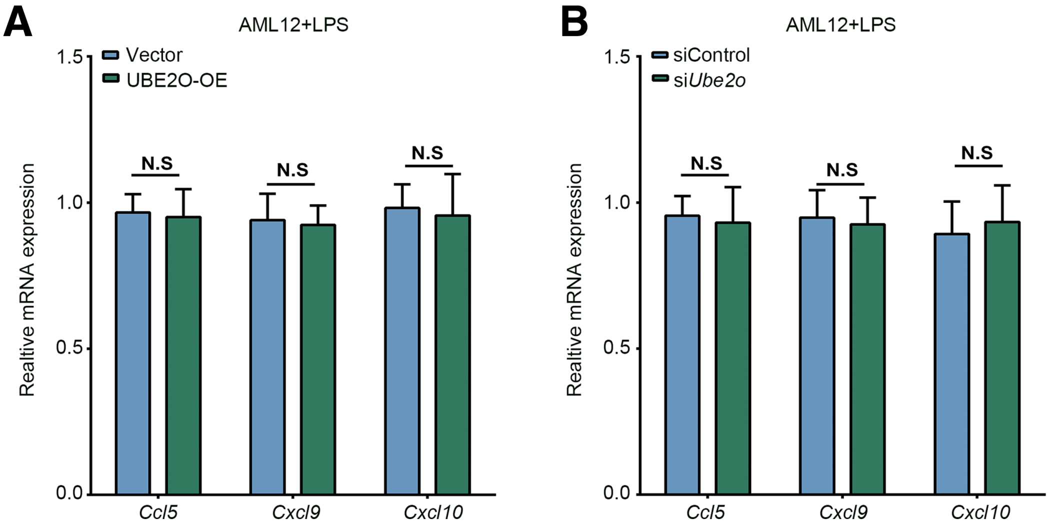

Figure 4. The effect of UBE2O on CCL5/CXCL9/CXCL10 expression. (*A*) qRT-PCR analysis of *Ccl5*, *Cxcl9*, and *Cxcl10* mRNA expression level in AML12 cells transfected with vector and UBE2O-OE plasmids. N.S, no significance. Student's *t*-test. (*B*) qRT-PCR analysis of *Ccl5*, *Cxcl9*, *Cxcl10* mRNA expression level in AML12 cells transfected with siControl and si*Ube2o*. N.S, no significance. Student's *t*-test.

MS analyses, we observed a potential interaction between UBE2O and YBX1, a regulator of various cytokines, including IL-6 (Figure 5*A*).[22,23] As YBX1 acts as a transcriptional factor for many genes, we retrieved the YBX1 binding motif from the JASPAR database (http://jaspardev.genereg.net/) (Figure 6*A*). We then screened the *Il-6* promoter region for potential *Ybx1* binding sites using JASPAR and selected the most probable site for mutation (Figure 6*B*). The dual-luciferase reporter assay suggested that *Ybx1* bound to the *Il-6* promoter, and the mutation abolished the promoter inducibility mediated by YBX1 overexpression (Figure 6*C*). Then, the interaction between UBE2O and YBX1 was further corroborated by co-immunoprecipitation (Co-IP) assays (Figure 5*B* and *C*). Furthermore, we also detected the decreased protein expression level of YBX1 with UBE2O overexpression in AML12 hepatocytes in a dose-dependent manner, along with the downregulation of IL-6 (Figure 6*D*). Western blotting results demonstrated that YBX1 overexpression could counteract the UBE2O-induced reduction in IL-6 expression in LPS-treated AML12 hepatocytes (Figure 6*E*). Consistent results were also observed in the IL-6 promoter luciferase activity assay and the secretion levels of IL-6 measured by the ELISA assay (Figure 5*F* and *G*). Prior studies have demonstrated that the nuclear translocation of YBX1 is important for the transcriptional activation of target genes.[24,25] As anticipated, IF staining results showed that UBE2O overexpression significantly reduced the YBX1 nuclear distribution (Figure 5*H*). These data delineated that UBE2O downregulated IL-6 expression through YBX1 in hepatocytes.

Because macrophages are another source of IL-6, to evaluate the contribution of macrophages to AIH pathogenesis, we depleted macrophages in AIH mice by using clodronate liposomes. The results of H&E staining showed that hepatic inflammatory infiltration and tissue damage remained evident in AIH mice after macrophage depletion, with no significant difference compared with untreated AIH controls (Figure 7*A*), indicating that macrophages may contribute only a limited role in AIH. The depletion experiment results suggested that removal of macrophages did not markedly ameliorate AIH pathology, implying that some other cell types might be more critical in this context of AIH. Moreover, the expression of IL-6 was higher in hepatocytes than nonparenchymal cells in AIH (Figure 7*B*). Then, because we wondered whether UBE2O/YBX1/IL-6 axis was also established in macrophages, we further analyzed the expression of UBE2O between hepatocytes and nonparenchymal cells in AIH. The results showed that UBE2O expression was abundant in hepatocytes (Figure 7*C*). In addition, we preliminarily examined the IL-6 levels in the supernatant of RAW264.7 cells, and found that UBE2O did not affect IL-6 levels in RAW264.7 cells (Figure 7*D*). Similarly, the results of Western blotting also indicated that UBE2O had no effects on YBX1 and IL-6 protein expression in RAW264.7 cells (Figure 7*E*). These results collectively demonstrated that macrophages played a minor role in AIH pathogenesis in our model and that the UBE2O/YBX1/IL-6 axis was primarily active in hepatocytes rather than in macrophages, which further prompted us to focus on IL-6 derived from hepatocytes in AIH.

UBE2O Promotes Ubiquitination Degradation of YBX1

As UBE2O has been identified as a ubiquitin-editing enzyme, we wondered whether UBE2O promoted ubiquitination degradation of YBX1. Therefore, we used MG132, a proteasome inhibitor, to elucidate the degradation pathway of YBX1. The results showed that MG132 treatment led to a significant accumulation of endogenous YBX1

protein in AML12 hepatocytes (Figure 8A). In addition, the reduction in YBX1 protein levels was markedly reversed under MG132 treatment with UBE2O overexpression. Conversely, in AML12 hepatocytes with UBE2O down-regulation, MG132 treatment exacerbated the increase in YBX1 protein levels, indicating that YBX1 was presumably

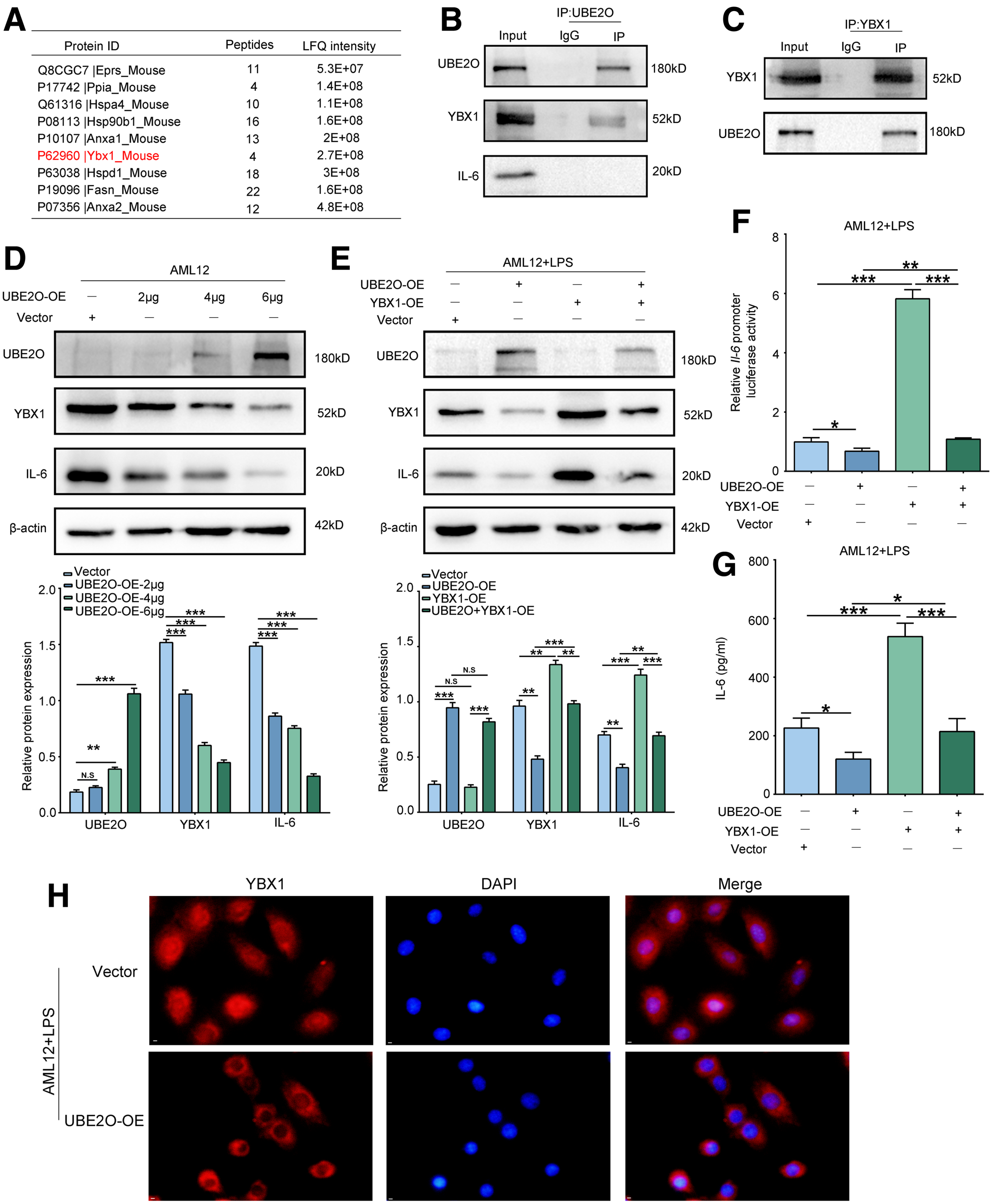

degraded via the proteasome pathway (Figure 8*B* and *C*). As illustrated in Figure 8*D* and *E*, the half-life of YBX1 was considerably shortened after UBE2O overexpression, whereas the half-life of YBX1 protein was substantially prolonged in AML12 hepatocytes with UBE2O knockdown. Because lysine 48 (K48)-linked ubiquitination (K48-Ub) is a canonical form of protein ubiquitination, we performed Co-IP assays to detect the K48-Ub level of YBX1. The results demonstrated a significant increase in the K48-Ub level of YBX1 protein following UBE2O overexpression, whereas UBE2O knockdown resulted in a decrease in the level of K48-Ub of YBX1 protein (Figure 8*F*).

We then predicted the 4 most potential residues (K62, K79, K90, K135) by combining amino acid sequence analysis of YBX1 across different species and GPS-Uber analysis (http://gpsuber.biocuckoo.cn/) (Figure 8*G*). Accordingly, 4 YBX1 mutants were constructed for analysis, namely K62R, K79R, K90R, and K135R (lysine-to-arginine). The results of Co-IP assays revealed that the K135R mutation disrupted the interaction between YBX1 and UBE2O, as well as the ubiquitination level of the YBX1 protein. Conversely, the other mutants of YBX1 did not affect the interaction between UBE2O and YBX1 or the ubiquitination of YBX1 (Figure 8*H*). Sequence alignment also indicated the high conservation of this lysine residue across species (Figure 8*I*), implying post-translational modification of this residue might hold biological significance. Collectively, our data suggested that UBE2O promoted ubiquitination degradation of YBX1 at K135.

YBX1 Is Crucial for the Protective Effects Mediated by UBE2O in AIH

The morbidity of AIH is much higher in women than men. To substantiate our hypothesis that YBX1 is implicated in the regulation of IL-6 by UBE2O in vivo, we upregulated YBX1 in mice with UBE2O overexpression in chronic AIH. In comparison to the AIH control group, mice with UBE2O overexpression exhibited a significant reduction in inflammatory cell infiltration, whereas this infiltration was exacerbated in mice with YBX1 overexpression (Figure 9*A*). As expected, YBX1 was found to partially negate the protective effects of UBE2O against AIH, as evidenced by histological analyses using H&E and Sirius red staining (Figure 9*A* and *B*). Additionally, it was observed that the UBE2O-mediated reduction in IL-6 protein expression in hepatocytes was reversed in the context of YBX1 overexpression, which was consistent with in vitro experiments (Figure 9*C*). Furthermore, the IHC staining results showed a reduction in forkhead box p3 (Foxp3) and an increase in IL-17A expression in liver tissues in mice of both UBE2O and YBX1 overexpression, compared with those with UBE2O overexpression alone (Figure 9*D*). Together, these results further demonstrated that UBE2O protected against AIH through modulation of YBX1.

Comparison of UBE2O/YBX1/IL-6 Expression and Tregs Between Sexes in AIH

AIH is recognized as a consequence of dysregulated immune activation, with a notably higher prevalence in females than in males. To investigate whether gender influences the UBE2O/YBX1/IL-6 axis and Tregs in AIH, we preliminarily analyzed the expression of UBE2O, YBX1, Foxp3, and serum IL-6 levels between sexes in both patients and the mouse model. The IHC and ELISA results showed that there were no statistically significant differences in expression of UBE2O, YBX1, Foxp3, and serum IL-6 levels between males and females (Figure 10*A*–*D*). These data indicated that, under our experimental conditions, gender exerted minimal influence on UBE2O/YBX1/IL-6 axis and Tregs in AIH.

Discussion

AIH represents a significant global health challenge, with its incidence on the rise and garnering increasing attention. To date, no effective treatment has been established, posing a serious threat to human health.[2] Although immune tolerance is increasingly recognized as a critical factor in the progression of AIH, the underlying mechanisms still remain unelucidated.[1] In addition to traditional immunosuppressants, in recent years, certain interventions employed in other autoimmune disease treatments are being tried for potential application in AIH treatment. These include the administration of anti-TNF-α antibodies, recombinant IL-10, cytotoxic T-lymphocyte-associated protein 4 (CTLA4) Ig, and the adoptive transfer of Tregs, although their efficacy has been limited.[6,26–28] The clinical management of AIH thus continues to present significant

Figure 5. (***See previous page***). **UBE2O regulates IL-6 expression through YBX1 in hepatocytes.** (*A*) IP/MS detection of the potential proteins that interact with UBE2O. (*B*) UBE2O was immunoprecipitated from AML12 hepatocytes and subjected to Western blotting assays to detect its interaction with YBX1 and IL-6. IgG was used as a negative control. (*C*) YBX1 was immunoprecipitated from AML12 hepatocytes and subjected to Western blotting assays to detect its interaction with UBE2O. IgG was used as a negative control. (*D*) Western blotting analysis of UBE2O, YBX1, and IL-6 protein levels in AML12 hepatocytes transfected different amounts of UBE2O-expressing plasmids (*upper panel*). Semi-quantification (*lower panel*). **P* < .05; ***P* < .01; ****P* < .001,; N.S, no significance. Mean ± SEM. One-way ANOVA. (*E*) Western blotting analysis of UBE2O, YBX1, and IL-6 protein levels in control and UBE2O-overexpressing AML12 hepatocytes with LPS treatment, treated without or with YBX1 overexpression (*upper panel*). Semi-quantification (*lower panel*). **P* < .05; ***P* < .01; ****P* < .001; N.S, no significance. Mean ± SEM. One-way ANOVA. (*F*) Luciferase reporter assays showing the luciferase activities of *Il-6* promoter in control and UBE2O-overexpressing AML12 hepatocytes with LPS treatment, treated without or with YBX1 overexpression. **P* < .05; ***P* < .01; ****P* < .001. Mean ± SEM. One-way ANOVA. (*G*) ELISA analysis of IL-6 of the culture supernatants in control and UBE2O-overexpressing AML12 hepatocytes with LPS treatment, treated without or with YBX1 overexpression. **P* < .05; ***P* < .01; ****P* < .001. Mean ± SEM. One-way ANOVA. (*H*) The cellular localization of the YBX1 protein in AML12 hepatocytes following UBE2O overexpression detected by IF staining assays. Bars, 500 μm.

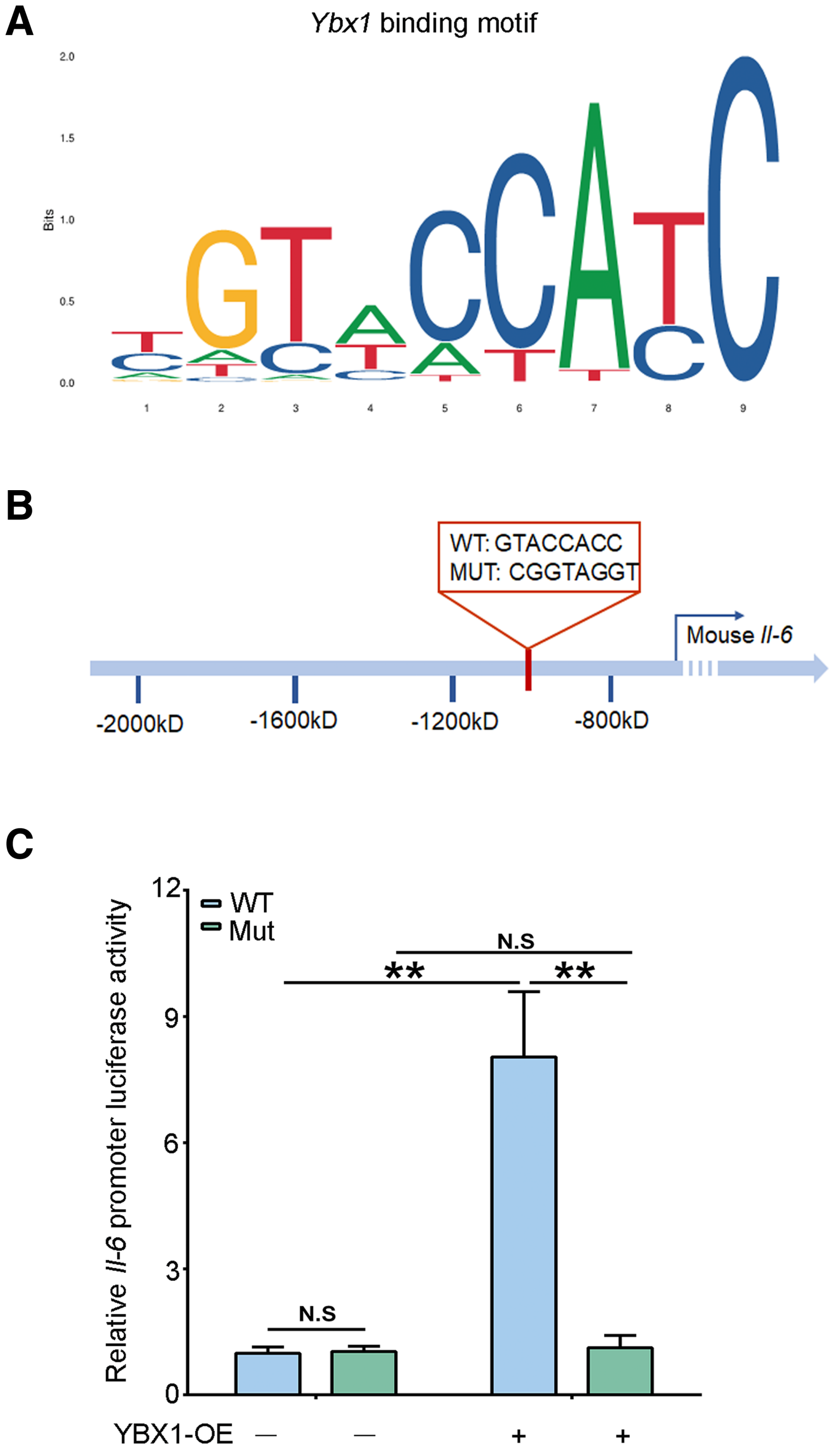

Figure 6. YBX1 promotes *Il-6* transcription. (*A*) The *Ybx1* binding motif predicted in JASPAR database. (*B*) Mutation of most potential binding site of *Ybx1* in *Il-6* promoter sequence. MUT, mutant. (*C*) Luciferase reporter assays showing the luciferase activities of WT or mutants in control and AML12 hepatocytes with YBX1 overexpression. *$P < .05$; **$P < .01$; N.S, no significance. Mean ± SEM. One-way ANOVA.

challenges. Therefore, exploring novel immunomodulation-related targets may offer valuable clues for AIH treatment. In the present study, we specifically identified the ubiquitin-conjugating enzyme UBE2O as a hepatic protector against AIH, primarily through its regulation of cytokines secreted by hepatocytes, thus restoring Treg/Th17 immune homeostasis. Mechanistically, we confirmed that UBE2O interacted with and facilitated ubiquitination degradation of YBX1 at K135 in hepatocytes, thereby reducing IL-6 expression and ultimately restoring the balance between Tregs and Th17 cells. However, this protective function of UBE2O was compromised in patients and mice with AIH due to its downregulation. Targeting UBE2O might provide a promising strategy for AIH therapy.

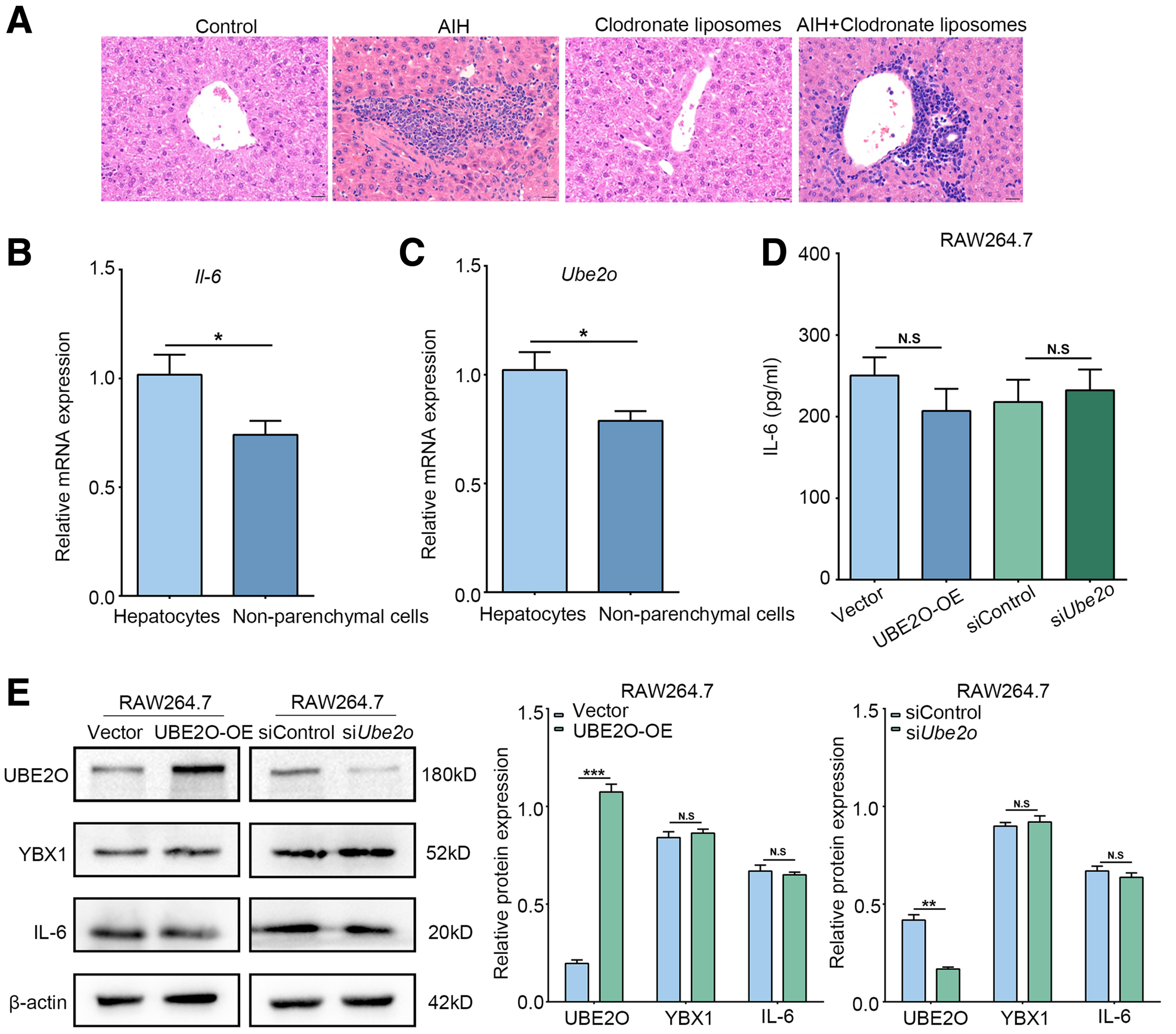

Figure 7. Macrophages contribute a limited role in AIH. (*A*) Representative H&E staining images of hepatic tissues of mice from control, AIH, clodronate liposomes, and AIH+ clodronate liposomes. Bars, 50 μm. (*B*) qRT-PCR analysis of *Il-6* mRNA expression level in hepatocytes and nonparenchymal cells from chronic AIH model. **P* $<$ .05. Mean $\pm$ SEM. Student's *t*-test. (*C*) qRT-PCR analysis of *Ube2o* mRNA expression level in hepatocytes and nonparenchymal cells from chronic AIH model. **P* $<$ .05. Mean $\pm$ SEM. Student's *t*-test. (*D*) ELISA analysis of IL-6 of the culture supernatants of RAW267.4 cells from the indicated groups. N.S, no significance. Mean $\pm$ SEM. One-way ANOVA. (*E*) Western blotting analysis of UBE2O, YBX1, and IL-6 protein levels in RAW264.7 cells from the experimental groups (*left*). Semi-quantification (*right*). **P* $<$ .05; ***P* $<$ .01; ****P* $<$ .001; N.S, no significance. Mean $\pm$ SEM. One-way ANOVA.

In experimental AIH, the overexpression of UBE2O was identified to mitigate the aggravation of hepatic inflammation and fibrosis. We also observed that UBE2O overexpression resulted in an increase in IL-10 and a decrease in IL-17A levels in mice with AIH. IL-10, primarily secreted by Tregs, is a crucial immunosuppressive cytokine, whereas IL-17A, predominantly secreted by Th17 cells, is a key pathogenic factor in AIH. Tregs and Th17 cells are critical subtypes of $CD4^+$ effector T cells in the liver microenvironment.[8] The impaired balance of Treg/Th17 is implicated in the pathogenesis and progression of AIH.[9,29] Our previous study has detected that tofacitinib treatment could alleviate concanavalin A (conA)-induced liver injury by increasing the Treg/Th17 ratio.[11] In this study, we found that hepatic overexpression of UBE2O restored Treg/Th17 balance in the liver microenvironment, evidenced by an increased proportion of Tregs and a decreased proportion of Th17 cells. Consequently, our study suggested that the protective effect of UBE2O on AIH can be attributed to its role in modulating intrahepatic immune disorders.

Hepatocytes, a predominant parenchymal cell type in the liver, possess a significant capacity to secrete cytokines,

thereby exacerbating inflammatory injury within the organ. A growing body of evidence highlights the interaction between hepatocytes and immune cells during liver inflammation.[30] Although numerous studies have concentrated on the immune microenvironment's attack on hepatocytes, the role of hepatocytes as innate immune cells in cytokine secretion and their impact on immune cells, particularly T cell differentiation, warrants further investigation. Here, our results indicated that overexpression of UBE2O in hepatocytes altered the cytokine secretion patterns, facilitating the differentiation of naïve CD4^{+} T cells into Tregs. Importantly, we noticed that among the cytokines exhibiting significant differences, IL-6, which could be secreted by hepatocytes, was markedly reduced with UBE2O

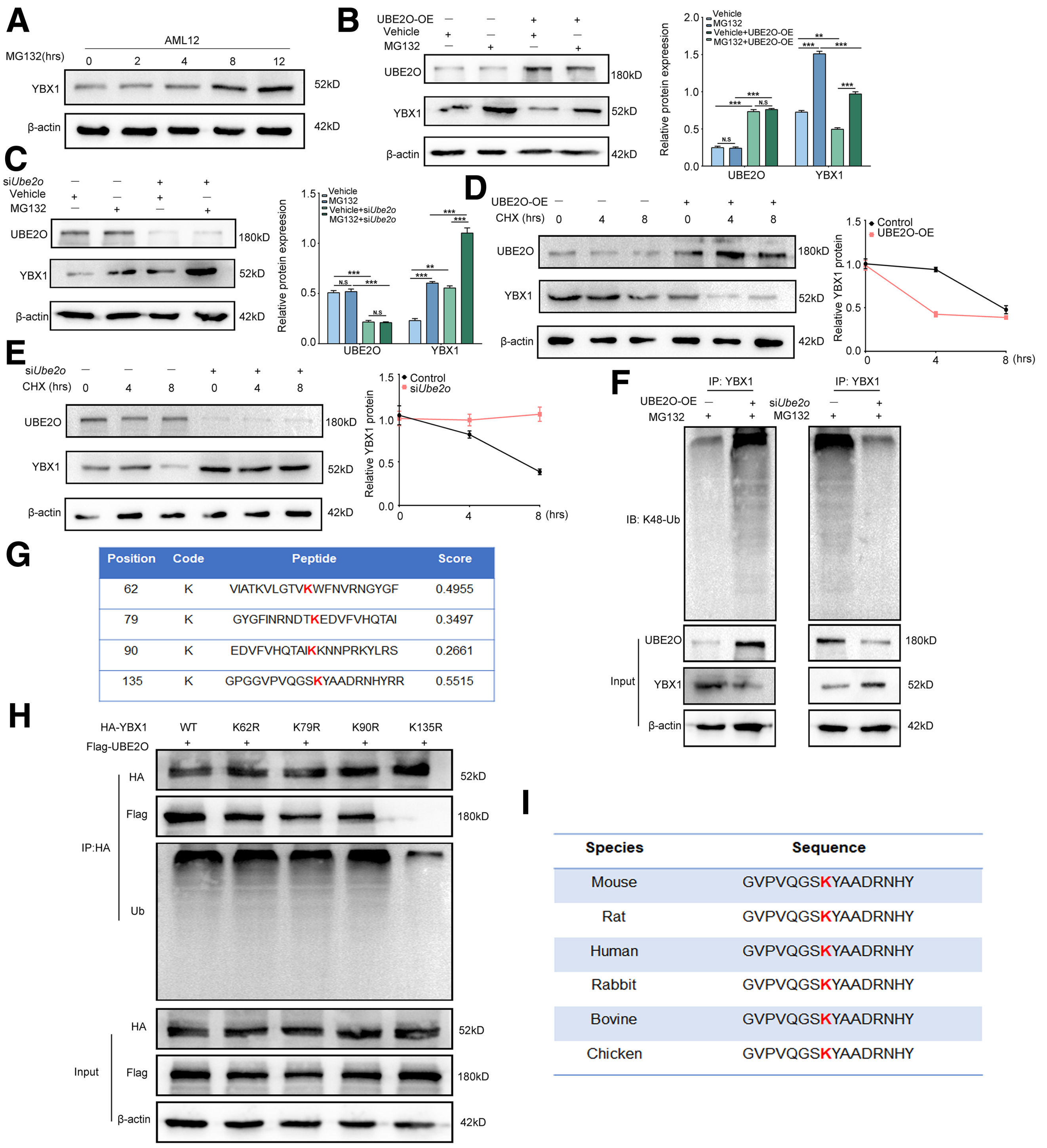

overexpression. IL-6 has been considered to play a key role in sustaining the differentiation balance between Tregs and Th17 cells.[31,32] Previously, we reported that liver injury induced by viral or chemical agents led to a sustained increase in IL-6 levels, which suppresses Foxp3 gene expression and consequently inhibited the differentiation of $CD4^+CD25^+$ cells into suppressive $Foxp3^+$ Tregs in the liver rather than in the spleen.[33] Our in vitro experiments revealed that neutralizing IL-6 with specific antibodies counteracted the inhibitory effects of supernatants from hepatocytes of AIH mice on the differentiation of naïve $CD4^+$ T cells into Tregs. Thus, UBE2O appeared to restore hepatic Treg/Th17 balance by modulating cytokines secreted from hepatocytes, which functioned as innate immune cells, with IL-6 potentially playing a significant role in this process.

IL-6 exhibits different functions across different types of hepatitis. In viral hepatitis, IL-6 is elevated acutely, where it promotes both inflammation and hepatic regeneration. However, during chronic infection, it sustains immune activation, thereby favoring fibrosis and HCC.[34] In alcoholic liver disease, IL-6 has dual effects as proinflammatory in early injury and regenerative in later stages, and IL-6/gp130 activation alleviates steatohepatitis in murine models.[35] In nonalcoholic fatty liver disease (NAFLD), IL-6 is correlated with obesity and insulin resistance, and may either exacerbate or ameliorate steatosis depending on different contexts.[34,36] In acute-on-chronic liver failure (ACLF), an early rise in IL-6 correlates with poor prognosis, yet IL-6 also supports regeneration following injury, as seen in acetaminophen-induced hepatotoxicity.[37,38] The role of IL-6 is dual and stage-specific: protective in regeneration, yet pathogenic in chronic inflammation. The dominant effect is determined by the underlying etiology of hepatitis and the timing of IL-6 signaling, as well as genetic or environmental modifiers. In this study, IL-6 derived from hepatocytes, rather than from macrophages, appears to be a major driver in the chronic AIH model, consistent with the regenerative/proinflammatory balance characteristic of AIH.

When exploring the exact mechanisms underlying the regulation of IL-6 by UBE2O, we performed an IP/MS analysis and focused on YBX1, which could act as a transcription regulator of multiple inflammatory factors. As UBE2O is a ubiquitin-binding enzyme, and ubiquitin always serves as a signal for protein degradation,[39] we speculated that UBE2O might exert its effects through its role in the regulation of ubiquitination modification. In the present study, we found that YBX1 could enhance the transcriptional activation of IL-6, and confirmed its involvement in the regulation of IL-6 expression and production by UBE2O in hepatocytes. In addition, our findings also revealed that the observed increase in IL-6 production in hepatocytes with UBE2O overexpression might be attributed to the ubiquitination degradation of YBX1 protein mediated by UBE2O. In previous studies, YBX1 has been identified as undergoing various post-translational modifications at distinct residues. For example, serine 176 (S176) and serine 165 (S165) phosphorylation of YBX1 is critical for NF-κB activation, with mutations at these serine residues influencing the regulation of NF-κB target genes.[40] Another study has shown that K48-Ub modification of YBX1 is prevented by activated Protein C (aPC) depending on Otubain1 (OTUB1) in renal ischemia-reperfusion injury.[41] A recent study also indicates that YBX1 is O-GlcNAcylated at threonine 57 (T57) in HCC, which stabilizes the protein and increases its expression.[42] In this study, we first reported that YBX1 could be ubiquitinated at K135, as mutation at this residue abolished the interaction between UBE2O and YBX1, as well as ubiquitination modification of YBX1 protein. However, further investigations are needed to identify the role of this residue in AIH.

When analyzing the UBE2O/YBX1/IL-6 axis and Tregs in patients with AIH and the mouse model, we found no statistically significant differences between male and female groups in either cohort, indicating that, under our experimental conditions, gender may not be a major factor influencing the UBE2O/YBX1/IL-6 axis or T-cell subset distribution in AIH. Possible reasons include the relatively limited sample size, the incomplete recapitulation of human hormonal environments in the mouse model, or the presence of other overriding factors such as genetic background and environmental triggers. Although no gender disparity was observed, this result is important to inform future larger-scale or hormone related studies.

These findings raise several new questions that warrant further investigation. For instance, more explorations are

Figure 8. (***See previous page***). **UBE2O promotes ubiquitination degradation of YBX1.** (*A*) Western blotting analysis of YBX1 protein level in AML12 hepatocytes treated with proteasome inhibitor MG132 (20 μM) for 0, 2, 4, 8, and 12 hours. (*B*) AML12 hepatocytes transfected with UBE2O-OE plasmids or vector treated with or without MG132. Western blotting analysis of UBE2O and YBX1 protein levels for each group (*left*). Semi-quantification (*right*). *$P < .05$; **$P < .01$; ***$P < .001$; N.S, no significance. Mean $\pm$ SEM. One-way ANOVA. (*C*) AML12 hepatocytes transfected with si*Ube2o* or control treated with or without MG132. Western blotting analysis of UBE2O and YBX1 protein levels for each group (*left*). Semi-quantification (*right*). *$P < .05$; **$P < .01$; ***$P < .001$; N.S, no significance. Mean $\pm$ SEM. One-way ANOVA. (*D*) AML12 hepatocytes transfected with UBE2O-OE plasmids or vector, and then treated with 10 μM CHX for 0, 4, and 8 hours. Western blotting analysis of UBE2O and YBX1 protein levels for each group (*left*). Semi-quantification (*right*). (*E*) AML12 cells transfected with si*Ube2o* or control, and then treated with 10 μM CHX for 0, 4, and 8 hours. Western blotting analysis of UBE2O and YBX1 protein levels for each group (*left*). Semi-quantification (*right*). (*F*) Endogenous YBX1 K48-ubiquitination in AML12 hepatocytes transfected with UBE2O-OE plasmids or vector, si*Ube2o* or control. Lysates from cells were immunoprecipitated with YBX1 antibody before subjected to immunoblotting with Ub-K48 antibody. (*G*) Potential ubiquitination sites for mouse YBX1 predicted by GPS-Uber. (*H*) HA-tagged WT, K62R, K79R, K90R, and K135R constructs of YBX1 were respectively expressed in AML12 hepatocytes with Flag-UBE2O plasmid transfection. Cell lysates containing HA-tagged YBX1 were immunoprecipitated using an anti-HA antibody, and immunoblot assay were performed to detect Flag-UBE2O or YBX1 ubiquitination level. (*I*) The amino acid sequence alignment around K135 of YBX1 protein among different species.

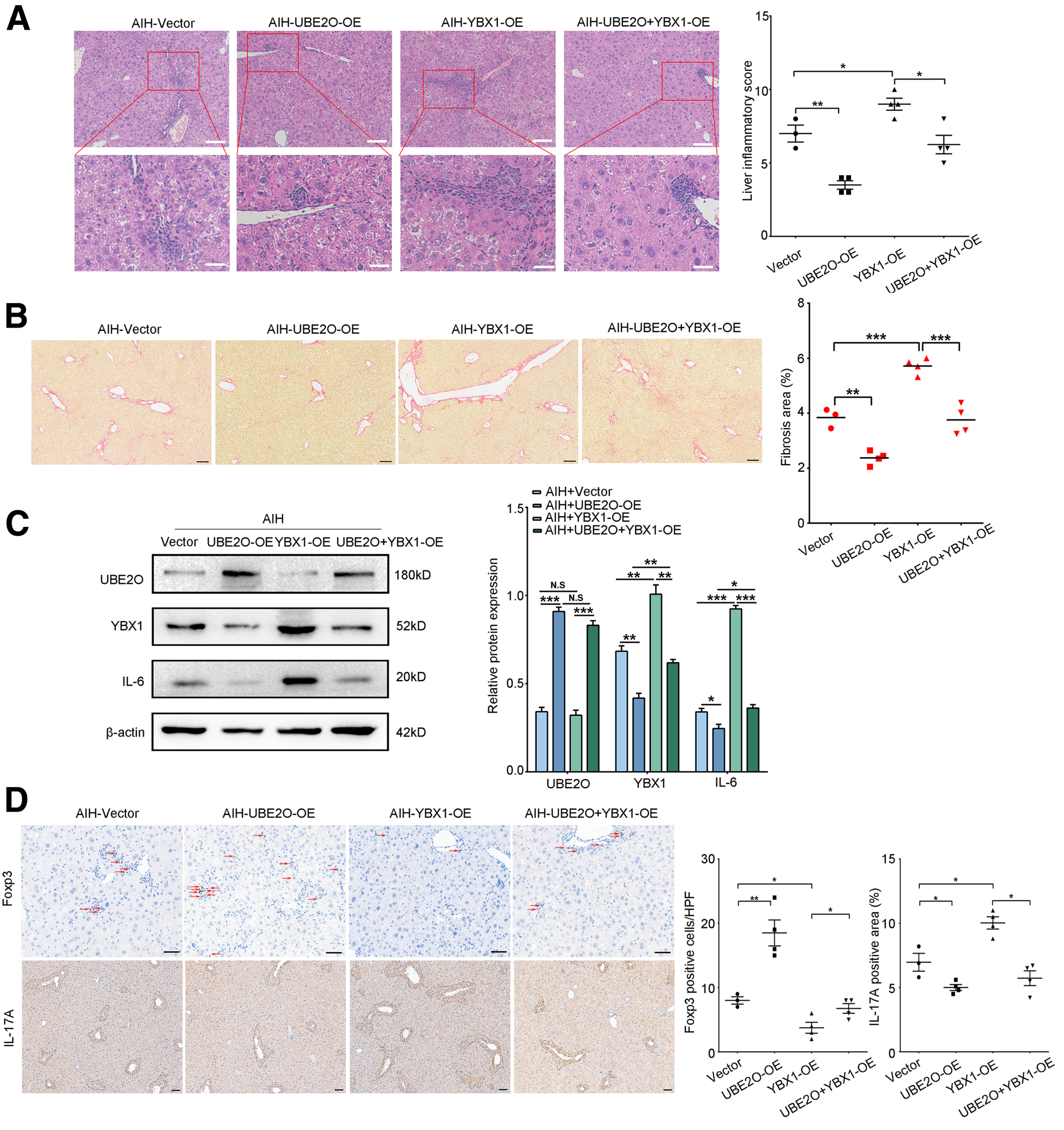

Figure 9. YBX1 is crucial for the protective effects mediated by UBE2O in AIH. (*A*) Representative H&E staining images of hepatic tissues of chronic AIH mice injected with vector, UBE2O-OE, YBX1-OE, and UBE2O+YBX1-OE plasmids (*left*); liver inflammatory scores (*right*). Bars, 200 μm (*upper panel*); 50 μm (*lower panel*). *P < .05; **P < .01; Mean ± SEM. One-way ANOVA. (*B*) Representative Sirius red staining of liver tissues of chronic AIH mice injected with vector, UBE2O-OE, YBX1-OE, and UBE2O+YBX1-OE plasmids. Bars, 100 μm (*left*). Liver fibrosis score (*right*). *P < .05; **P < .01; ***P < .001; N.S, no significance. Mean ± SEM. One-way ANOVA. (*C*) Western blotting analysis of hepatic UBE2O, YBX1, and IL-6 protein levels of chronic AIH mice injected with vector, UBE2O-OE, YBX1-OE, and UBE2O+YBX1-OE plasmids (*left*). Semi-quantification (*right*). *P < .05; **P < .01; ***P < .001; N.S, no significance. Mean ± SEM. One-way ANOVA. (*D*) Representative IHC staining images showing Foxp3 and IL-17A expression in hepatic tissues of chronic AIH mice injected with vector, UBE2O-OE, YBX1-OE, and UBE2O+YBX1-OE plasmids. *Red arrows* indicate Foxp3^{+}cells. Bars, 50 μm (*upper panel*); 100 μm (*lower panel*) (*left*). Quantification of the IHC data of Foxp3 and IL-17A expression (*right*). *P < .05; **P < .01. Mean ± SEM. One-way ANOVA.

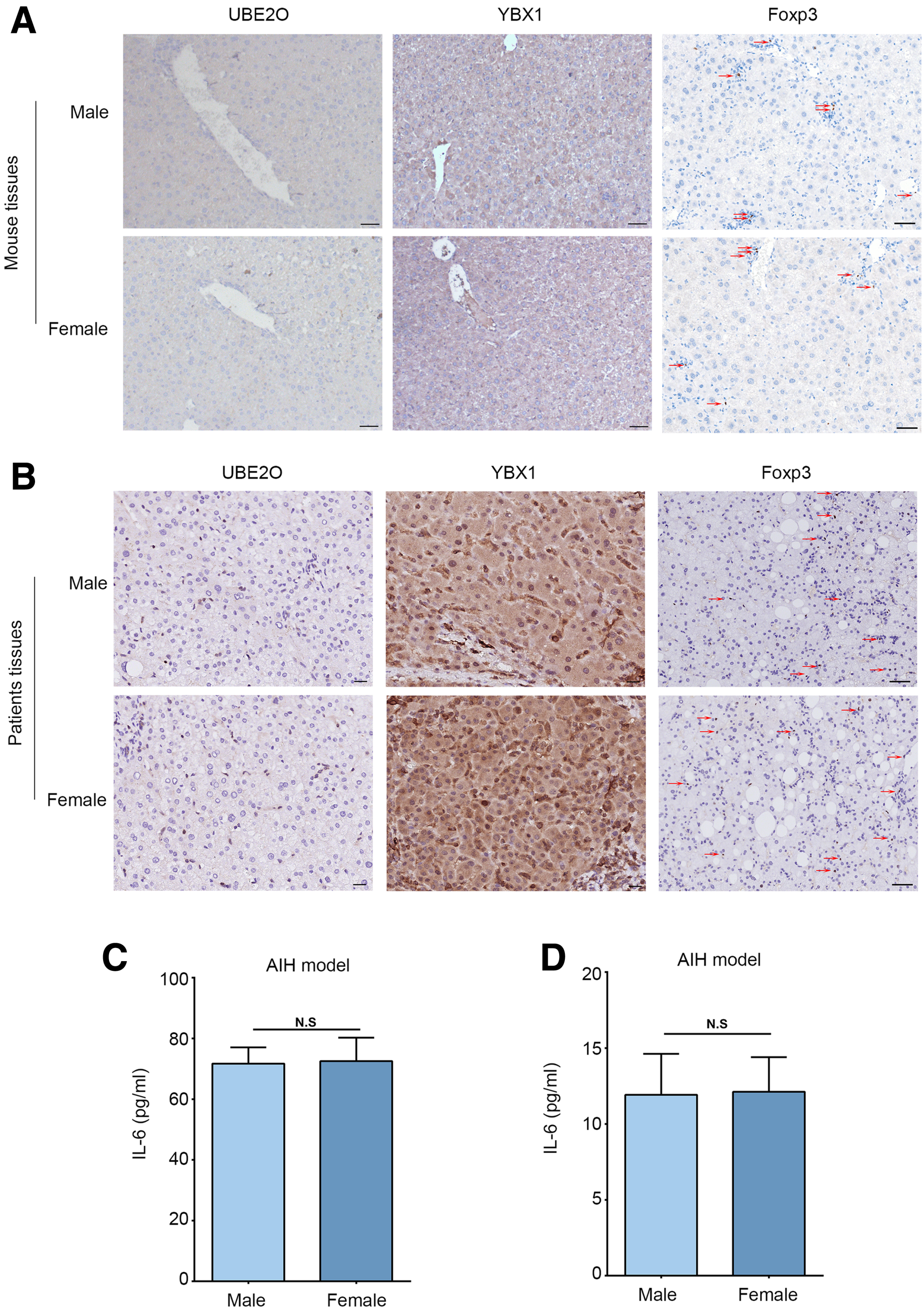

Figure 10. Comparison of UBE2O/YBX1/IL-6 expression and Tregs between sexes in AIH. (*A*) Representative IHC staining images showing UBE2O, YBX1, and Foxp3 expression in hepatic tissues of male and female chronic AIH mice. *Red arrows* indicate Foxp3^{+}cells. Bars, 20 μm (*left 2 columns*); 50 μm (*right column*). (*B*) Representative IHC staining images showing UBE2O, YBX1, and Foxp3 expression in hepatic tissues of male and female patients with AIH. *Red arrows* indicate Foxp3^{+}cells. Bars, 20 μm (*left 2 columns*); 50 μm (*right column*). (*C*) ELISA analysis of serum IL-6 levels of male and female chronic AIH mice. N.S, no significance. Mean ± SEM. Student's *t*-test. (*D*) ELISA analysis of serum IL-6 levels of male and female patients with AIH. N.S, no significance. Mean ± SEM. Student's *t*-test.

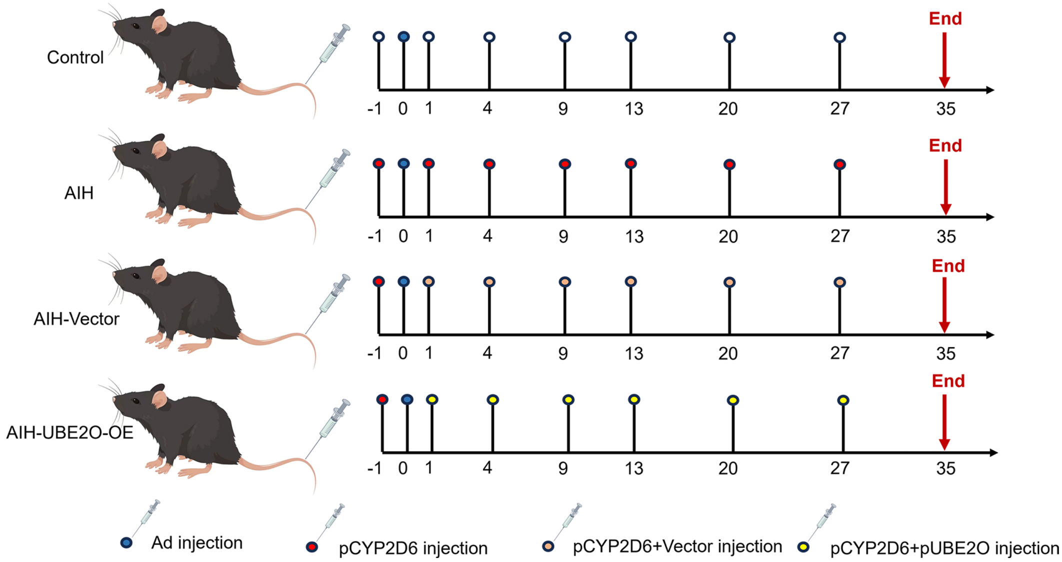

Figure 11. Diagrams for establishment of murine models. (*A*) Experimental strategy to induce chronic murine AIH models in the indicated groups.

required to clarify other potential downstream pathways regulated by UBE2O and to understand how hepatic UBE2O expression is downregulated in AIH. Investigating whether YBX1 or UBE2O mutations is involved in patients with AIH will be important to better understand their contribution to AIH susceptibility. Importantly, further work is also needed to identify the crosstalk between immune cells and hepatocellular UBE2O.

In conclusion, this study provides evidence to demonstrate the hepatoprotective role of UBE2O in AIH and sheds light on an important mechanistic link between the hepatocellular UBE2O/YBX1/IL-6 axis and hepatic Treg/Th17 immune homeostasis. UBE2O overexpression promotes ubiquitination degradation of YBX1 and inhibits IL-6 secreted by hepatocytes, thus restoring hepatic Treg/Th17 balance and attenuating liver injury in AIH. These findings underscore the significance of the ubiquitin-binding enzyme UBE2O in modulating immune homeostasis in AIH. Furthermore, the present study expands the current knowledge of the crosstalk between hepatocytes as innate immune cells and adaptive immune cells, offering new perspectives for clinical intervention.

Materials and Methods

Animals and the Experimental Protocol

Specific pathogen-free (SPF) male C57BL/6 mice (6–8 weeks old; 18–20 g) were purchased from Gempharmatech Co, Ltd. The protocol was approved by the Committee of Ethics of Animal Experiments of Tongji Hospital, Huazhong University of Science and Technology. As previously described, the chronic AIH model was established by overexpressing human autoantigen cytochrome P450 2D6 (CYP2D6) in mice. The plasmid expressing human CYP2D6 (pCYP2D6) was preserved in our institute. On day 0, mice were infected with adenovirus through tail vein injection to promote the induction of AIH using human CYP2D6. Then, pCYP2D6 plasmid was administered at days 1, 4, 9, 13, 20, and 27 via a rapid tail vein injection (50 μg per injection) to transfect human CYP2D6 into livers using the hydrodynamic-based liver-targeted gene delivery technique. Plasmid injection was also performed once at 1 day before adenovirus injection to enhance immunogenicity.[18,43] Plasmid pUBE2O or pYBX1was injected together with pCYP2D6 to overexpress hepatic UBE2O in chronic AIH models. An empty vector was used as the control (Figure 11). For clodronate liposomes treatment, mice received clodronate liposomes (100 μL per mouse) by intraperitoneal injection every other day from day 14 of AIH induction. The mice were then sacrificed on day 35 for analysis.

Human Samples

The human liver samples were obtained from patients with AIH who underwent liver biopsies at Tongji Hospital, and the diagnosis was confirmed pathologically. Samples of the control group were obtained from those diagnosed as hepatic hemangioma who underwent surgical resection at Tongji Hospital. Baseline characteristics of the non-AIH controls and patients with AIH are shown in Table 3. This study was approved by the Ethics Committee of Tongji Hospital and complied with the World Medical Association Declaration of Helsinki. Written consent was obtained from the patients.

Cell Line and Reagents

The AML12 hepatocyte cell line and RAW264.7 cells were kept in the Institute of Liver and Gastrointestinal Diseases (Tongji Hospital, Huazhong University of Science and Technology). The AML12 cells were cultured in Dulbecco's Modified Eagle's Medium (DMEM)/F12 medium and RAW264.7

cells were cultured in DMEM medium. The cell lines were incubated at 37°C with 5% CO_2. Ten percent fetal bovine serum (FBS) (Invitrogen Gibco) was added into the media. LPS was purchased from Sigma-Aldrich. Recombinant murine IL-2 was purchased from R&D systems, and recombinant murine TGF-β, anti-IFN-γ antibody, anti-IL-4 antibody, and anti-IL-6 antibody were purchased from Biolegend. Clodronate liposomes was purchased from MedChemExpress.

Histopathology and IHC

Liver tissues from the median and left lobes were harvested and were cut and fixed in 4% paraformaldehyde for at least 24 hours, embedded in paraffin, and cut at a thickness of 5 μm. Then they were stained with H&E and Sirius red to detect hepatic inflammation and fibrosis. The calculation of liver inflammatory scores has been described previously.[18] The liver inflammatory score was assessed using the Knodell histological activity index (Knodell HAI), a hepatitis scoring system.[44] Briefly, the system includes the following components: (1) periportal necrosis/interface hepatitis (0–10); (2) intralobular degeneration and focal spotty necrosis (0–4); (3) portal fibrosis (0–4); (4) septal/bridging fibrosis and cirrhosis (0–4). The total score ranges from 0 to 22, with higher values indicating greater inflammatory activity and more advanced fibrosis. Five-μm thick liver sections dewaxed in xylene and rehydrated in graded ethanol solutions were used for IHC to detect Tregs and Th17 cells, YBX1, and UBE2O expression in the liver, with anti-UBE2O (cat#A17201, Abclonal) for human, anti-UBE2O (cat#A10036, Abclonal) for mouse, anti-YBX1 (cat#20339-1-AP, Proteintech), anti-Foxp3 (cat#12653, Cell Signaling Technology) for mouse, anti-Foxp3 (cat#98377, Cell Signaling Technology) for human, anti-IL-17A (cat#26163-1-AP, Proteintech) as the primary antibody. The intensity of IHC staining was quantified by the mean gray value in defined regions of interest using ImageJ software, and the relative intensity units were further compared. The detailed procedure was described in our previous study.[45]

Biochemistry and Inflammatory Cytokines Measurement

The venous blood of mice was collected and centrifuged at 35,000 rpm for 10 minutes. The serum levels of AST and ALT, in the collected mouse sera, were determined using an automatic biochemistry analysis apparatus at the Clinical Laboratory of Tongji Hospital. The cytokines (IL-2, IL-4, IL-6, IFN-γ, TNF-α, IL-17A, and IL-10) in the serum were detected by flow cytometry using a mouse cytometric bead array (CBA) kit (cat#560485, BD Biosciences).

Western Blot and qRT-PCR

The primary antibodies used for Western blot were anti-UBE2O (cat#A10036, Abclonal); anti-YBX1 (cat#ab76149, Abcam); anti-IL-6 (cat#12912, Cell signaling technology); and anti-β-actin (cat#66009-1-Ig, Proteintech). A horseradish peroxidase-conjugated secondary antibody (Promoter Biotechnology Ltd) was also used. The expression of the antibody-linked proteins was determined using a Super ECL Detection Reagent (cat#E422-01S60, Vazyme). The total RNA extraction was conducted using FastPure Cell/Tissue Total RNA Isolation Kit (cat#R112-01, Vazyme), and reverse transcription was performed using HiScript IV RT SuperMix for qPCR (cat#R423, Vazyme). The relative mRNA levels were evaluated by qPCR using the One Step RT-qPCR SYBR Green Kit (cat#11143, YEASEN). The primers used are listed in Table 1. The detailed procedure was described previously.[46]

ELISA Analysis

The collected cell supernatant was used to detect the expression of IL-6 level by an IL-6 ELISA kit (cat#EK0411, Boster) following the manufacturer's instructions.

Isolation of Liver Mononuclear Cells

As a previous study described, an approach combining collagenase digestion and gradient centrifugation was used to isolate mouse primary hepatocytes and mononuclear cells (MNCs). The peritoneal cavity of mice was opened after anesthesia. The inferior vena cava was intubated and cut off. The liver was first perfused with D-Hank's buffer containing 0.5 mmol/L ethylenediaminetetraacetic acid (EDTA) at 37°C for 10 minutes, and then slowly perfused with 0.05% Collagen IV (Sigma-Aldrich) buffer to digest the liver for 10 minutes. The liver was removed, placed in sterile dishes, and bluntly separated into several small pieces. The precipitate was then filtered and centrifuged at 50 × g for 1 minute 3 times. DMEM containing 1% penicillin-streptomycin solution (Beyotime Biotechnology) and 10% FBS was added to obtain primary hepatocytes. For MNC isolation, the filtrate was centrifuged at 20 × g for 5 minutes and then washed in DMEM/F12. Resuspended cells were treated with 30% Percoll (Sigma-Aldrich) and then gently stacked onto 70% Percoll. MNCs were harvested from the Percoll gradient interface after centrifugation at a density gradient of 1000 × g for 30 minutes, and collected for further analysis.

Table 1. Primers Used for qRT-PCR

Primer	Sequence
Ube2o	Forward: 5′-GCGTCTCGGATGCTTTGAC-3′<br>Reverse: 5′-CACTGGGTATCTGGGGTGC-3′
Il-6	Forward: 5′-TCTATACCACTTCACAAGTCGGA-3′<br>Reverse: 5′-GAATTGCCATTGCACAACTCTTT-3′
Ccl5	Forward: 5′-TGCCCACGTCAAGGAGTATTTC-3′<br>Reverse: 5′-CCTCGTAGGACAGGAAGATCTC-3′
Cxcl9	Forward: 5′-TGCAGACCATGAAGGAACTGCT-3′<br>Reverse: 5′-TGGTCTTTGAGGGATTTGTGGT-3′
Cxcl10	Forward: 5′-CTTGCCTGCTGGGATGACAAC-3′<br>Reverse: 5′-GCTTCCCTATGGCCCTCATT-3′
Actb	Forward: 5′-GTGACGTTGACATCCGTAAAGA-3′<br>Reverse: 5′- GCCGGACTCATCGTACTCC-3′

qRT-PCR, quantitative real-time-polymerase chain reaction.

Table 2. Primer Sequences Used for Mutagenesis

Primer name	Primer sequences
mYBX1-K62R	Forward: 5′-GCAACGAAGGTTTTGGGAACAGTCAGATGGTTCAATGTAAG-3′<br>Reverse: 5′-CTGACTGTTCCCAAAACCTTCGTTGCGATGACCTTCTTGTC-3′
mYBX1-K79R	Forward: 5′-CGGTTTCATCAACAGGAATGACACCAGGGAAGACGTATTTG-3′<br>Reverse: 5′-CTGGTGTCATTCCTGTTGATGAAACCGTATCCGTTCCTTAC-3′
mYBX1-K90R	Forward: 5′-GTATTTGTACACCAGACTGCCATAAGGAAGAATAACCCCAG-3′<br>Reverse: 5′-CTTATGGCAGTCTGGTGTACAAATACGTCTTCCTTGGTGTC-3′
mYBX1-K135R	Forward: 5′-CTGGTGGAGTTCCAGTTCAAGGCAGTAGATACGCAGCAGACCG-3′<br>Reverse: 5′-CTACTGCCTTGAACTGGAACTCCACCAGGGCCTGTAACATTTG-3′

Isolation and Culture of Naïve CD4$^+$T Cells

Mice spleens were ground and filtered with a 70-μm cell strainer in phosphate-buffered saline (PBS). After the elimination of erythrocytes by Red Blood Cell Lysis Buffer (Sigma-Aldrich), splenocytes were washed and resuspended in PBS. Per 10^7 cells were resuspended with 40 μL of sorting buffer. The next steps were according to the manufacturer's instructions by a mouse Naïve CD4$^+$T Cell Isolation Kit (cat#130-104-453, Miltenyi Biotec). The magnetic bead-sorted naïve CD4$^+$T cells were seeded in a U-bottomed 96-well plate (1×10^5/well). One μl of anti-CD3/CD28 beads, recombinant murine IL-2 (100 U/mL), 5 μg/mL anti-IL-4 antibody (5 μg/mL), anti-IFN-γ antibody (5 μg/mL), and recombinant murine transforming growth factor β (TGF-β; 1 ng/mL) were added to each well and then for further analysis.

Plasmids, siRNA, and Adenoviruses

The pcDNA3.1 was used to generate the vector carrying human CYP2D6, mouse UBE2O, and mouse YBX1. The mutant YBX1 plasmids were cloned in pcDNA3.1 with HA-tag, and the sequences of cloning primers are listed in Table 2. The sequence for siRNA was listed as follows: si*Ube2o*, 5′-TGAAGAGCCTGAAGATGTT-3′, and the control sequence was: 5′-TTCTCCGAACGTGTCACGT-3′. Plasmid or siRNA transfection was performed with a standard method. The siRNA was provided by RiboBio Co, Ltd, and the plasmids and adenoviruses used were provided by DesignGene Biotechnology Company.

Table 3. Baseline Characteristics of the Non-AIH Controls and Patients With AIH

	Control group (n = 10)	AIH group (n = 14)
Age, *y*	39.7 ± 3.249	34.5 ± 3.092
Sex (male/female)	3/7	3/11
ALT, *IU/L*	25.1 ± 3.10	165.4 ± 36.75[b]
AST, *IU/L*	38.1 ± 6.25	151.2 ± 32.97[b]
IgG (+)	0 (0)	9 (64.3)
ANA (≥1:100)	0 (0)	11 (78.5)
Interface hepatitis	0 (0)	9 (64.3)
Rosettes	0 (0)	4 (28.6)
Lymphocyte invasion	0 (0)	6 (42.9)

NOTE. Data are presented as mean ± standard error of the mean or number (%).
[a]Significantly different from control group.
AIH, autoimmune hepatitis; ALT, alanine aminotransferase; ANA, antinuclear antibodies; AST, aspartate aminotransferase.
[b]$P < .01$ (unpaired *t*-test).

Luciferase Reporter Assay

The promoter region (−2000 to +200) of corresponding genes was cloned into the pGL3-Basic luciferase reporter vector. The luciferase reporter vector, Renilla luciferase reporter plasmid, and the indicated plasmids in different experimental groups were co-transfected into AML12 cells. Forty-eight hours later, luciferase activity was analyzed using a Luciferase Reporter Gene Assay Kit (cat#11401ES60, YEASEN) following the manufacturer's instructions. Renilla luciferase activity was used as a normalization control.

Flow Cytometry

The isolated MNCs (at least 10^6 cells/tube) were resuspended in PBS. Anti-mouse CD16/CD32 (cat#101319, Biolegend) was used to reduce nonspecific fluorescent staining. Then, the cells were stained with fluorochrome-conjugated antibodies: allophycocyanin (APC)-conjugated anti-mouse CD25 (cat#557192, BD Pharmingen), fluorescein isothiocyanate (FITC)-conjugated anti-mouse CD4 (cat#557307, BD Pharmingen), BV421-conjugated anti-mouse Foxp3 (cat#562996, BD Pharmingen) and phycoerythrin (PE)-conjugated anti-mouse IL-17A (cat#559502, BD Pharmingen). The resuspended cells were finally analyzed by fluorescence-activated cell sorting (FACS). Data were analyzed by using a FlowJo software package (TreeStar).

IF Assays

For IF experiments, hepatocytes were fixed with 4% paraformaldehyde at room temperature for 15 minutes. Tissue sections or fixed cells were permeabilized with PBS containing 0.3% Triton X-100 for 10 minutes, blocked with 10% goat serum, and incubated overnight at 4°C with primary antibodies such as YBX1 (cat#ab76149, Abcam), and anti-UBE2O (cat#A10036, Abclonal). Following washing 3 times with PBS, the samples underwent incubation with the appropriate secondary antibodies (Promoter). 4,6-diamidino-2-phenylindone

(DAPI) was used for nuclei staining. Fluorescence detection was conducted with an Olympus fluorescence microscope.

Co-IP Assay

For Co-IP assays, the AML12 hepatocytes were transfected with indicated plasmids or siRNA. Then, the cells were lysed in cold NP-40 immunoprecipitation buffer for 1 hour. Following centrifugation, the supernatant was combined with the selected antibodies including YBX1 (cat#20339-1-AP, Proteintech), UBE2O (cat#A10036, Abclonal), HA-tag (Proteintech, cat#66006-2-Ig), Flag-tag (Proteintech, cat#66008-4-Ig), Ub (Cell signaling technology, cat#20326), K48-Ub (Cell signaling technology, cat#8081), and Normal IgG (Santa Cruz, cat#sc-2025) overnight at 4°C. Then, Protein A/G Magnetic Beads (cat#HY-K0202, MedChemExpress) were added into the supernatants. After incubation at 4°C for 4 hours, magnetic beads precipitated with proteins were separated from the mixture. The precipitated proteins were washed with lysis buffer 3 times. Samples were then prepared for subsequent Western blotting analyses or MS analysis.

MS Analysis

AML12 hepatocytes were transfected with pUBE2O for 72 hours and harvested. Extractions of cell samples were processed for immunoprecipitation as described above. The immunoprecipitates were separated by sodium dodecyl-sulfate polyacrylamide gel electrophoresis (SDS-PAGE). The gel bands were proteolyzed and dried. The dried peptide samples were then reconstituted and centrifuged. The supernatants were taken for MS detection. The results of protein identification were obtained by aligning experimental MS/MS data with theoretical values from databases, such as the UniProt protein database (https://www.uniprot.org/) (Supplementary Table 1). MS analysis was conducted by Bioyi Technology Co, Ltd.

Statistical Analysis

Data are presented as mean ± standard error of the mean (SEM). Statistical analyses were conducted using GraphPad Prism software (version 6.0). Comparisons were performed using the Student's *t*-test, or analysis of variance (ANOVA). Fisher's exact test was used for the categorical variables. Correlation was assessed using Spearman correlation analysis. A 2-sided $P < .05$ was considered statistically significant.

Supplementary Material

Note: To access the supplementary material accompanying this article, visit the full text version at https://doi.org/10.1016/j.jcmgh.2026.101765.

References

1. Sirbe C, Simu G, Szabo I, et al. Pathogenesis of autoimmune hepatitis-cellular and molecular mechanisms. Int J Mol Sci 2021;22:13578.
2. Muratori L, Lohse AW, Lenzi M. Diagnosis and management of autoimmune hepatitis. BMJ 2023;380:e070201.
3. Katsumi T, Ueno Y. Epidemiology and surveillance of autoimmune hepatitis in Asia. Liver Int 2022;42:2015–2022.
4. Cheng ML, Nakib D, Perciani CT, MacParland SA. The immune niche of the liver. Clin Sci (Lond) 2021; 135:2445–2466.
5. Sakaguchi S, Mikami N, Wing JB, et al. Regulatory T cells and human disease. Annu Rev Immunol 2020; 38:541–566.
6. Wang H, Feng X, Yan W, Tian D. Regulatory T cells in autoimmune hepatitis: unveiling their roles in mouse models and patients. Front Immunol 2020;11: 575572.
7. Huang Z, Nie S, Wang H, et al. The proportion of regulatory T cells in peripheral blood of patients with autoimmune hepatitis: a systematic review and meta-analysis. Int Immunopharmacol 2023;122:110576.
8. Knochelmann HM, Dwyer CJ, Bailey SR, et al. When worlds collide: Th17 and Treg cells in cancer and autoimmunity. Cell Mol Immunol 2018;15:458–469.
9. Liu Y, Yan W, Yuan W, et al. Treg/Th17 imbalance is associated with poor autoimmune hepatitis prognosis. Clin Immunol 2019;198:79–88.
10. Chen H, Han Z, Fan Y, et al. CD4+ T-cell subsets in autoimmune hepatitis: a review. Hepatol Commun 2023; 7:e0269.
11. Wang H, Feng X, Han P, et al. The JAK inhibitor tofacitinib ameliorates immune-mediated liver injury in mice. Mol Med Rep 2019;20:4883–4892.
12. Popovic D, Vucic D, Dikic I. Ubiquitination in disease pathogenesis and treatment. Nat Med 2014;20:1242–1253.
13. Yu H, Yang W, Cao M, et al. Mechanism study of ubiquitination in T cell development and autoimmune disease. Front Immunol 2024;15:1359933.
14. Ma M, Zhang C, Cao R, et al. UBE2O promotes lipid metabolic reprogramming and liver cancer progression by mediating HADHA ubiquitination. Oncogene 2022; 41:5199–5213.
15. Huang Q, Qin D, Pei D, et al. UBE2O and USP7 co-regulate RECQL4 ubiquitinylation and homologous recombination-mediated DNA repair. FASEB J 2022;36: e22112.
16. Chen S, Yang J, Zhang Y, et al. Ubiquitin-conjugating enzyme UBE2O regulates cellular clock function by promoting the degradation of the transcription factor BMAL1. J Biol Chem 2018;293:11296–11309.
17. Zhang X, Zhang J, Zhang L, et al. UBE2O negatively regulates TRAF6-mediated NF-kappaB activation by inhibiting TRAF6 polyubiquitination. Cell Res 2013;23:366–377.
18. Wang H, Yan W, Feng Z, et al. Plasma proteomic analysis of autoimmune hepatitis in an improved AIH mouse model. J Transl Med 2020;18:3.
19. Longhi MS, Zhang L, Mieli-Vergani G, Vergani D. B and T cells: (Still) the dominant orchestrators in autoimmune hepatitis. Autoimmun Rev 2024;23:103591.
20. Kimura A, Kishimoto T. IL-6: regulator of Treg/Th17 balance. Eur J Immunol 2010;40:1830–1835.
21. Korn T, Hiltensperger M. Role of IL-6 in the commitment of T cell subsets. Cytokine 2021;146:155654.
22. Rybalkina EY, Moiseeva NI. Role of YB-1 Protein in Inflammation. Biochemistry (Mosc) 2022;87:S94–S202.

23. Castellana B, Aasen T, Moreno-Bueno G, et al. Interplay between YB-1 and IL-6 promotes the metastatic phenotype in breast cancer cells. Oncotarget 2015;6:38239–38256.
24. Li X, Chen G, Liu B, et al. PLK1 inhibition promotes apoptosis and DNA damage in glioma stem cells by regulating the nuclear translocation of YBX1. Cell Death Discov 2023;9:68.
25. Zhang HR, Li TJ, Yu XJ, et al. The GFPT2-O-GlcNAcylation-YBX1 axis promotes IL-18 secretion to regulate the tumor immune microenvironment in pancreatic cancer. Cell Death Dis 2024;15:244.
26. Chaouali M, Azaiez MB, Tezeghdenti A, et al. Association of TNF-alpha-308 polymorphism with susceptibility to autoimmune hepatitis in Tunisians. Biochem Genet 2018;56:650–662.
27. Czaja AJ. Immune inhibitory properties and therapeutic prospects of transforming growth factor-beta and interleukin 10 in autoimmune hepatitis. Dig Dis Sci 2022; 67:1163–1186.
28. Czaja AJ. Immune inhibitory proteins and their pathogenic and therapeutic implications in autoimmunity and autoimmune hepatitis. Autoimmunity 2019;52:144–160.
29. Shojaie L, Ali M, Iorga A, Dara L. Mechanisms of immune checkpoint inhibitor-mediated liver injury. Acta Pharm Sin B 2021;11:3727–3739.
30. Gong J, Tu W, Liu J, Tian D. Hepatocytes: a key role in liver inflammation. Front Immunol 2022;13:1083780.
31. Hunter CA, Jones SA. IL-6 as a keystone cytokine in health and disease. Nat Immunol 2015;16:448–457.
32. Norris CA, He M, Kang LI, et al. Synthesis of IL-6 by hepatocytes is a normal response to common hepatic stimuli. PLoS One 2014;9:e96053.
33. Chi G, Feng XX, Ru YX, et al. TLR2/4 ligand-amplified liver inflammation promotes initiation of autoimmune hepatitis due to sustained IL-6/IL-12/IL-4/IL-25 expression. Mol Immunol 2018;99:171–181.
34. Naseem S, Hussain T, Manzoor S. Interleukin-6: a promising cytokine to support liver regeneration and adaptive immunity in liver pathologies. Cytokine Growth Factor Rev 2018;39:36–45.
35. Li R, Li D, Nie Y. IL-6/gp130 signaling: a key unlocking regeneration. Cell Regen 2023;12:16.
36. Giraldez MD, Carneros D, Garbers C, et al. New insights into IL-6 family cytokines in metabolism, hepatology and gastroenterology. Nat Rev Gastroenterol Hepatol 2021; 18:787–803.
37. Murakami S, Imamura M, Uchida T, et al. Serum interleukin-6 level predicts the prognosis for patients with alcohol-related acute-on-chronic liver failure. Hepatol Int 2023;17:1225–1232.
38. Li SQ, Zhu S, Han HM, et al. IL-6 trans-signaling plays important protective roles in acute liver injury induced by acetaminophen in mice. J Biochem Mol Toxicol 2015; 29:288–297.
39. Yau R, Rape M. The increasing complexity of the ubiquitin code. Nat Cell Biol 2016;18:579–586.
40. Martin M, Hua L, Wang B, et al. Novel serine 176 phosphorylation of YBX1 activates NF-kappaB in colon cancer. J Biol Chem 2017;292:3433–3444.
41. Dong W, Wang H, Shahzad K, et al. Activated protein C ameliorates renal ischemia-reperfusion injury by restricting Y-box binding protein-1 ubiquitination. J Am Soc Nephrol 2015;26:2789–2799.
42. Ji Y, Xu Z, Tang L, et al. O-GlcNAcylation of YBX1 drives a glycolysis-histone lactylation feedback loop in hepatocellular carcinoma. Cancer Lett 2025;631:217957.
43. Wang H, Wang S, Lei Y, et al. Serum amyloid A1-induced intrahepatic regulatory T-cell dysfunction drives autoimmune hepatitis progression. Published online June 9, 2025. https://doi.org/10.1097/HEP.0000000000001419.
44. Knodell RG, Ishak KG, Black WC, et al. Formulation and application of a numerical scoring system for assessing histological activity in asymptomatic chronic active hepatitis. Hepatology 1981;1:431–435.
45. Lei Y, Han P, Chen Y, et al. Protein arginine methyltransferase 3 promotes glycolysis and hepatocellular carcinoma growth by enhancing arginine methylation of lactate dehydrogenase A. Clin Transl Med 2022;12:e686.
46. Chen Y, Lei Y, Wang H, et al. Sophoricoside attenuates autoimmune-mediated liver injury through the regulation of oxidative stress and the NF-kappaB signaling pathway. Int J Mol Med 2023;52:78.

Received August 16, 2025. Accepted February 24, 2026.

Correspondence
Address correspondence to: Dean Tian, MD, PhD, Department of Gastroenterology, Tongji Hospital, Tongji Medical College, Huazhong University of Science and Technology, Jiefang Avenue 1095, Wuhan, Hubei Province 430030, China. e-mail: datian@tjh.tjmu.edu.cn; or Mei Liu, MD, PhD, Department of Gastroenterology, Tongji Hospital, Tongji Medical College, Huazhong University of Science and Technology, Jiefang Avenue 1095, Wuhan, Hubei Province 430030, China. e-mail: fliumei@126.com.

Acknowledgments
The authors thank the experimental instrument support (Fluorescence Activating Cell Sorter support) from Experimental Medicine Center, Tongji Hospital, Tongji Medical College, Huazhong University of Science and Technology. The authors thank the MS service assistance from Bioyi Biotechnology Co, Ltd, Wuhan, China.

CRedit Authorship Contributions
Yu Lei (Conceptualization: Equal; Data curation: Lead; Investigation: Equal; Methodology: Equal; Validation: Lead; Visualization: Equal; Writing – original draft: Equal)

Han Wang (Conceptualization: Equal; Funding acquisition: Supporting; Investigation: Equal; Methodology: Equal; Visualization: Equal; Writing – original draft: Equal)

Yu Chen (Investigation: Supporting; Methodology: Supporting)

Shuhui Wang (Investigation: Supporting; Methodology: Supporting)

Zhipeng Du (Data curation: Supporting; Methodology: Supporting)

Zheng Huang (Data curation: Supporting; Methodology: Supporting)

Muru Wang (Investigation: Supporting; Methodology: Supporting)

Shangshu Nie (Formal analysis: Supporting; Investigation: Supporting)

Ping Han (Data curation: Supporting; Formal analysis: Supporting)

Wei Yan (Data curation: Supporting; Formal analysis: Supporting)

Mei Liu (Formal analysis: Lead; Investigation: Equal; Methodology: Equal; Supervision: Equal; Writing – review & editing: Equal)

Dean Tian (Conceptualization: Lead; Data curation: Lead; Funding acquisition: Lead; Investigation: Equal; Methodology: Equal; Supervision: Equal; Writing – review & editing: Equal)

Conflicts of interest
The authors disclose no conflicts.

Funding
The work was supported by grants from the National Natural Science Foundation of China (No. 81974071, 82270558, 82200588, and 82470561), and China Postdoctoral Science Foundation (No. 2025M782000).

Data Availability
The data presented in this study are available on request from the corresponding authors.

Cellular and Molecular Gastroenterology and Hepatology, Volume *20*

Supplemental information

Ubiquitin-Conjugating Enzyme E2O Primes Hepatocytes to Restore Immune Tolerance in Autoimmune Hepatitis via Inhibiting Y-Box Binding Protein 1/Interleukin-6 Axis

Yu Lei, Han Wang, Yu Chen, Shuhui Wang, Zhipeng Du, Zheng Huang, Muru Wang, Shangshu Nie, Ping Han, Wei Yan, Mei Liu, and Dean Tian

Table S1. Potential proteins showing in mass spectrometry that may interact with Ube2o

Accession	Gene	Description	iBAQ IP [%]
**P99024**	Tubb5	Tubulin beta-5 chain OS=Mus musculus OX=10090 GN=Tubb5 PE=1 SV=1	0.59822702
**P68372**	Tubb4b	Tubulin beta-4B chain OS=Mus musculus OX=10090 GN=Tubb4b PE=1 SV=1	0.844459314
**P11983**	Tcp1	T-complex protein 1 subunit alpha OS=Mus musculus OX=10090 GN=Tcp1 PE=1 SV=3	0.159140979
**P14869**	Rplp0	60S acidic ribosomal protein P0 OS=Mus musculus OX=10090 GN=Rplp0 PE=1 SV=3	0.272813107
**Q6A0A9**	FAM120A	Constitutive coactivator of PPAR-gamma-like protein 1 OS=Mus musculus OX=10090 GN=FAM120A PE=1 SV=2	0.153046016
**Q8CGC7**	Eprs	Bifunctional glutamate/proline--tRNA ligase OS=Mus musculus OX=10090 GN=Eprs PE=1 SV=4	0.030899666
**P70168**	Kpnb1	Importin subunit beta-1 OS=Mus musculus OX=10090 GN=Kpnb1 PE=1 SV=2	0.125655715
**P17742**	Ppia	Peptidyl-prolyl cis-trans isomerase A OS=Mus musculus OX=10090 GN=Ppia PE=1 SV=2	0.596110713
**P09405**	Ncl	Nucleolin OS=Mus musculus OX=10090 GN=Ncl PE=1 SV=2	1.288460477
**Q99KI0**	Aco2	Aconitate hydratase, mitochondrial OS=Mus musculus OX=10090 GN=Aco2 PE=1 SV=1	0.079890581
**Q8C2Q7**	Hnrnph1	Heterogeneous nuclear ribonucleoprotein H OS=Mus musculus OX=10090 GN=Hnrnph1 PE=1 SV=1	0.277162118
**Q5SUT0**	Ewsr1	RNA-binding protein EWS OS=Mus musculus OX=10090 GN=Ewsr1 PE=1 SV=1	0.130512639
**Q61316**	Hspa4	Heat shock 70 kDa protein 4 OS=Mus musculus OX=10090 GN=Hspa4 PE=1 SV=1	0.108820495
**P26039**	Tln1	Talin-1 OS=Mus musculus OX=10090 GN=Tln1 PE=1 SV=2	0.01707066
**Q9JKF1**	Iqgap1	Ras GTPase-activating-like protein IQGAP1 OS=Mus musculus OX=10090 GN=Iqgap1 PE=1 SV=2	0.05028874
**P27659**	Rpl3	60S ribosomal protein L3 OS=Mus musculus OX=10090 GN=Rpl3 PE=1 SV=3	0.551774086
**Q9Z1Q9**	Vars	Valine--tRNA ligase OS=Mus musculus OX=10090 GN=Vars PE=1 SV=1	0.102429248
**Q9JIK5**	Ddx21	Nucleolar RNA helicase 2 OS=Mus musculus OX=10090 GN=Ddx21 PE=1 SV=3	0.140459281

**P08113**	Hsp90b1	Endoplasmin OS=Mus musculus OX=10090 GN=Hsp90b1 PE=1 SV=2	0.204990766
**Q8VEK3**	Hnrnpu	Heterogeneous nuclear ribonucleoprotein U OS=Mus musculus OX=10090 GN=Hnrnpu PE=1 SV=1	0.803984947
**E9PVC6**	Eif4g1	Eukaryotic translation initiation factor 4 gamma 1 OS=Mus musculus OX=10090 GN=Eif4g1 PE=1 SV=1	0.03475399
**Q8BGJ5**	Ptbp1	MCG13402, isoform CRA_a OS=Mus musculus OX=10090 GN=Ptbp1 PE=1 SV=1	0.198017535
**P14148**	Rpl7	60S ribosomal protein L7 OS=Mus musculus OX=10090 GN=Rpl7 PE=1 SV=2	0.338148788
**O35286**	Dhx15	Pre-mRNA-splicing factor ATP-dependent RNA helicase DHX15 OS=Mus musculus OX=10090 GN=Dhx15 PE=1 SV=2	0.101492783
**P11499**	Hsp90ab1	Heat shock protein HSP 90-beta OS=Mus musculus OX=10090 GN=Hsp90ab1 PE=1 SV=3	2.422324743
**Q02053**	Uba1	Ubiquitin-like modifier-activating enzyme 1 OS=Mus musculus OX=10090 GN=Uba1 PE=1 SV=1	0.13755465
**P17182**	Eno1	Alpha-enolase OS=Mus musculus OX=10090 GN=Eno1 PE=1 SV=3	0.553096778
**A0A1B0GQU8**	Rpl18	60S ribosomal protein L18 OS=Mus musculus OX=10090 GN=Rpl18 PE=1 SV=1	1.056936515
**Q9WTI7**	Myo1c	Unconventional myosin-Ic OS=Mus musculus OX=10090 GN=Myo1c PE=1 SV=2	0.022019643
**P26040**	Ezr	Ezrin OS=Mus musculus OX=10090 GN=Ezr PE=1 SV=3	0.443900638
**P45376**	Akr1b1	Aldo-keto reductase family 1 member B1 OS=Mus musculus OX=10090 GN=Akr1b1 PE=1 SV=3	0.029521421
**Q61753**	Phgdh	D-3-phosphoglycerate dehydrogenase OS=Mus musculus OX=10090 GN=Phgdh PE=1 SV=3	0.386099009
**P13020**	Gsn	Gelsolin OS=Mus musculus OX=10090 GN=Gsn PE=1 SV=3	0.26896672
**P10107**	Anxa1	Annexin A1 OS=Mus musculus OX=10090 GN=Anxa1 PE=1 SV=2	0.511040471
**P62960**	Ybx1	Nuclease-sensitive element-binding protein 1 OS=Mus musculus OX=10090 GN=Ybx1 PE=1 SV=3	0.315620703
**P58252**	Eef2	Elongation factor 2 OS=Mus musculus OX=10090 GN=Eef2 PE=1 SV=2	0.867897411

**Q05D44**	Eif5b	Eukaryotic translation initiation factor 5B OS=Mus musculus OX=10090 GN=Eif5b PE=1 SV=2	0.107180357
**P20029**	Hspa5	Endoplasmic reticulum chaperone BiP OS=Mus musculus OX=10090 GN=Hspa5 PE=1 SV=3	0.519680293
**P29341**	Pabpc1	Polyadenylate-binding protein 1 OS=Mus musculus OX=10090 GN=Pabpc1 PE=1 SV=2	0.462381287
**A2BH06**	Rpl11	60S ribosomal protein L11 (Fragment) OS=Mus musculus OX=10090 GN=Rpl11 PE=1 SV=1	0.252306095
**A0A2I3BQF4**	Rpl30	60S ribosomal protein L30 OS=Mus musculus OX=10090 GN=Rpl30 PE=1 SV=1	0.161289031
**P63038**	Hspd1	60 kDa heat shock protein, mitochondrial OS=Mus musculus OX=10090 GN=Hspd1 PE=1 SV=1	0.49364972
**P52293**	Kpna2	Importin subunit alpha-1 OS=Mus musculus OX=10090 GN=Kpna2 PE=1 SV=2	0.0998209
**P52480**	Pkm	Pyruvate kinase PKM OS=Mus musculus OX=10090 GN=Pkm PE=1 SV=4	1.409566132
**B1AXW5**	Prdx1	Peroxiredoxin-1 (Fragment) OS=Mus musculus OX=10090 GN=Prdx1 PE=1 SV=8	0.113624511
**P97429**	Anxa4	Annexin A4 OS=Mus musculus OX=10090 GN=Anxa4 PE=1 SV=4	0.245396353
**P23116**	Eif3a	Eukaryotic translation initiation factor 3 subunit A OS=Mus musculus OX=10090 GN=Eif3a PE=1 SV=5	0.042145191
**P05213**	Tuba1b	Tubulin alpha-1B chain OS=Mus musculus OX=10090 GN=Tuba1b PE=1 SV=2	2.080117936
**P56959**	Fus	RNA-binding protein FUS OS=Mus musculus OX=10090 GN=Fus PE=1 SV=1	0.400415825
**P68369**	Tuba1a	Tubulin alpha-1A chain OS=Mus musculus OX=10090 GN=Tuba1a PE=1 SV=1	0.061917846
**P40142**	Tkt	Transketolase OS=Mus musculus OX=10090 GN=Tkt PE=1 SV=1	0.491205386
**P62908**	Rps3	40S ribosomal protein S3 OS=Mus musculus OX=10090 GN=Rps3 PE=1 SV=1	0.149882137
**P07356**	Anxa2	Annexin A2 OS=Mus musculus OX=10090 GN=Anxa2 PE=1 SV=2	1.057253961
**A0A0R4J0V5**	Polr2a	DNA-directed RNA polymerase subunit OS=Mus musculus OX=10090 GN=Polr2a PE=1 SV=1	0.004397156
**Q60598**	Cttn	Src substrate cortactin OS=Mus musculus OX=10090 GN=Cttn PE=1 SV=2	0.293246049

**P56480**	Atp5f1b	ATP synthase subunit beta, mitochondrial OS=Mus musculus OX=10090 GN=Atp5f1b PE=1 SV=2	0.629389637
**P14206**	Rpsa	40S ribosomal protein SA OS=Mus musculus OX=10090 GN=Rpsa PE=1 SV=4	0.734411361
**P14131**	Rps16	40S ribosomal protein S16 OS=Mus musculus OX=10090 GN=Rps16 PE=1 SV=4	0.259221127
**G3UVV4**	Hk1	Hexokinase 1, isoform CRA_f OS=Mus musculus OX=10090 GN=Hk1 PE=1 SV=1	0.027229461
**A0A075B5N9**	Igkv3-7	Immunoglobulin kappa variable 3-7 OS=Mus musculus OX=10090 GN=Igkv3-7 PE=4 SV=7	0.161966249
**Q6ZQ38**	Cand1	Cullin-associated NEDD8-dissociated protein 1 OS=Mus musculus OX=10090 GN=Cand1 PE=1 SV=2	0.016402436
**Q8BTU6**	Eif4a2	Eukaryotic initiation factor 4A-II OS=Mus musculus OX=10090 GN=Eif4a2 PE=1 SV=1	0.204043718
**A0A0R4J259**	Syncrip	Heterogeneous nuclear ribonucleoprotein Q OS=Mus musculus OX=10090 GN=Syncrip PE=1 SV=1	0.112714499
**Q9JHJ0**	Tmod3	Tropomodulin-3 OS=Mus musculus OX=10090 GN=Tmod3 PE=1 SV=1	0.062642681
**P19096**	Fasn	Fatty acid synthase OS=Mus musculus OX=10090 GN=Fasn PE=1 SV=2	0.069663529
**Q8R0W0**	Eppk1	Epiplakin OS=Mus musculus OX=10090 GN=Eppk1 PE=1 SV=2	0.016306673
**Q03265**	Atp5f1a	ATP synthase subunit alpha, mitochondrial OS=Mus musculus OX=10090 GN=Atp5f1a PE=1 SV=1	1.11084943
**P63017**	Hspa8	Heat shock cognate 71 kDa protein OS=Mus musculus OX=10090 GN=Hspa8 PE=1 SV=1	2.010544351
**P38647**	Hspa9	Stress-70 protein, mitochondrial OS=Mus musculus OX=10090 GN=Hspa9 PE=1 SV=3	0.243666272
**P62137**	Ppp1ca	Serine/threonine-protein phosphatase PP1-alpha catalytic subunit OS=Mus musculus OX=10090 GN=Ppp1ca PE=1 SV=1	0.016440529
**Q61656**	Ddx5	Probable ATP-dependent RNA helicase DDX5 OS=Mus musculus OX=10090 GN=Ddx5 PE=1 SV=2	0.248094644
**P10126**	Eef1a1	Elongation factor 1-alpha 1 OS=Mus musculus OX=10090 GN=Eef1a1 PE=1 SV=3	0.80731813
**P25444**	Rps2	40S ribosomal protein S2 OS=Mus musculus OX=10090 GN=Rps2 PE=1 SV=3	0.325641415
**E9Q390**	Myof	Myoferlin OS=Mus musculus OX=10090 GN=Myof PE=1 SV=2	0.068520723

**E9Q616**	Ahnak	AHNAK nucleoprotein (desmoyokin) OS=Mus musculus OX=10090 GN=Ahnak PE=1 SV=1	0.68425489
**D3YYM6**	Rps5	40S ribosomal protein S5 (Fragment) OS=Mus musculus OX=10090 GN=Rps5 PE=1 SV=1	0.299203453
**E9QAI5**	Cad	CAD protein OS=Mus musculus OX=10090 GN=Cad PE=1 SV=1	0.027160152
**F6TN80**	Srsf11	Serine/arginine-rich-splicing factor 11 (Fragment) OS=Mus musculus OX=10090 GN=Srsf11 PE=1 SV=1	0.019845667
**Q2KN98**	Specc1l	Cytospin-A OS=Mus musculus OX=10090 GN=Specc1l PE=1 SV=1	0.03616345
**C0HKE4**	Hist1h2ae	Histone H2A type 1-E OS=Mus musculus OX=10090 GN=Hist1h2ae PE=1 SV=1	1.111431414
**P62983**	Rps27a	Ubiquitin-40S ribosomal protein S27a OS=Mus musculus OX=10090 GN=Rps27a PE=1 SV=2	0.400198903
**P61979**	Hnrnpk	Heterogeneous nuclear ribonucleoprotein K OS=Mus musculus OX=10090 GN=Hnrnpk PE=1 SV=1	0.382744663
**Q9D8E6**	Rpl4	60S ribosomal protein L4 OS=Mus musculus OX=10090 GN=Rpl4 PE=1 SV=3	0.58050295
**Q9D8N0**	Eef1g	Elongation factor 1-gamma OS=Mus musculus OX=10090 GN=Eef1g PE=1 SV=3	0.373141921
**P12970**	Rpl7a	60S ribosomal protein L7a OS=Mus musculus OX=10090 GN=Rpl7a PE=1 SV=2	0.441445722

Figure 2F

UBE2O

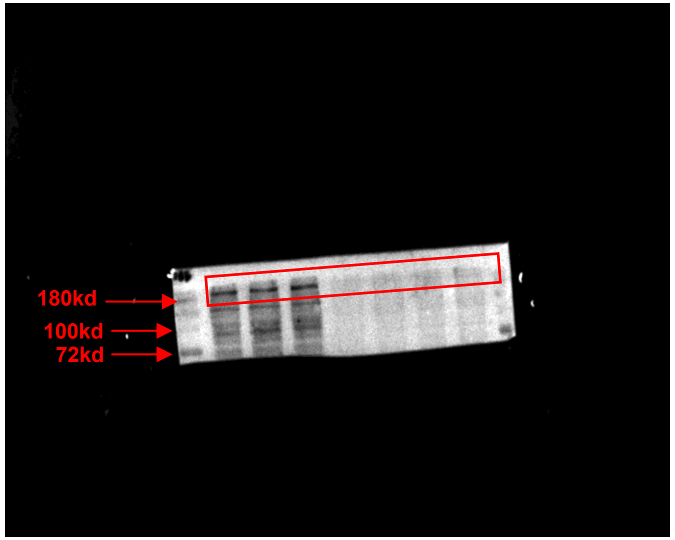

β-actin

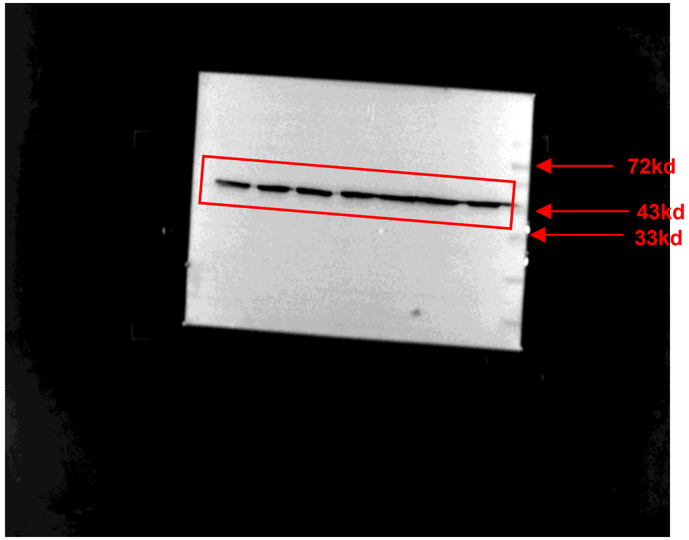

Figure 3B

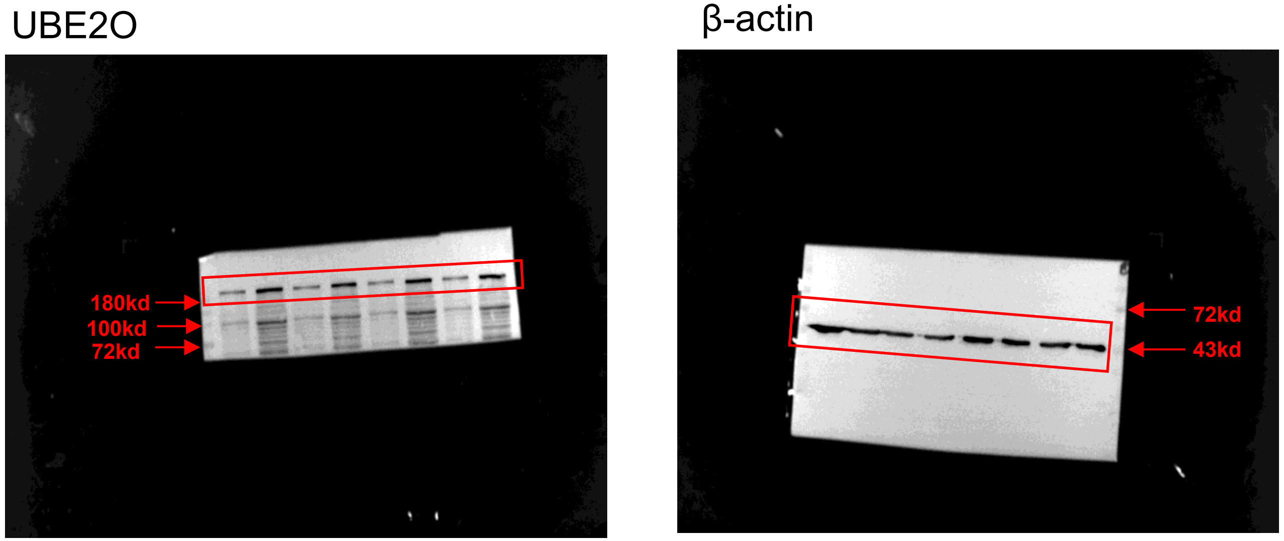

Figure 4B

UBE2O

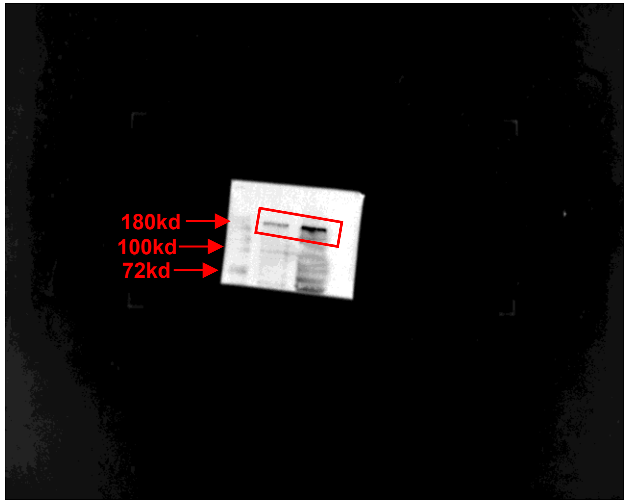

β-actin

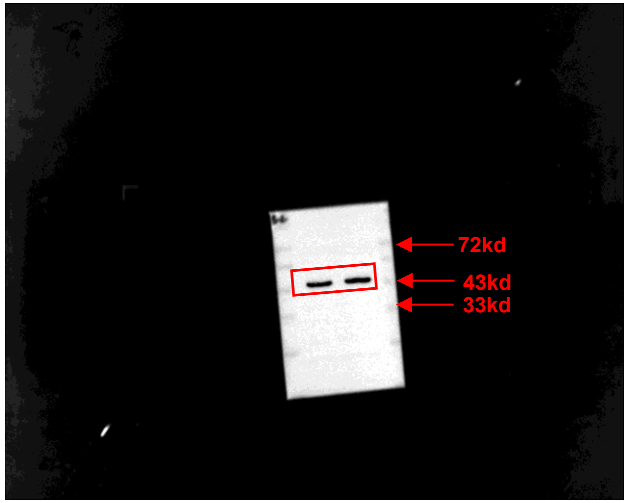

Figure 4G

UBE2O

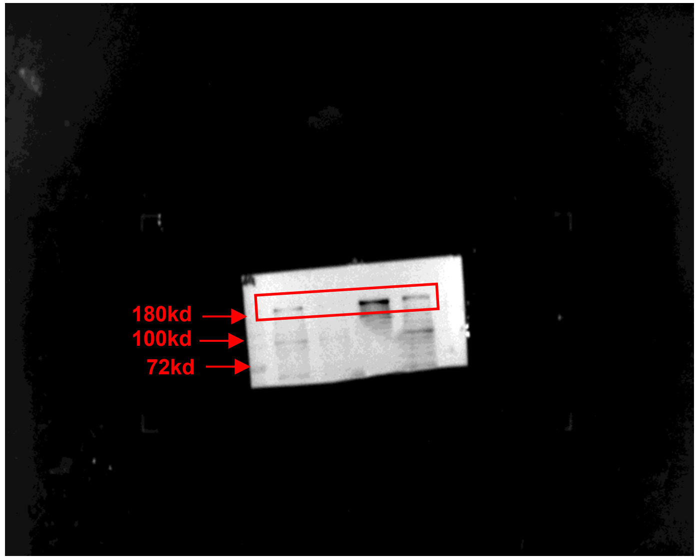

IL-6

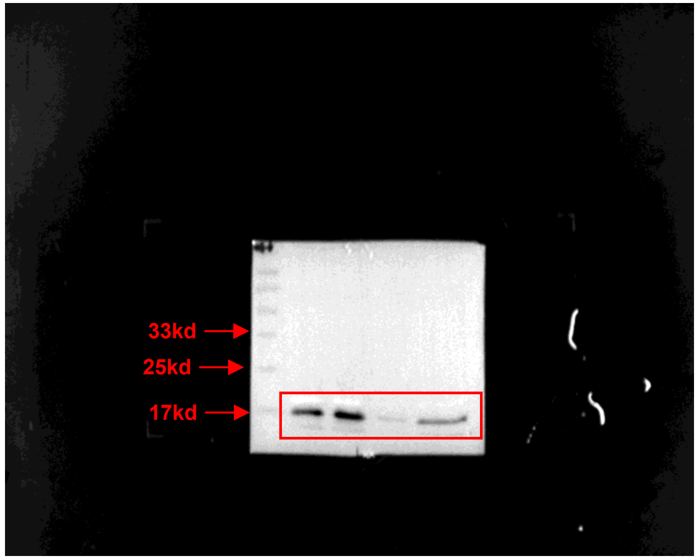

β-actin

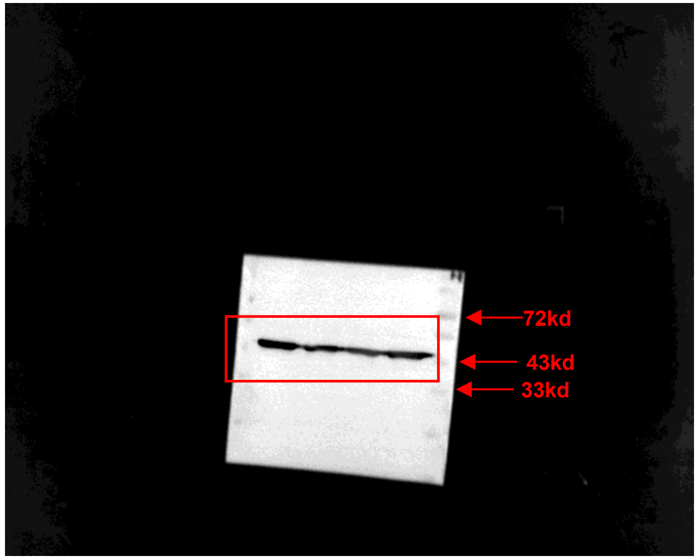

Figure 6B

UBE2O

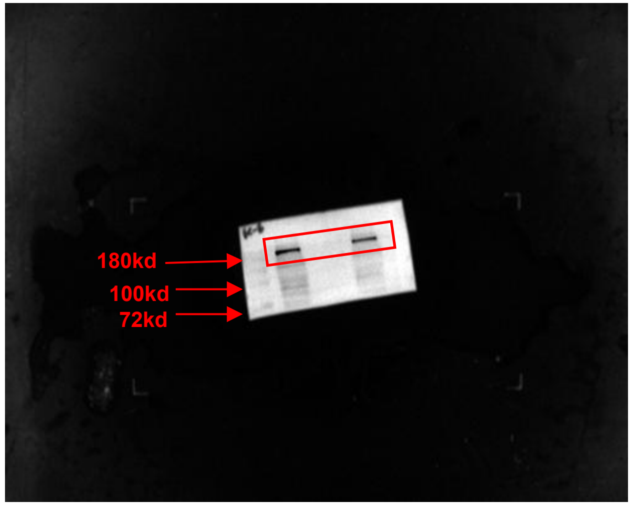

YBX1

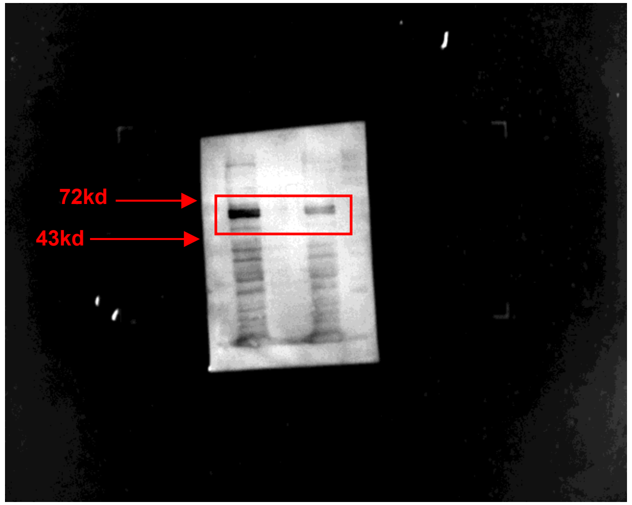

IL-6

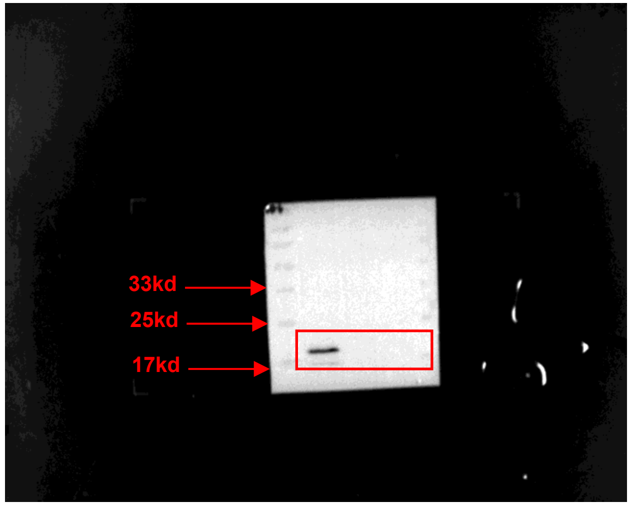

Figure 6C

YBX1

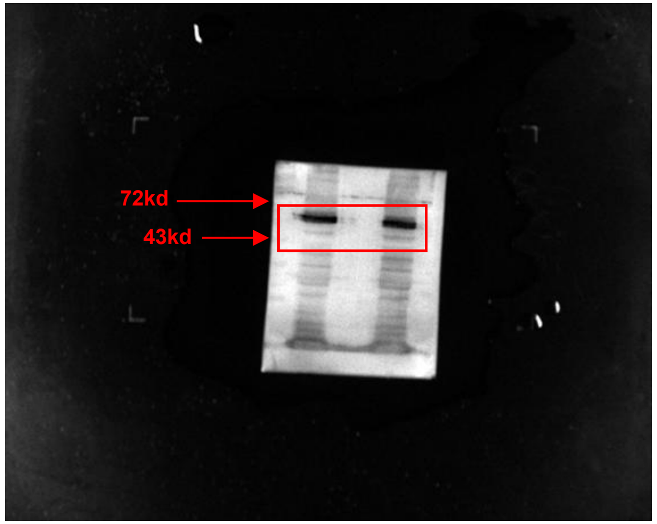

UBE2O

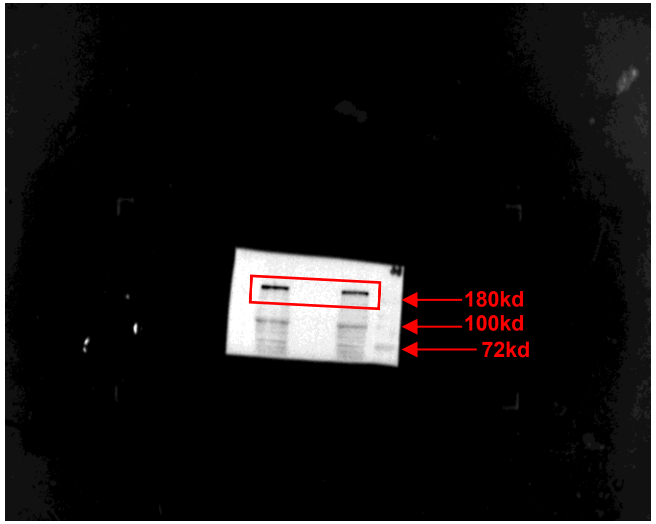

Figure 6D

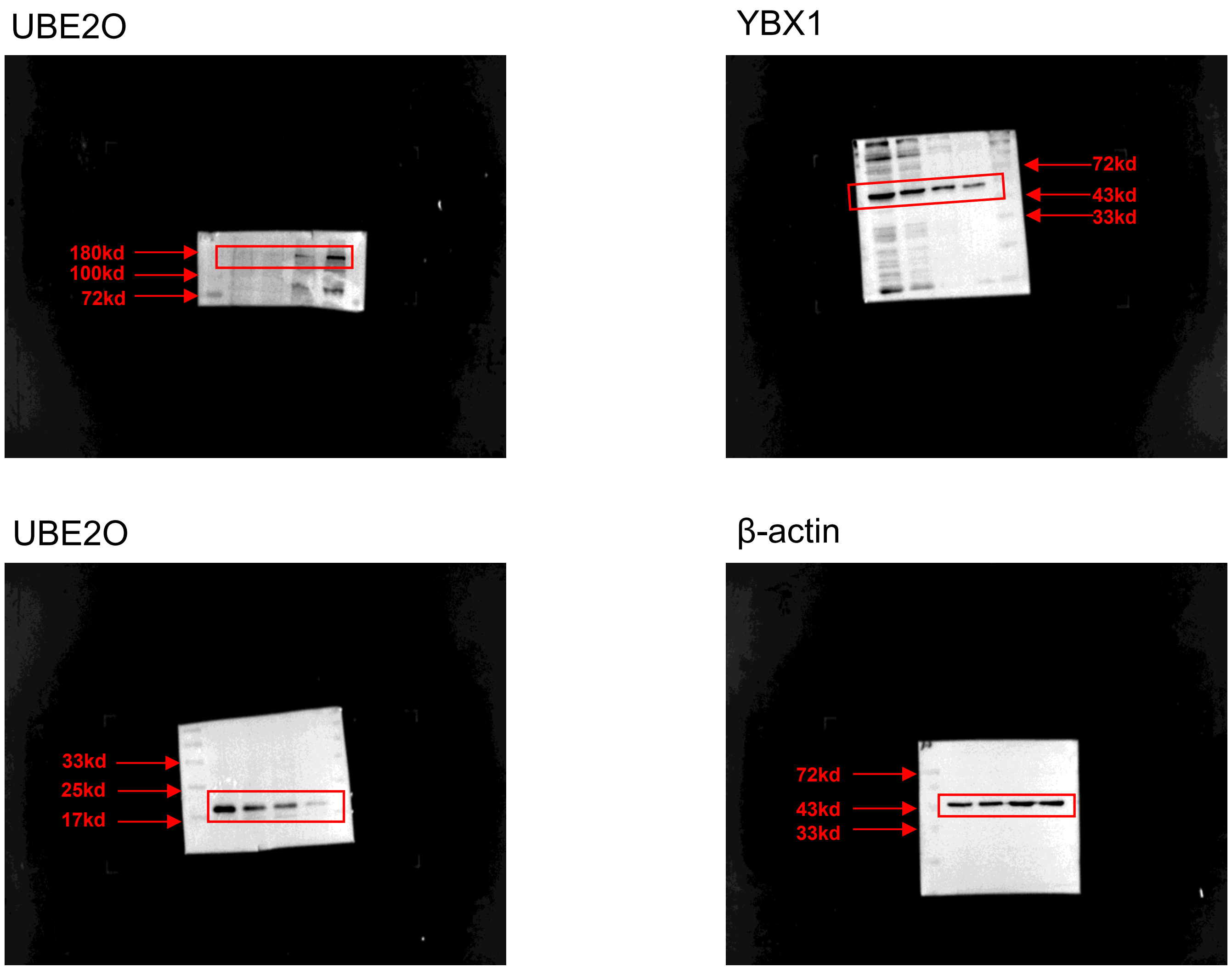
UBE2O
180kd
100kd
72kd
YBX1
72kd
43kd
33kd
UBE2O
33kd
25kd
17kd
β-actin
72kd
43kd
33kd

Figure 6E

UBE2O

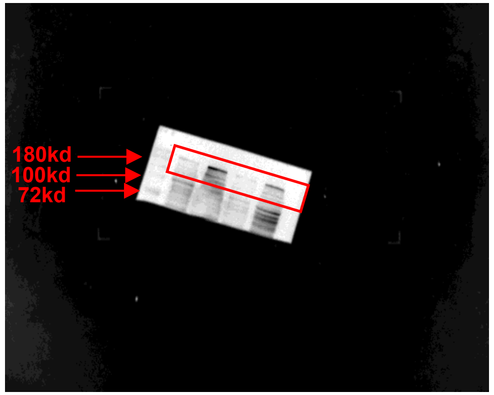

YBX1

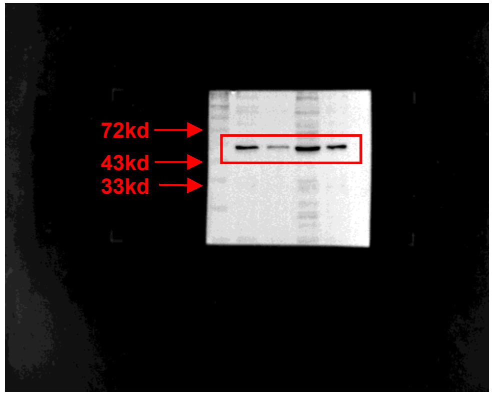

IL-6

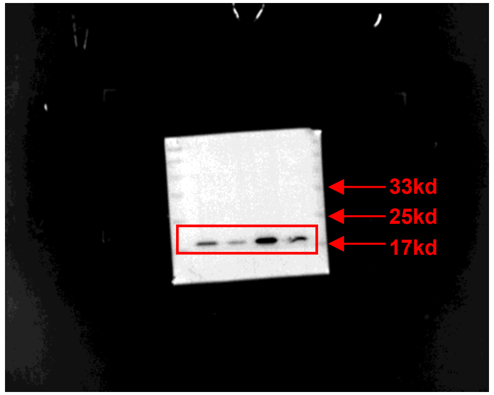

β-actin

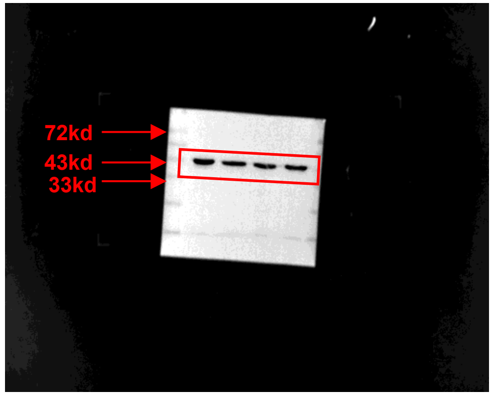

Figure 8E

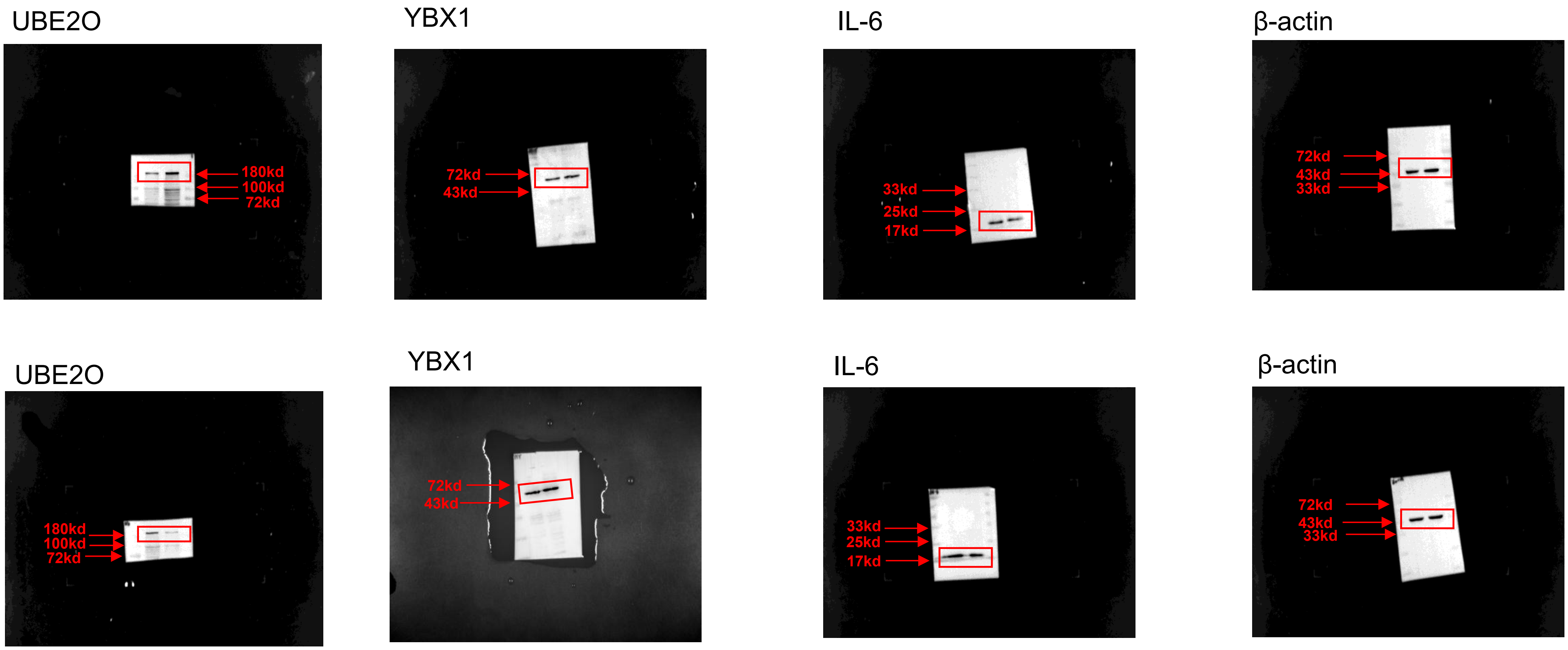
UBE2O
180kd
100kd
72kd
YBX1
72kd
43kd
IL-6
33kd
25kd
17kd
β-actin
72kd
43kd
33kd
UBE2O
180kd
100kd
72kd
YBX1
72kd
43kd
IL-6
33kd
25kd
17kd
β-actin
72kd
43kd
33kd

Figure 9A

YBX1

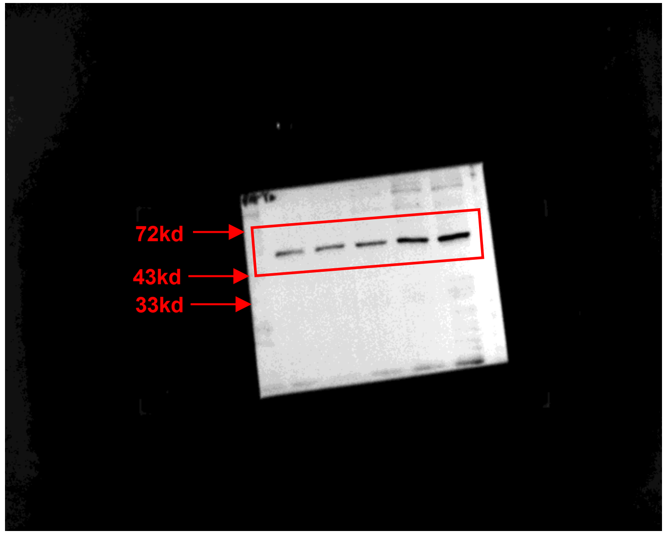

β-actin

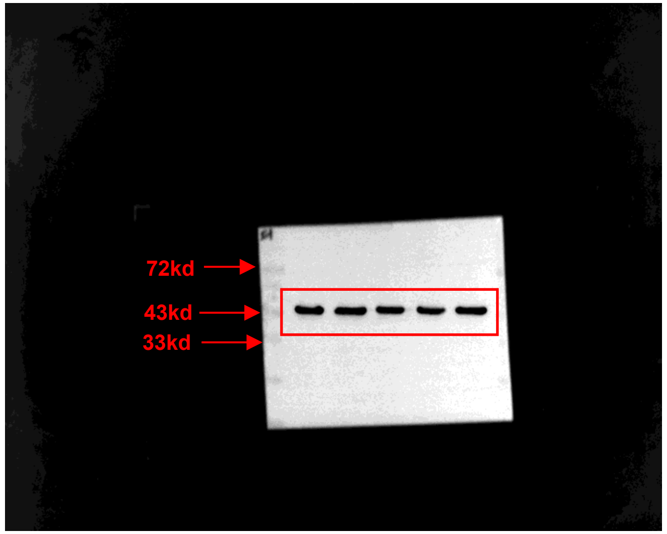

Figure 9B

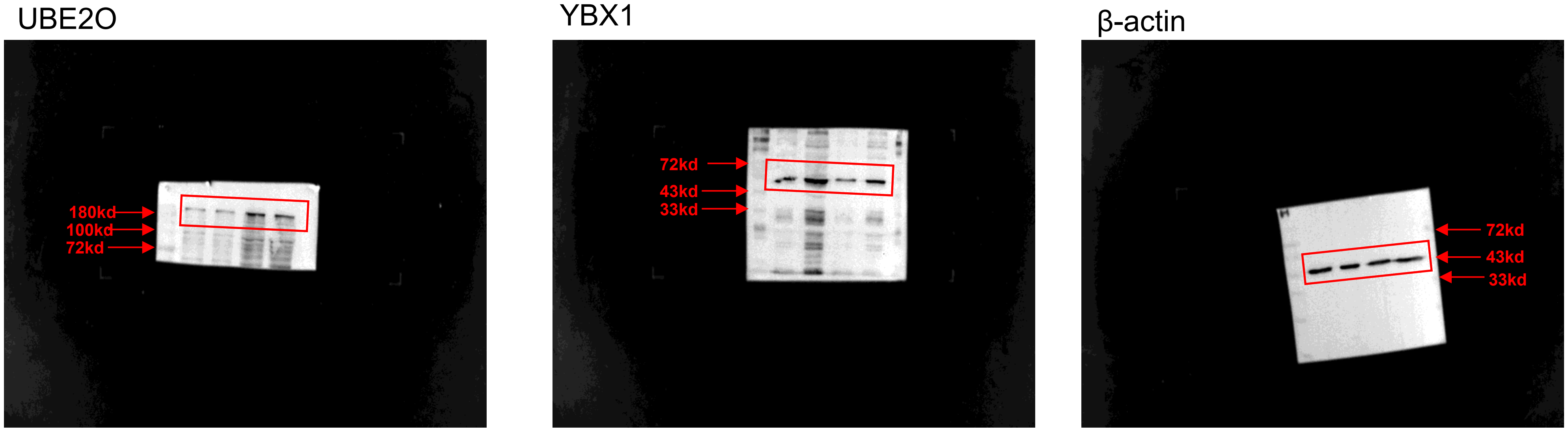
UBE2O
180kd
100kd
72kd
YBX1
72kd
43kd
33kd
β-actin
72kd
43kd
33kd

Figure 9C

UBE2O

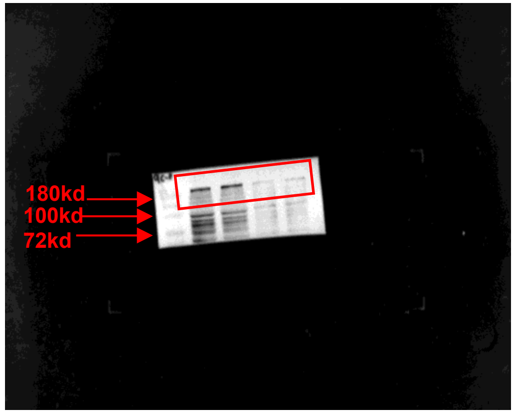

YBX1

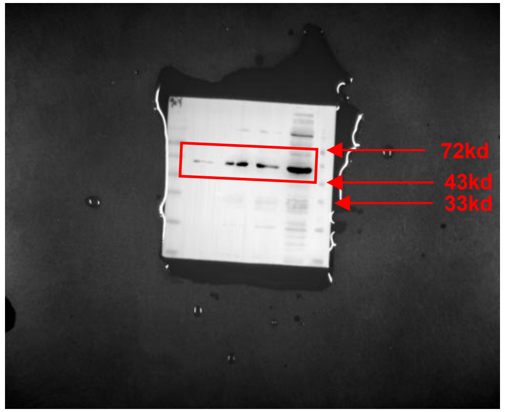

β-actin

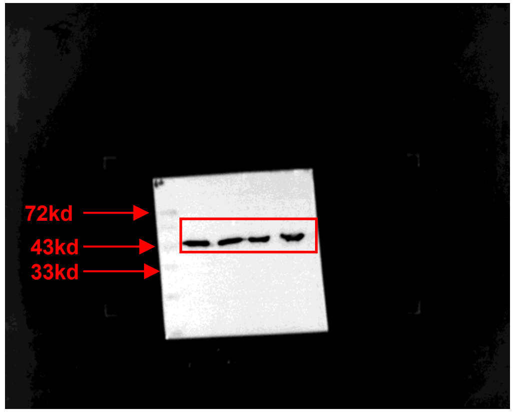

Figure 9D

UBE2O YBX1 β-actin

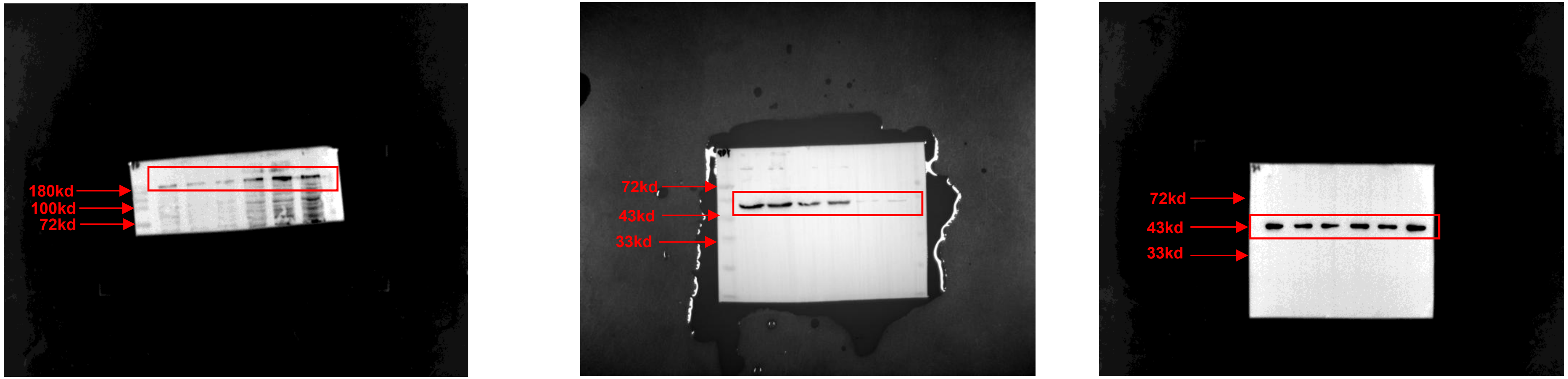

Figure 9E

UBE2O

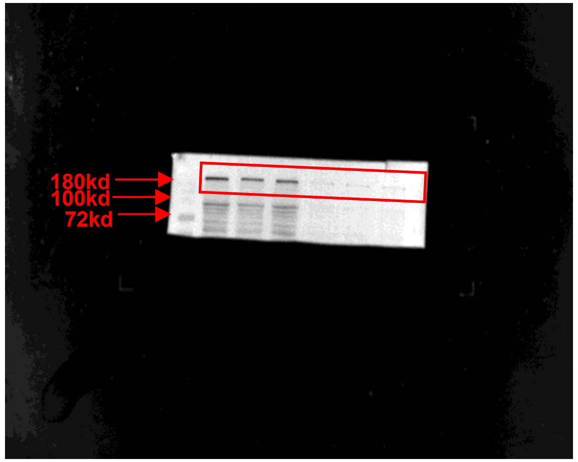

YBX1

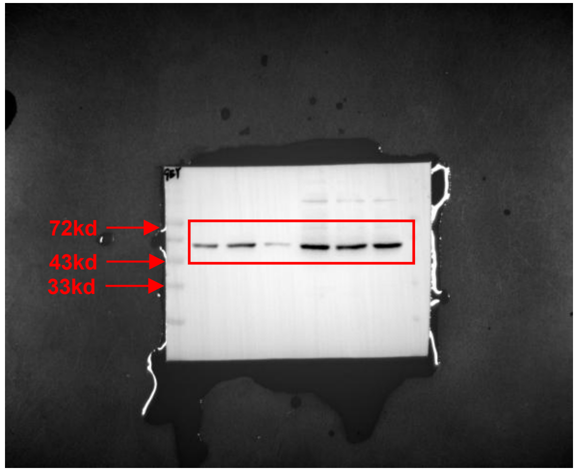

β-actin

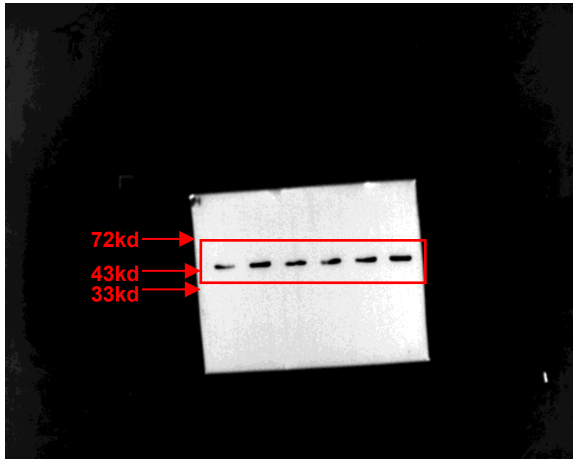

Figure 9F

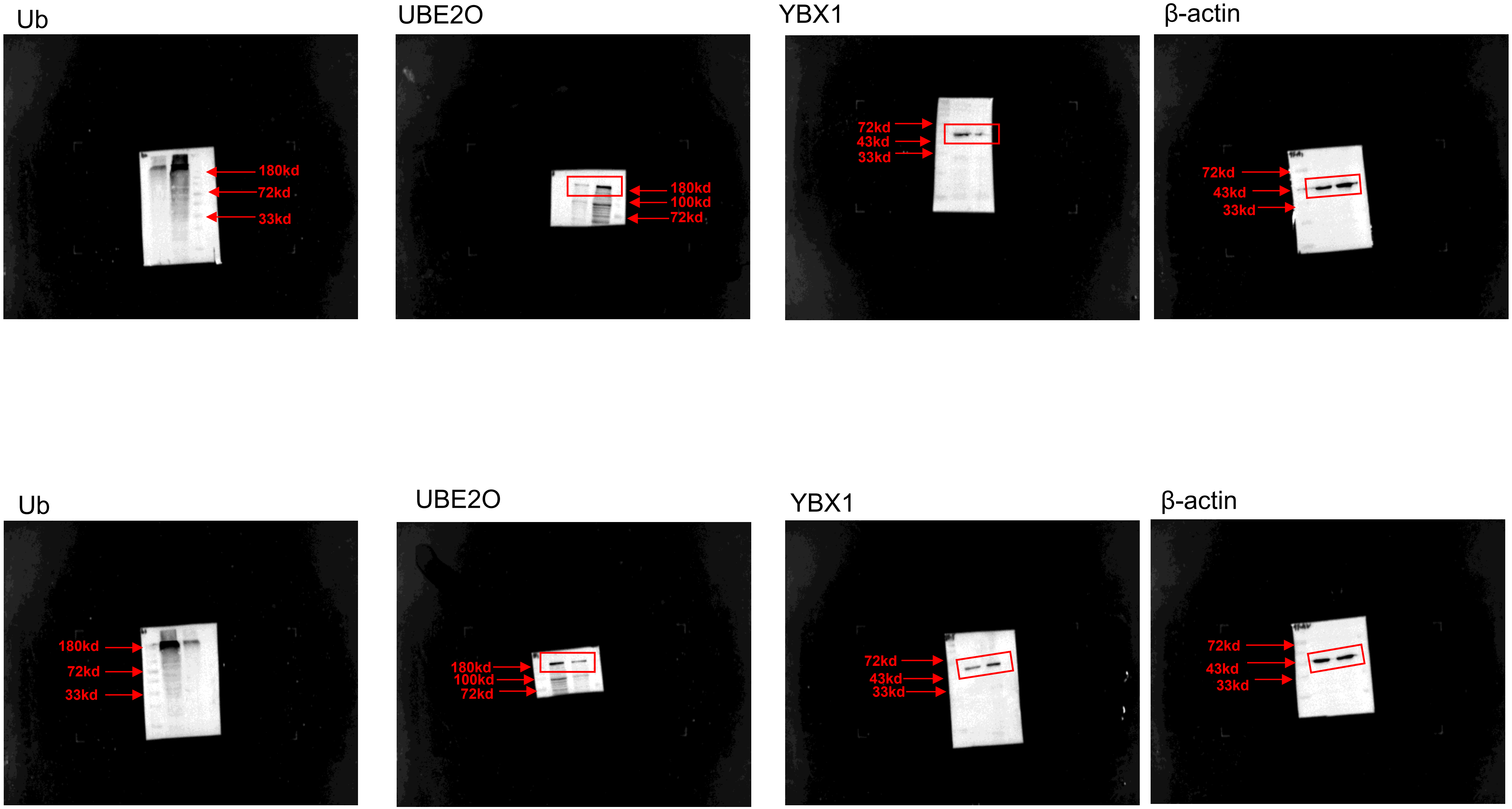
Ub
180kd
72kd
33kd
UBE2O
180kd
100kd
72kd
YBX1
72kd
43kd
33kd
β-actin
72kd
43kd
33kd
Ub
180kd
72kd
33kd
UBE2O
180kd
100kd
72kd
YBX1
72kd
43kd
33kd
β-actin
72kd
43kd
33kd

Figure 9H

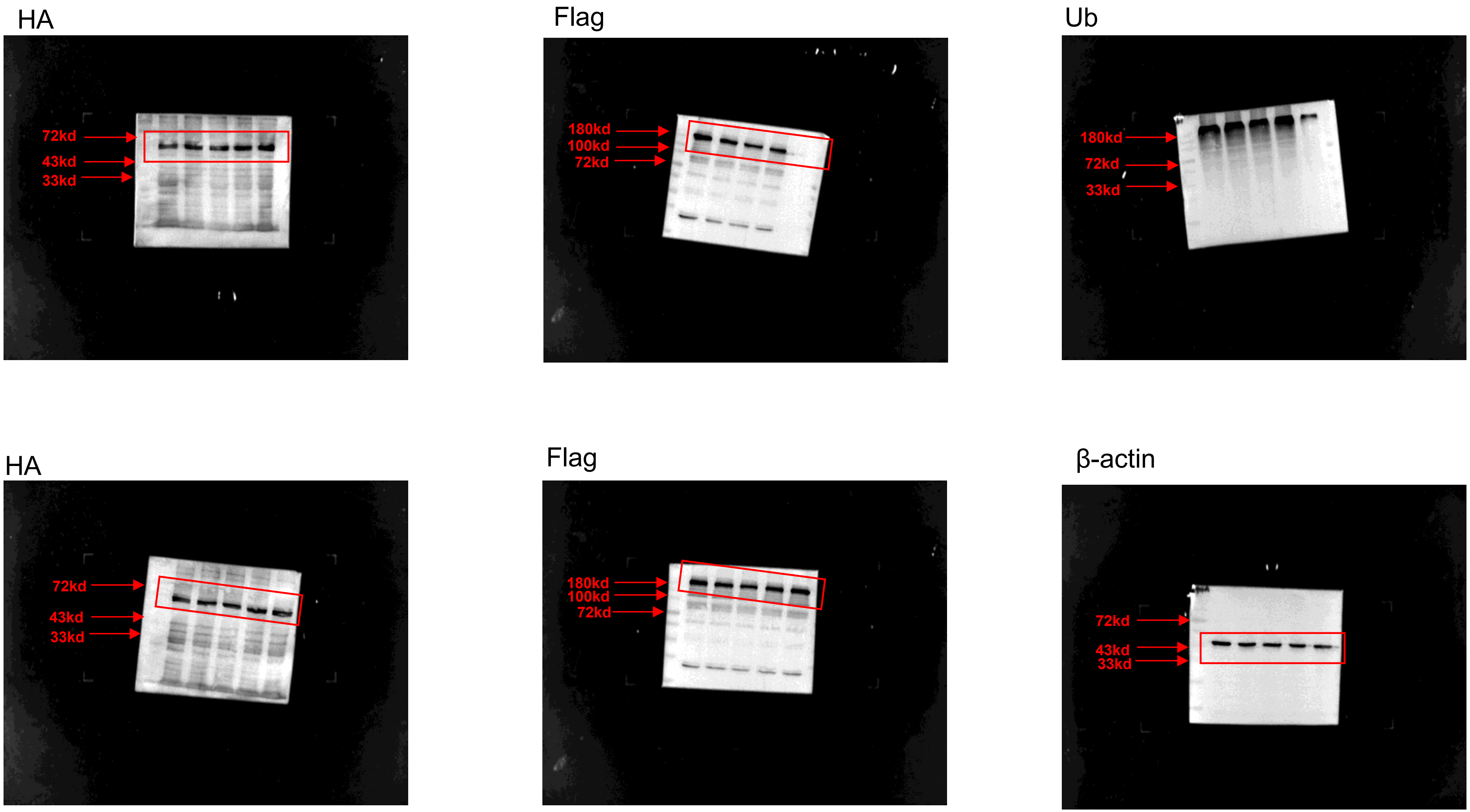
HA
72kd
43kd
33kd
Flag
180kd
100kd
72kd
Ub
180kd
72kd
33kd
HA
72kd
43kd
33kd
Flag
180kd
100kd
72kd
β-actin
72kd
43kd
33kd

Figure 10C

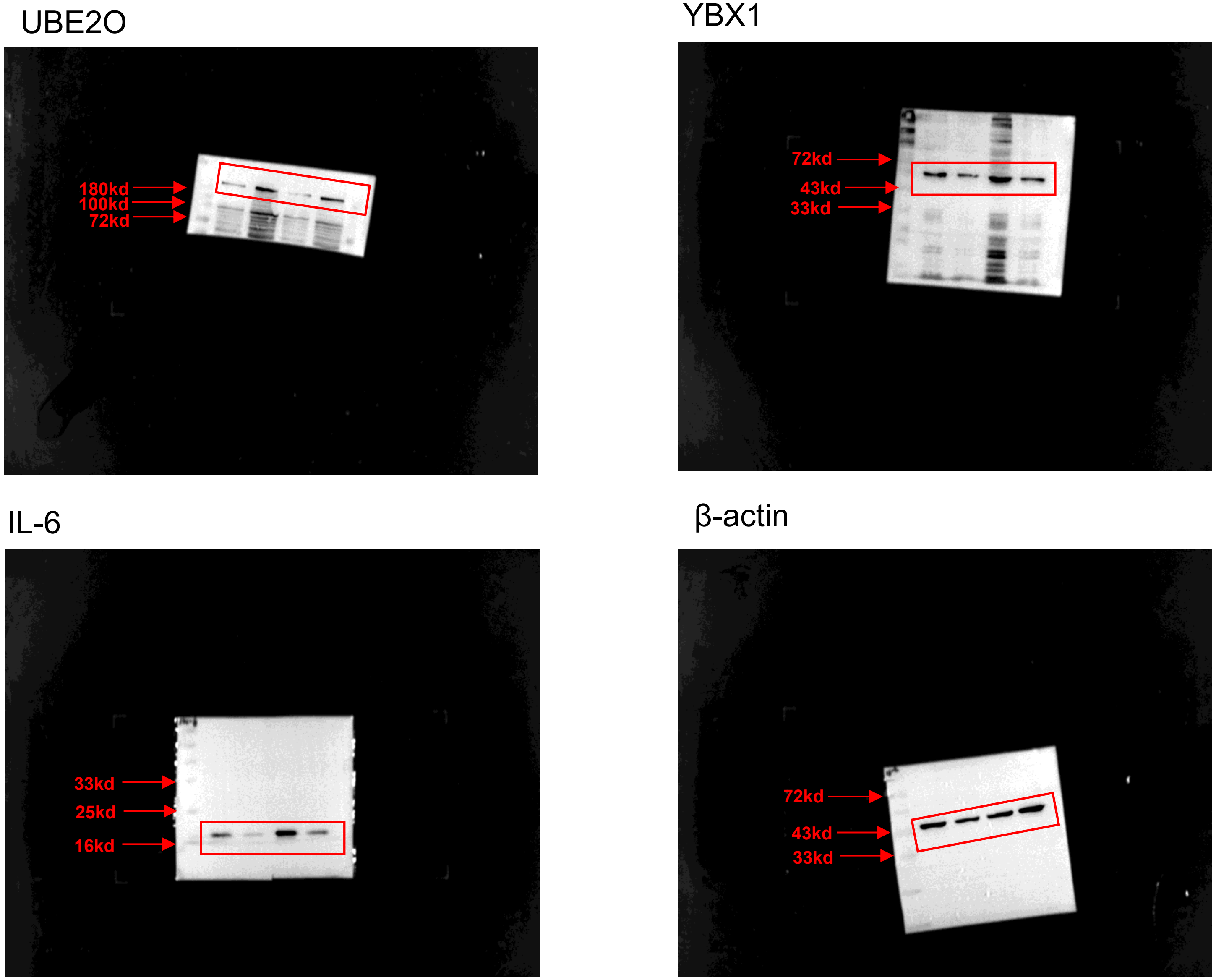
UBE2O
180kd
100kd
72kd
YBX1
72kd
43kd
33kd
IL-6
33kd
25kd
16kd
β-actin
72kd
43kd
33kd
